# A chromosomal-scale genome assembly of *Tectona grandis* reveals the importance of tandem gene duplication and enables discovery of genes in natural product biosynthetic pathways

**DOI:** 10.1093/gigascience/giz005

**Published:** 2019-01-30

**Authors:** Dongyan Zhao, John P Hamilton, Wajid Waheed Bhat, Sean R Johnson, Grant T Godden, Taliesin J Kinser, Benoît Boachon, Natalia Dudareva, Douglas E Soltis, Pamela S Soltis, Bjoern Hamberger, C Robin Buell

**Affiliations:** 1 Department of Plant Biology, Michigan State University, 612 Wilson Road, East Lansing, MI 48824, USA; 2 Department of Biochemistry and Molecular Biology, Michigan State University, 603 Wilson Road, East Lansing, MI 48824, USA; 3 Department of Pharmacology and Toxicology, Michigan State University, 1355 Bogue Street, East Lansing, MI 48824, USA; 4 Florida Museum of Natural History, University of Florida, 1659 Museum Road, Gainesville, FL 32611, USA; 5 Department of Biology, University of Florida, 876 Newell Drive, Gainesville, FL 32611, USA; 6 Department of Biochemistry, Purdue University, 625 Agriculture Mall Drive, West Lafayette, IN 47907, USA; 7 Plant Resilience Institute, Michigan State University, 612 Wilson Road, East Lansing, MI 48872, USA; 8 MSU AgBioResearch, Michigan State University, 446 West Circle Drive, East Lansing, MI 48872, USA

**Keywords:** teak, chromosomal-scale assembly, terpene synthases, tandem-duplicated genes

## Abstract

**Background:**

Teak, a member of the Lamiaceae family, produces one of the most expensive hardwoods in the world. High demand coupled with deforestation have caused a decrease in natural teak forests, and future supplies will be reliant on teak plantations. Hence, selection of teak tree varieties for clonal propagation with superior growth performance is of great importance, and access to high-quality genetic and genomic resources can accelerate the selection process by identifying genes underlying desired traits.

**Findings:**

To facilitate teak research and variety improvement, we generated a highly contiguous, chromosomal-scale genome assembly using high-coverage Pacific Biosciences long reads coupled with high-throughput chromatin conformation capture. Of the 18 teak chromosomes, we generated 17 near-complete pseudomolecules with one chromosome present as two chromosome arm scaffolds. Genome annotation yielded 31,168 genes encoding 46,826 gene models, of which, 39,930 and 41,155 had Pfam domain and expression evidence, respectively. We identified 14 clusters of tandem-duplicated terpene synthases (TPSs), genes central to the biosynthesis of terpenes, which are involved in plant defense and pollinator attraction. Transcriptome analysis revealed 10 TPSs highly expressed in woody tissues, of which, 8 were in tandem, revealing the importance of resolving tandemly duplicated genes and the quality of the assembly and annotation. We also validated the enzymatic activity of four TPSs to demonstrate the function of key TPSs.

**Conclusions:**

In summary, this high-quality chromosomal-scale assembly and functional annotation of the teak genome will facilitate the discovery of candidate genes related to traits critical for sustainable production of teak and for anti-insecticidal natural products.

## Data Description

### Introduction

**Figure 1:**
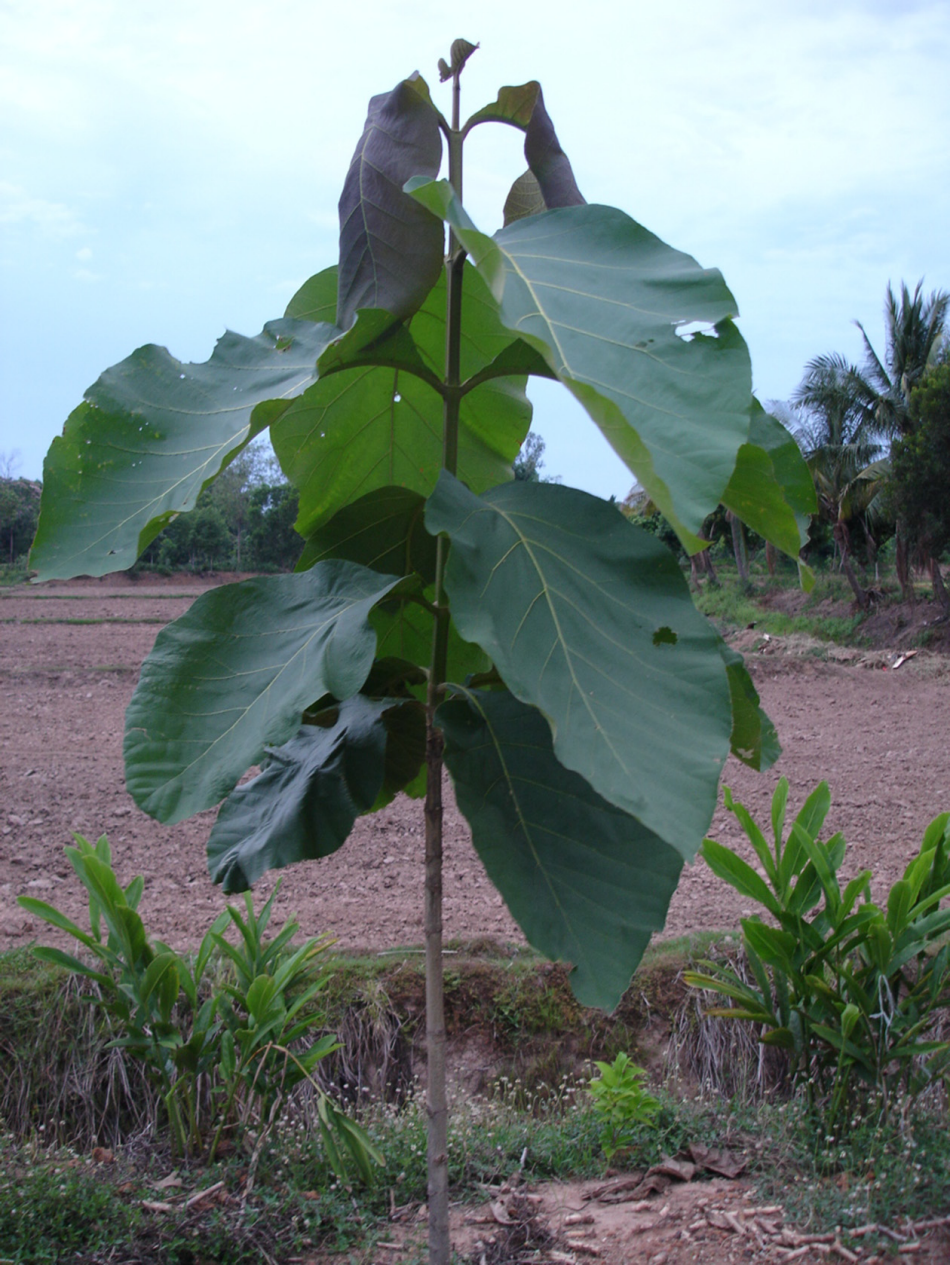
A young teak tree. Photo taken by Phong Ek [CC BY 2.0 (https://creativecommons.org/licenses/by/2.0)], via Wikimedia Commons

Teak (*Tectona grandis* L.f.; 2*n* = 2*x* = 36), a member of the angiosperm family Lamiaceae, produces timber of high value due to its durability, hardness, appearance, and resistance to biotic and abiotic stresses (Fig. 1). Teak is one of the most expensive hardwoods in the world, with an average price for high-quality logs ranging from $600 to $1,000/m^3^ [1]. High demand coupled with deforestation have caused a decrease in natural teak forests, and future supplies will be reliant on teak plantations. Hence, selection of teak tree varieties with superior growth performance for clonal propagation is of great importance, and access to high-quality genetic and genomic resources can accelerate the selection process by identifying genes underlying desired traits. The only available genome assembly for teak (hereafter referred to as the “released assembly”) was completed using short-reads and low-coverage (7x) nanopore long reads [2]. While improved compared to other short-read assembled plant genomes, the released assembly is still highly fragmented, comprising 2,993 scaffolds with the maximum and N50 scaffold length of 1.7 Mb and 358 kbp, respectively.

### DNA extraction and genome sequencing

Teak seeds were obtained from Sheffield's Seed Company [3]. High-molecular-weight DNA was extracted from young leaves of a 2-week-old plant grown in the greenhouse using a modified cetyl trimethylammonium bromide method [4]. Long read sequencing was done using Pacific Biosciences (PacBio) RSII and Sequel single-molecule sequencers at the University of Delaware Sequencing & Genotyping Center. Briefly, SMRTbell DNA libraries were constructed from genomic DNA using the SMRTbell Template Prep Kit 1.0-SPv3 as per the manufacturer's instructions (Pacific Biosciences, Menlo Park, CA). The library was size selected using the BluePippin size-selection system and protocol for 15 Kbp size selection (Sage Science, Amherst, MA). Following size selection, the average library fragment size was 25 kb based on the Fragment Analyzer sizing profile (Advanced Analytical Technologies, Arkeny, IA). The library was sequenced for 6 hours on 10 single molecule real-time (SMRT) sequencing cells using P6-C4 chemistry on the PacBio RS II instrument (Pacific Biosciences) and 10 hours on 4 SMRT cells using 2.0 sequencing chemistry on the PacBio Sequel instrument (Pacific Biosciences). A total of ∼4.7 million PacBio long reads were generated, which is ∼104x coverage of the estimated 325 Mbp teak genome. Additionally, whole-genome short-read sequencing libraries were generated using Illumina TruSeq Nano DNA Library Preparation Kit (Cat. No. FC-121-4001) and sequenced to 150-nt paired end reads on Illumina HiSeq 4000.

### Genome assembly and quality assessment

**Figure 2:**
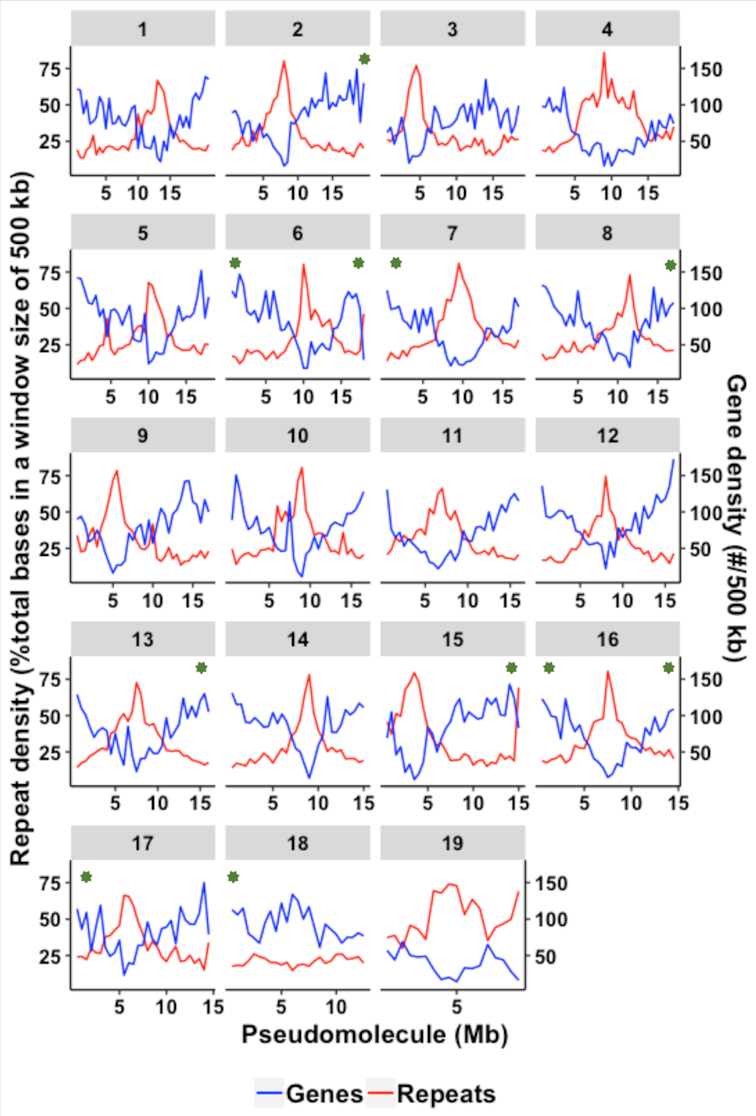
Gene and repeat density across the 19 pseudomolecules in the assembly. Green asterisks denote telomere tracks.

The raw reads were error-corrected using Canu (Canu, RRID:SCR_015880) v1.6 [5] (canu -correct) and trimmed (canu -trim) for low-quality bases, and reads ≥1 kb were used to generate the initial assembly (canu -assemble) with a correctedErrorRate of 0.09%. The assembly consists of 1,474 contigs with a total length of 338 Mbp, 20 Mbp larger than the released assembly (Tables 1 and 2). The initial assembly was polished using the raw PacBio reads using Arrow in the SMRT Analysis package v5.0.1.9585 [6], followed by three rounds of error correction with 643.7 million Illumina short reads (570x coverage, Table 3) using Pilon (Pilon, RRID:SCR_014731) v1.13 [7]. A Dovetail Hi-C library was prepared as described previously [8]. The resulting library had a double restriction site signature, where four non-genomic bases were introduced. The initial PacBio assembly, shotgun reads, and Dovetail Hi-C library reads were used as input data for scaffolding using HiRise [9]. Shotgun and Dovetail Hi-C library sequences were aligned to the initial assembly using a SNAP read mapper [10] where the four non-genomic bases were deleted prior to the mapping. The separation of aligned Dovetail Hi-C read pairs was analyzed by HiRise to produce a likelihood model for genomic distance between read pairs, and the model was used to identify and break putative mis-joins, to score prospective joins, and to make joins above a threshold. The Hi-C scaffolding resulted in 936 scaffolds (referred to as “improved assembly,” hereafter), with an N50 scaffold size of 18.5 Mbp, which is a 46x improvement of genome contiguity over the released assembly (Tables 1 and 2). The 19 largest scaffolds (minimum length of 8.6 Mbp) represented 90% of the assembled 338 Mbp genome; of the 18 teak chromosomes, we generated 17 near-complete pseudomolecules with one chromosome present as two chromosome arm scaffolds (Fig. 2). The completeness of our improved assembly was also demonstrated by the presence of tandem tracts of the telomere repeat sequence in 9 of the 19 pseudomolecules; 2 pseudomolecules contained telomere tracks at both ends (Fig. 2). A tandem array of 5S rRNA sequence (135 copies with each at 496 bp) was found in pseudomolecule 10 spanning >67.5 kbp, highlighting the power of long reads in resolving highly repetitive sequences. Around 98% of the whole genome shotgun reads aligned to the improved assembly, of which, 94%–98% of the reads were properly paired (Table 3). The representation of genic sequences in our improved assembly was confirmed by detection of 94.4% of the Benchmarking Universal Single-Copy Orthologs (BUSCO, RRID:SCR_015008) v2.0 [11]; Complete:92.3%[Single-copy:82.4%, Duplicated:9.9%], Fragmented:2.1%, Missing:5.6%, Total BUSCO groups searched:1440; Supplementary Table S1) and by alignment of 89%–93% of transcriptome reads from publicly available RNA sequencing (RNA-seq) datasets derived from diverse tissues of other teak accessions [12] (National Center for Biotechnology Information [NCBI] Sequence Read Archive [SRA] SRP059970; Supplementary Table S2).

**Table 1:** Metrics of contigs and scaffolds of the current assembly

	Initial assembly using PacBio reads (contigs)	Assembly after Hi-C scaffolding (scaffolds)
Total sequences	1,474	936
Total size (bp)	338,318,549	338,300,341
Maximum sequence size (bp)	21,267,566	20,661,910
Minimum sequence size (bp)	1,168	1,168
N50 sequence size (bp)	3,749,470	16,483,567
N90 sequence size (bp)	52,675	463,203
Average sequence size (bp)	229,524	361,432

**Table 2:** Cumulative size of contigs and scaffolds of the current assembly

Initial assembly using PacBio reads (contigs)			
Contig size	Total size (bp)	%Total assembly	Number of Contigs
≥1 Mbp	248,187,558	73.37	64
≥0.5 Mbp	267,412,682	79.06	91
≥0.1 Mbp	291,028,790	86.04	198
≥0.05 Mbp	305,851,391	90.42	420
Assembly after Hi-C scaffolding (scaffolds)			
Scaffold size	Total size (bp)	%Total assembly	Number of Scaffolds
≥1 Mbp	304,435,280	89.99	19
≥0.5 Mbp	304,435,280	89.99	19
≥0.1 Mbp	308,724,809	91.26	41
≥0.05 Mbp	314,467,503	92.96	134

**Table 3:** Whole genome shotgun reads

Sample name	NCBI SRA run ID	QC-passed reads	Mapped	Properly paired out of total reads
Teak_TruSeq_01	SRR7984127	168,566,966	165,783,328 (98.35%)	163,390,358 (97.40%)
Teak_TruSeq_02	SRR7984127	188,504,116	185,541,771 (98.43%)	182,934,854 (97.15%)
TEC_AA_01	SRR7984129	371,978,214	364,473,434 (97.98%)	357,722,188 (96.65%)
TEC_AA_02	SRR7984129	394,477,964	386,545,305 (97.99%)	379,620,884 (96.72%)
TEC_AB_01	SRR7984130	89,116,777	87,087,277 (97.72%)	84,001,838 (94.93%)
TEC_AB_02	SRR7984130	81,436,054	79,540,000 (97.67%)	76,733,986 (94.89%)

### Genome annotation

A custom repeat library (CRL) was generated for teak by running RepeatModeler (RepeatModeler, RRID:SCR_015027) v1.0.8 [13], excluding protein-coding genes using ProtExcluder (v1.1) [14] and adding the Viridiplantae RepBase repeats [15]. The improved assembly was masked with the CRL using RepeatMasker (RepeatMasker, RRID:SCR_012954) v4.0.6 with default parameters [16], which revealed that 32.02% of the improved assembly was identified as repetitive sequence, 3-fold more compared to that reported in the released assembly (11%). To generate transcript evidence for genome annotation, raw RNA-seq reads from a previous study were downloaded from NCBI (SRA SRP059970), and adapters and low-quality bases were removed using Cutadapt (v1.8.1) [17] requiring a minimum base quality of 20 and minimum size of 20-nt. The processed reads were aligned to the improved assembly using TopHat2 (v2.0.13) [18] with default parameters. Genome-guided transcript assemblies for each aligned RNA-seq library were created using Trinity (Trinity, RRID:SCR_013048) v2.2.0 [19] using the default parameters. Gene models were predicted using Augustus (Augustus: Gene Prediction, RRID:SCR_008417) v3.1 [20] by first training Augustus with the leaf RNA-seq alignments, then generating gene predictions on the hard-masked genome. The predicted gene models were refined by running PASA2 v2.1.0 [21] using the genome-guided transcript assemblies and two rounds of annotation comparison. Genes of interest (e.g., terpene synthases as described below) were manually curated using Apollo v1.11.8 [22]. The final working set of annotations was comprised of 31,168 loci and 46,826 gene models. Functional annotation was assigned using Basic Local Alignment Search Tool (BLAST) [23] searches against the *Arabidopsis thaliana* (L.) Heynh annotation (TAIR10) [24] and Swiss-Prot plant proteins (downloaded on 17 November 2016), and a search against Pfam v31 [25] using HMMER v3.1b2 [26] with a cutoff of 1e-5. A high confidence subset of the working gene model set was identified by identifying models with an FPKM (fragments per kilobase of exon model per million reads mapped, a normalized estimation of gene expression abundance) >0 in any of the RNA-seq libraries or a match in Pfam (v31). The high confidence gene model set is comprised of 41,155 gene models and 39,930 loci.

### Detection of whole genome duplication events

Whole-genome duplications (WGDs) can contribute to genetic innovations underlying chemical defense against co-evolving insect herbivores, as exemplified by evidence from studies of other plant groups (e.g., Brassicales [27]). To infer WGD events in teak, we used the DupPipe pipeline with default settings [28] to analyze coding sequences representing the longest isoforms of genes (Supplemental Information). Gaussian mixture models predicted three components within the observed *Ks* distribution of teak, with mean values at *K_S_* = 0.22, 0.60, 1.36 (Supplementary Fig. S1A). These components were further compared with results from a SiZer analysis [29] (implemented with the “multimode” R statistical package [30]), which distinguishes true data features from noise by testing for significant increases or decreases, or no significant changes across an observed *K_S_* distribution at various bandwidths (Supplemental Information). Of the three peaks identified with mixture models, only a peak at *K_S_* = 0.60 was corroborated as a significant feature by a SiZer analysis (Supplementary Fig. S1B), providing evidence for at least one WGD event in teak. Whether or not this WGD event is lineage-specific or shared by other Lamiaceae is a subject of active research.

### The phenylpropanoid pathway genes and their expression

**Figure 3:**
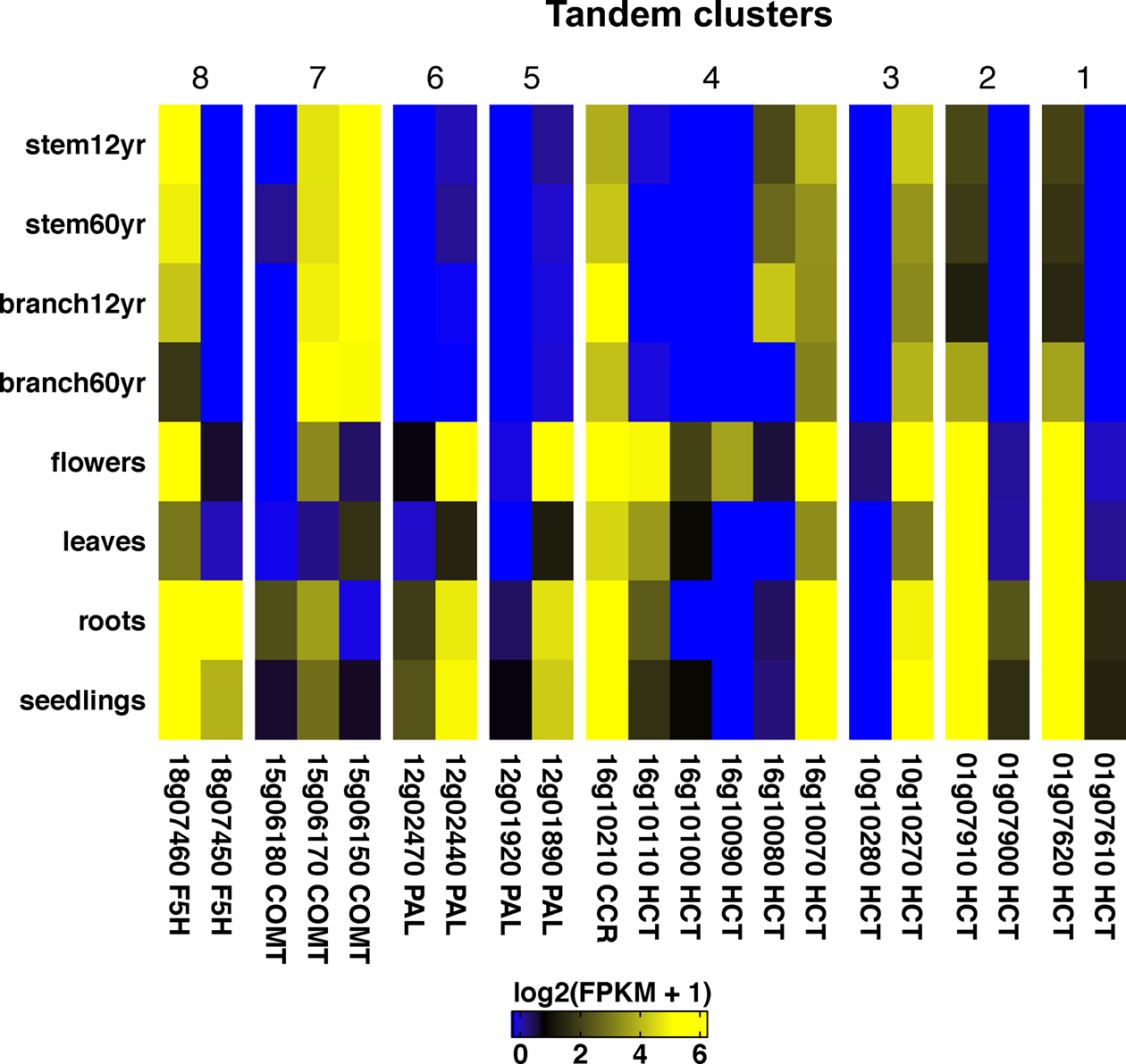
Differential expression of tandem copies of genes in lignin biosynthetic pathway. stem12yr: stem secondary xylem of a 12-year-old teak tree; stem60yr: stem secondary xylem of a 60-year-old teak tree; branch12yr: branch secondary xylem of a 12-year-old teak tree; branch60yr: branch secondary xylem of a 60-year-old teak tree.

Teak is known for strong wood, and we were able to identify all of the genes involved in the phenylpropanoid pathway that leads to lignin formation (Supplementary Table S3). Using phenylpropanoid pathway genes in *A. thaliana* [31] as bait, the corresponding candidate genes in teak were identified based on orthology analysis between teak and *A. thaliana* using OrthoFinder v2.0 with default parameters [32]. The phenylpropanoid pathway genes are often found in physical clusters [33] and we defined physical clusters of genes if: (1) there were no more than 10 genes in between on a single pseudomolecule and (2) the pairwise gene distance was less than 100 kbp. Notably, 4 of the 11 core genes in the phenylpropanoid pathway were present in tandem copies, with shikimate O-hydroxycinnamoytransferase (HCT) having three tandem clusters of two copies each and one cluster of five copies (Fig. 3). To better understand the potential function of these tandem gene clusters, normalized estimation of expression abundances (FPKM) of the annotated teak genes were quantified for the RNA-seq experiments (SRA SRP059970) described above using Cufflinks (Cufflinks, RRID:SCR_014597) v2.2.1 with default parameters [34]. Except for the 12-year-old branch (replicate 1 showed low correlation with other branch samples), the two biological replicates for other branch and stem samples showed high correlations (*r*>0.94, *P*<0.0001, Supplementary Table S4) of gene expression levels; therefore, replicate 2 for the 12-year-old branch and one replicate for other woody tissues were used for downstream analyses. For 20 of the 45 genes in the phenylpropanoid pathway, clear neofunctionalization at the expression level was observed for ferulate 5-hydroxylases, caffeic acid O-methyltransferases, phenylalanine ammonia lyase, and HCT. Interestingly, cinnamyl CoA reductase (CCR), which catalyzes the first committed step of the lignin-specific branch, was in a physical cluster with five copies of HCT; within this physical cluster, only one of the five HCT genes (Tg16g10070) and CCR (Tg16g10210) were constitutively expressed in all tissues (Fig. 3).

### Identification of terpene synthases and functional verification

**Figure 4:**
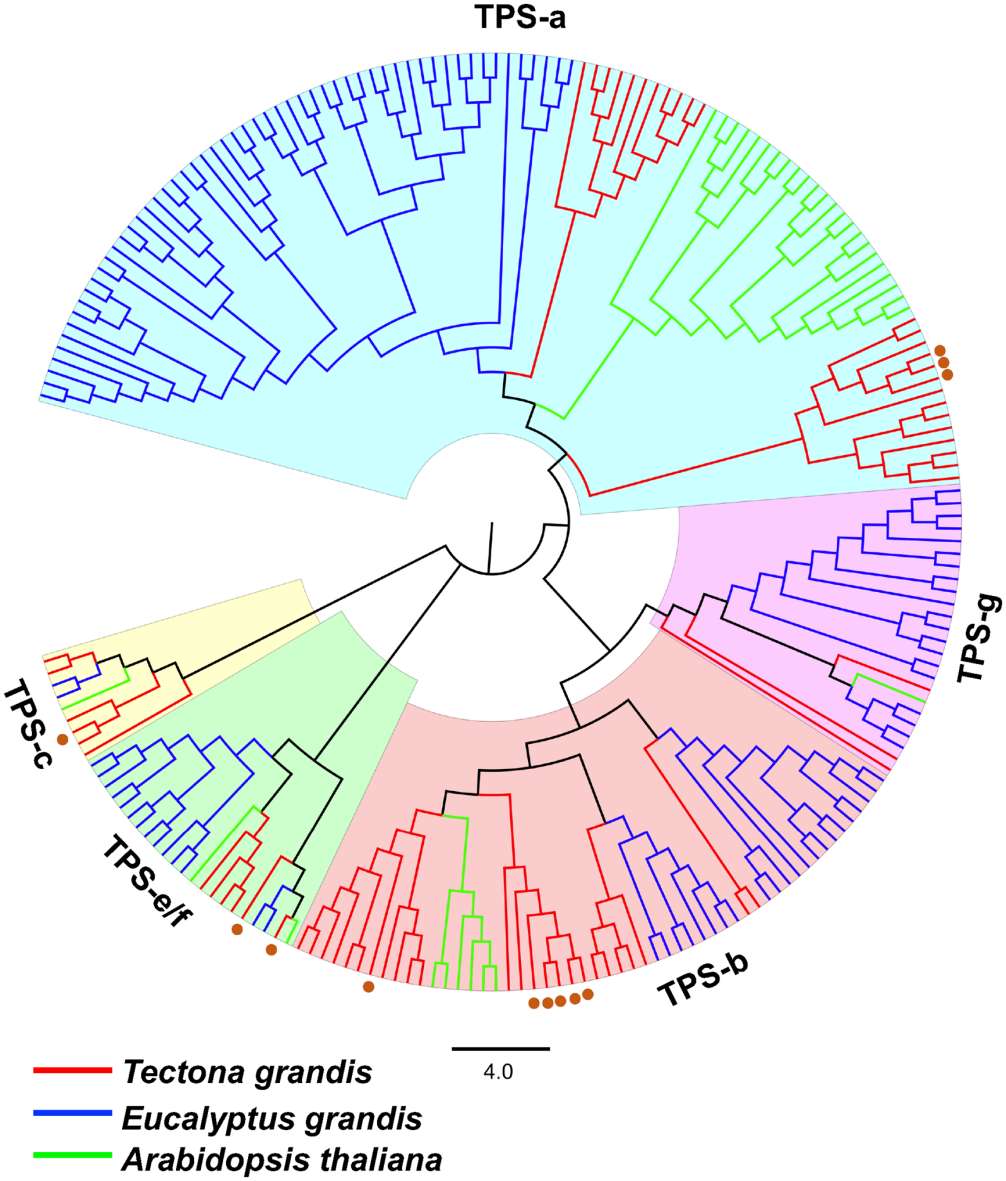
Maximum likelihood tree of peptide sequences of terpene synthase (TPS) family genes from the *Tectona grandis* (red branches), *Arabidopsis thaliana* (green branches), and *Eucalyptus grandis* (blue branches). Red dots denote teak TPSs expressed in stems.

**Figure 5:**
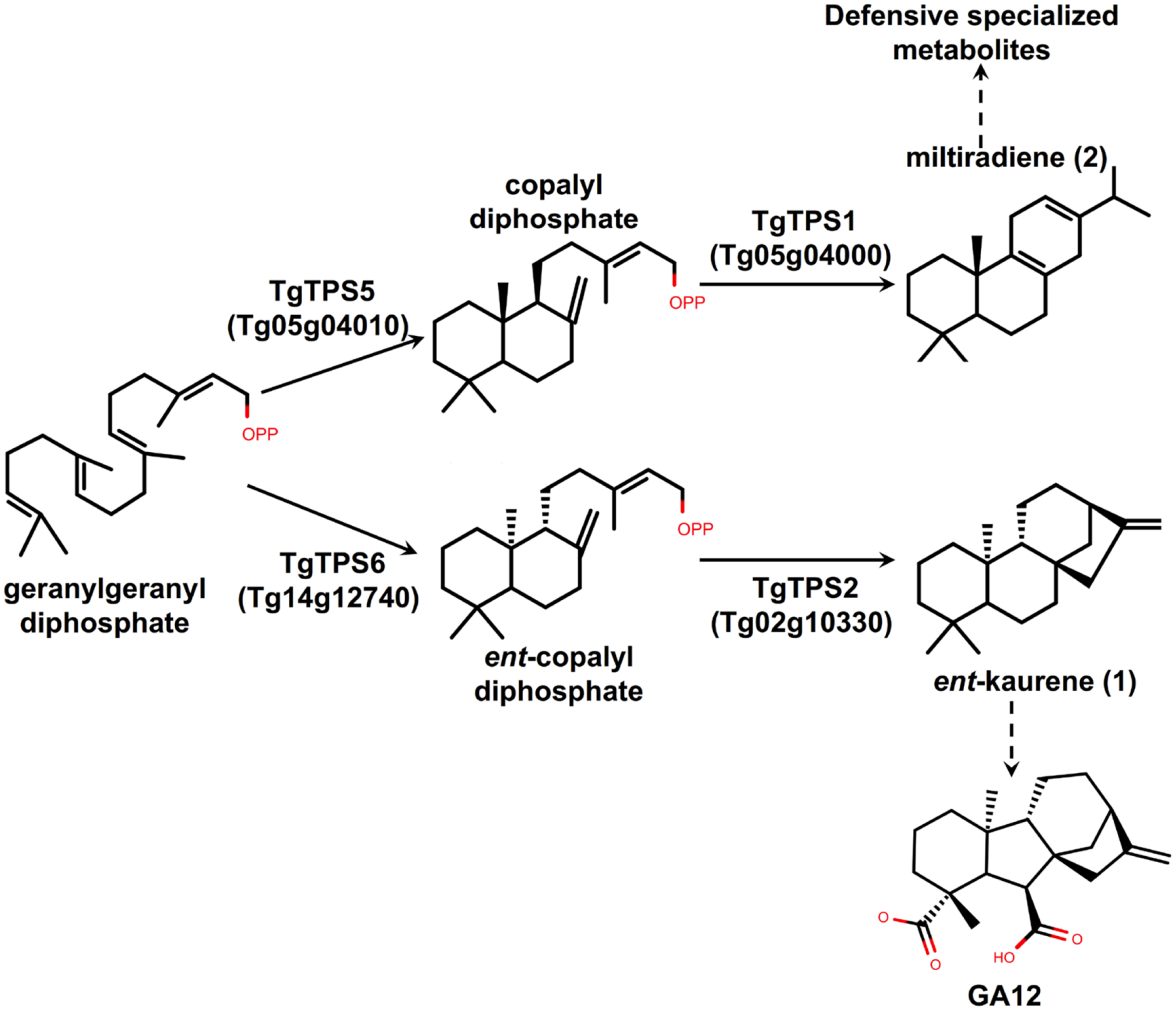
Proposed diterpene pathway based on functional validation.

Terpenes are a large class of specialized metabolites involved in plant defense and pollinator attraction [35]. Terpene synthases (TPSs) are key genes involved in terpenoid biosynthesis and are often found in physical clusters in the genome [36]. A sequence similarity search using BLASTP (v2.2.31+ with default parameters) [23] was performed using the teak peptide models against a set of reference TPS peptides (Supplementary Table S5). After filtering out teak peptides shorter than 350 amino acids or having less than 30% identity to the most similar reference sequence, 65 candidate TPSs were identified, of which, 41 TPSs were located in 14 tandem clusters (Supplementary Table S6). Phylogenetic analysis of teak TPSs and those from *A. thaliana* and *Eucalyptus grandis* W. Hill ex Maiden indicate that multiple recent species-specific tandem duplication events contributed to an expansion in TPS number in teak, consistent with previous findings [37] (Fig. 4; Supplementary Information). Twelve teak TPSs were expressed in stems; seven of these are tandemly duplicated, suggesting these recent tandemly duplicated genes may retain similar functions (Supplementary Table S6). To validate our TPS annotation, four teak diterpene synthases were amplified from leaf tissues and tested for functional verification through transient expression in *Nicotiana benthamiana* Domin (Supplementary Information). The results demonstrated that TgTPS6 (Tg14g12740) catalyzed the formation of *ent*-copalyl diphosphate, while TgTPS2 (Tg02g10330) converted that product to *ent*-kaurene in the first committed steps of gibberellic acid hormone biosynthesis (Fig. 5; Supplementary Fig. S2). TgTPS5 (Tg05g04010) and TgTPS1 (Tg05g04000) are located adjacent to each other on the genome and form the pathway to miltiradiene (Fig. 5), an intermediate in the biosynthesis of defense-related specialized metabolites found in many members of Lamiaceae.

### Transcriptomic analysis of TPSs and cytochrome P450 enzymes

**Figure 6:**
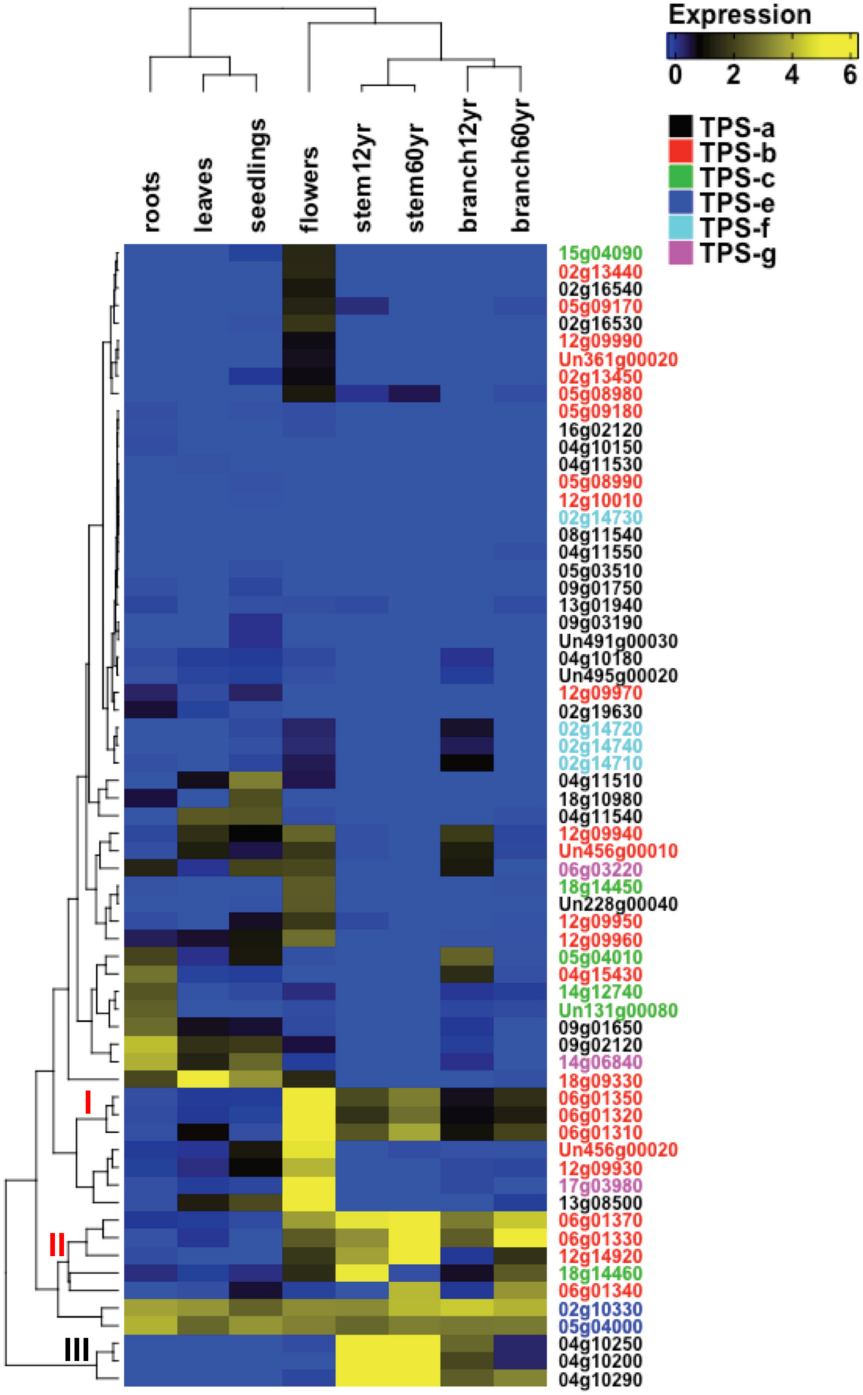
Expression of terpene synthases in various tissues of teak. Six monoterpene synthases (clade I and II as denoted on the nodes) and three putative sesquiterpene synthases (clade III) exhibited high expression in branches and stems of 12- and 60-year-old teak trees.

**Figure 7:**
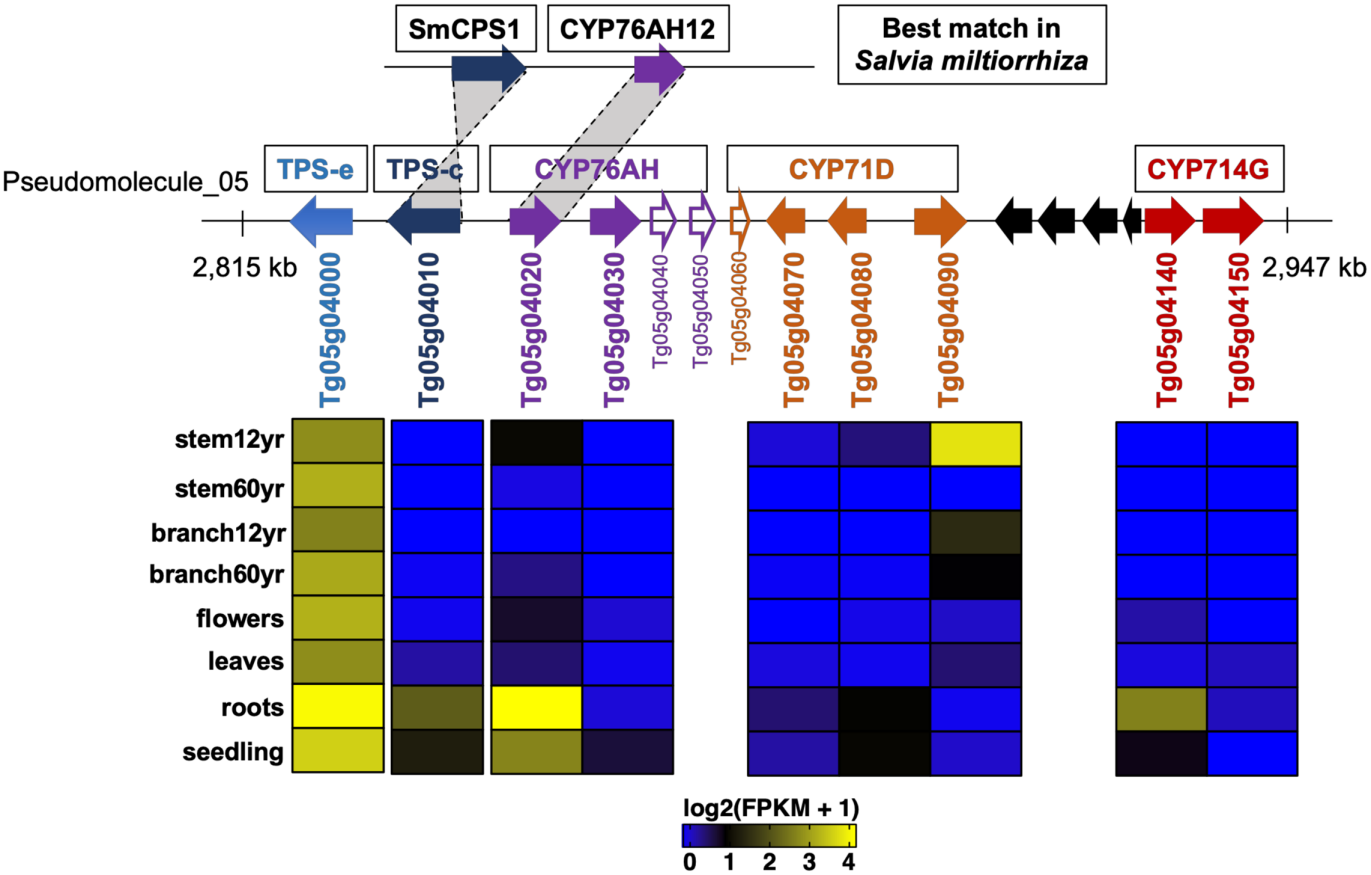
A physical cluster of TPS/CYP genes on pseudomolecule 5 and their expression in different tissues of teak. Horizontal arrows denote genes with their gene classification listed above and gene IDs below, where unfilled arrows denote partial genes and black arrows denote genes that are not TPS/CYP.

Transcriptomic analysis of diverse tissues of teak, including leaves, flowers, roots, seedling, and branch and stem secondary xylem of different ages, revealed seven putative monoterpene synthases from subfamily TPS-b (Fig. 6, clades I and II) and three putative sesquiterpene synthases from subfamily TPS-a (Fig. 6, clade III) that were highly expressed in woody tissues, including 12- and 60-year-old branches and stems (Fig. 6). These TPSs are likely responsible for the synthesis of defense-related compounds, including unknown, specialized metabolites that contribute to the termite resistance and defense of wood tissues from other pests and pathogens in teak [38]. Most specialized metabolites, including terpenes, require cytochrome P450 enzymes (CYPs) that modify the terpene scaffold; similar to TPSs, CYPs are often found in physical clusters in the genome [10]. Through sequence similarity searches, 377 CYP genes were identified, of which, 248 (66%) occurred in physical clusters (Supplementary Table S5). In addition, many TPSs and CYPs were clustered together, i.e., of 65 TPSs and 377 CYPs, 20 TPSs and 31 CYPs were co-located in 12 physical clusters. For example, a cluster on pseudomolecule 5 consisted of two TPSs (TPS-e, TPS-c) and eight complete and two partial CYP genes (i.e., four copies of CYP76AH, four copies of CYP71D, and two copies of CYP714G). Similar to the pattern observed for lignin pathway genes, neofunctionalization of expression across tissues was observed for the CYP subfamily genes (Fig. 7). It is notable that a putative TPS-e (Tg05g04000) was constitutively expressed in all tissues examined, and a putative TPS-c (Tg05g04010) was co-regulated with a putative CYP76AH31 (Tg05g04020) (Fig. 7). From a biochemical perspective, subfamily CYP76AH contains several P450s that are involved in (di)terpene specialized metabolism and occur in close physical proximity in other species [36, 39]. In another species of Lamiaceae, *Salvia miltiorrhiza* Bunge, the best match for the teak TPS-c/CYP76AH31 cluster was the SmCPS1/CYP76AH12 gene cluster (Fig. 7), which is involved in the biosynthesis of tanshinone diterpenes and organized in several gene clusters, suggesting physical clustering is a major mechanism regulating expression of genes involved in the same biosynthetic pathway in plants [40].

## Conclusion

In summary, we generated a chromosomal-scale assembly of the teak genome that, when coupled with high-quality functional annotation, will facilitate the discovery of candidate genes related to traits critical for sustainable production of teak and for anti-insecticidal natural products. Furthermore, the high contiguity of our improved assembly will permit comparative genomics studies and exploration of physical gene clustering, facilitating discovery of key biosynthetic pathways.

## Availability of supporting data

All sequences generated in this study, including PacBio long reads and Illumina short reads, were deposited in the NCBI SRA under BioProject PRJNA493753. The genome assembly, annotation files, expression matrix, and other supporting data can be accessed at the *GigaScience* database GigaDB [41] and via Dryad [42].

## Additional files

**Table S1**. BUSCO results. This is available as a separate XLS file.

**Table S2**. Mapping of RNA-seq reads to the assembly. This is available as a separate XLS file.

**Table S3**. Genes involved in the core phenylpropanoid biosynthetic pathway and their expression abundance (FPKM: fragments per kilobase of exon model per million reads mapped) in *Tectona grandis*. This is available as a separate XLS file.

**Table S4**. Gene expression correlations between tissues and biological replicates (NCBI SRA SRP059970) This is available as a separate XLS file.

**Table S5**. Terpene synthases (TPSs) used as references for identification of teak TPSs. This is available as a separate XLS file.

**Table S6**. Tandem clusters of candidate terpene synthases and CYPs and their expression abundance (FPKM: fragments per kilobase of exon model per million reads mapped) in *Tectona grandis*. This is available as a separate XLS file.

**Figure S1**. Inference of ancient WGDs in *Tectona grandis*. (A) Histogram (*K*_S_ plot) showing the age distribution of putative paralogous gene pairs overlaid with mixture models of inferred WGD events. The mixture model with an inferred peak at *K*_S_ = 0.60 (red) was corroborated by SiZer analysis (Chaudhuri and Marron, 1999), while modeled peaks at *K*_S_ = 0.22, 1.36 (blue) were not. (B) SiZer map displaying significant features in the observed *K*_S_ distribution at varying bandwidths. As indicated in the key, colors signify either a significant increase (blue), significant decrease (red), or no significant change (purple) in the data distribution.

**Figure S2**. Activities of diterpene synthases after transient expression in *Nicotiana benthamiana*. On the left are total ion chromatograms of hexane extracts from plant leaves. On the right are mass spectra from individual peaks. Controls express CfDXS and CfGGPPS, but no recombinant TPS. Hexane extract from the moss *Physcomitrella patens* was used as a standard for *ent*-kaurene. *Zea mays* ZmAN2 (Genbank: AY562491) is a known *ent*-copalyl diphosphate synthase. *Coleus forskohlii* CfTPS1 (Genbank: KF444506), and CfTPS3 (Genbank: KF444508) are known (+)-copalyl diphosphate and miltiradiene synthases, respectively.

giga-d-18-00458_original_submission.pdf

giga-d-18-00458_revision_1.pdf

response_to_reviewer_comments_original_submission.pdf

reviewer_1_report_original_submission -- Nicolas Delhomme, Ph. D. rer. nat. 12/10/2018 Reviewed

reviewer_2_report_original_submission -- Meg Staton 12/11/2018 Reviewed

Supplemental Files

## Abbreviations

BLAST: Basic Local Alignment Search Tool; BUSCO: Benchmarking Universal Single-Copy Orthologs; CCR: cinnamyl CoA reductase; CRL: custom repeat library; CYP: cytochrome P450 enzyme; FPKM: fragments per kilobase of exon model per million reads mapped; HCT: shikimate O-hydroxycinnamoytransferase; NCBI: National Center for Biotechnology Information; PacBio: Pacific Biosciences; RNA-seq: RNA-sequencing; SMRT sequencing: single molecule real time sequencing; SRA: Sequenmce Read Archive; TPS: terpene synthase; WGD: whole-genome duplication.

## Competing interests

The authors declare that they have no competing interests.

## Funding

Funds for this study were provided by a grant to C.R.B., N.D., D.S., and P.S. from the National Science Foundation Plant Genome Research Program (IOS-1444499), a grant to C.R.B. and B. H. from the Michigan State University Strategic Partnership Grants Program, and from Hatch funds to C.R.B.

## Author contributions

C.R.B., B.H., and D.Z. designed the experiment. D.Z. and J.P.H. conducted genome assembly and annotation. D.Z. generated the expression matrix and physical clustering of TPSs/CYPs. W.W.B. and S.R.J. conducted the TPS phylogeny and functional verification of four TPSs. G.G. and T.K. conducted whole-genome duplication analysis. B.B. analyzed TPS expression. C.R.B., B.H., P.S., D.S., and N.D. provided intellectual insights and supervised the work. All authors read and wrote part of the manuscript.
