# Supplementary material for: A chromosomal-scale genome assembly of Tectona grandis reveals the importance of tandem gene duplication and enables discovery of genes in natural product biosynthetic pathways
Source: Gigascience. 2019 Jan 30;8(3):giz005. doi: 10.1093/gigascience/giz005 (PMC6394206; doi:10.1093/gigascience/giz005)
Supplement: giga-d-18-00458_original_submission.pdf [file giz005_giga-d-18-00458_original_submission.pdf]

GigaScience

A chromosomal-scale genome assembly of Tectona grandis enables discovery of natural product biosynthetic pathway genes key to development of sustainable teak production

--Manuscript Draft--

**Manuscript Number:**	GIGA-D-18-00458
**Full Title:**	A chromosomal-scale genome assembly of Tectona grandis enables discovery of natural product biosynthetic pathway genes key to development of sustainable teak production
**Article Type:**	Data Note
**Funding Information:**	US NSF (IOS-1444499) — Prof. C Robin Buell<br>Hatch funds (NA) — Prof. C Robin Buell
**Abstract:**	Background<br><br>Teak, a member of the Lamiaceae family, produces one of the most expensive hardwoods in the world. High demand coupled with deforestation have caused a decrease in natural teak forests, and future supplies will be reliant on teak plantations. Hence, selection of teak tree varieties for clonal propagation with superior growth performance is of great importance, and access to high-quality genetic and genomic resources can accelerate the selection process by identifying genes underlying desired traits.<br><br>Findings<br><br>To facilitate teak research and variety improvement, we generated a highly contiguous, chromosomal-scale genome assembly using high-coverage PacBio long reads coupled with high-throughput chromatin conformation capture (Hi-C). Of the 18 teak chromosomes, we generated 17 near-complete pseudomolecules with one chromosome present as two chromosome arm scaffolds. Genome annotation yielded 31,168 genes encoding 46,826 gene models, of which, 39,930 and 41,155 had Pfam domains and expression evidence, respectively. We identified 14 clusters of tandem-duplicated terpene synthases (TPSs), genes central to the biosynthesis of terpenes which are involved in plant defense and pollinator attraction. Transcriptome analysis revealed 10 TPSs highly expressed in woody tissues, of which, 8 were in tandem, revealing the importance of resolving tandemly duplicated genes and the quality of the assembly and annotation. We also validated the enzymatic activity of four TPSs to demonstrate the function of key TPSs.<br><br>Conclusions<br><br>In summary, this high-quality chromosomal-scale assembly and functional annotation of the teak genome will facilitate the discovery of candidate genes related to traits critical for sustainable production of teak and for anti-insecticidal natural products.
**Corresponding Author:**	C Robin Buell<br>Michigan State University<br>East Lansing, Michigan UNITED STATES
**Corresponding Author Secondary Information:**	
**Corresponding Author's Institution:**	Michigan State University
**Corresponding Author's Secondary Institution:**	
**First Author:**	Dongyan Zhao
**First Author Secondary Information:**	

Powered by Editorial Manager® and ProduXion Manager® from Aries Systems Corporation

Order of Authors:	Dongyan Zhao
	John P. Hamilton
	Wajid Waheed Bhat
	Sean R. Johnson
	Grant T. Godden
	Taliesin J. Kinser
	Benoît Boachon
	Natalia Dudareva
	Douglas E. Soltis
	Pamela S. Soltis
	Bjoern Hamberger
	C Robin Buell
Order of Authors Secondary Information:	
Additional Information:	
Question	Response
Are you submitting this manuscript to a special series or article collection?	No
**Experimental design and statistics**<br><br>Full details of the experimental design and statistical methods used should be given in the Methods section, as detailed in our Minimum Standards Reporting Checklist. Information essential to interpreting the data presented should be made available in the figure legends.<br><br>Have you included all the information requested in your manuscript?	Yes
**Resources**<br><br>A description of all resources used, including antibodies, cell lines, animals and software tools, with enough information to allow them to be uniquely identified, should be included in the Methods section. Authors are strongly encouraged to cite Research Resource Identifiers (RRIDs) for antibodies, model organisms and tools, where possible.	Yes

Have you included the information requested as detailed in our Minimum Standards Reporting Checklist?	
**Availability of data and materials**<br><br>All datasets and code on which the conclusions of the paper rely must be either included in your submission or deposited in publicly available repositories (where available and ethically appropriate), referencing such data using a unique identifier in the references and in the “Availability of Data and Materials” section of your manuscript.<br><br>Have you have met the above requirement as detailed in our Minimum Standards Reporting Checklist?	Yes

manuscript

Click here to download Manuscript Zhao-Teak-manuscript-gigaSci_RB_DZ.docx

A chromosomal-scale genome assembly of *Tectona grandis* enables discovery of natural product biosynthetic pathway genes key to development of sustainable teak production

Dongyan Zhao[1], John P. Hamilton[1], Wajid Waheed Bhat[2,3], Sean R. Johnson[2], Grant T. Godden[4], Taliesin J. Kinser[4,5], Benoît Boachon[6], Natalia Dudareva[6], Douglas E. Soltis[4,5], Pamela S. Soltis[4], Bjoern Hamberger[2], C. Robin Buell[1,7,8,*]

[1]Department of Plant Biology, Michigan State University, East Lansing, MI 48824, USA

[2]Department of Biochemistry and Molecular Biology, Michigan State University, East Lansing, MI 48824, USA

[3]Department of Pharmacology and Toxicology, Michigan State University, East Lansing, MI 48824, USA

[4]Florida Museum of Natural History, University of Florida, Gainesville, FL 32611, USA

[5]Department of Biology, University of Florida, Gainesville, FL 32611, USA

[6]Department of Biochemistry, Purdue University, West Lafayette, IN 47907, USA

[7]Plant Resilience Institute, Michigan State University, East Lansing, MI 48872, USA

[8]MSU AgBioResearch, Michigan State University, East Lansing, MI 48872, USA

Email addresses: Dongyan Zhao <zhaodon4@msu.edu>, John P. Hamilton <jham@msu.edu>, Wajid Waheed Bhat <bhatwaji@msu.edu>, Sean R. Johnson <seanRjohnson@gmail.com>, Grant T. Godden <g0ddengr@ufl.edu>, Taliesin J. Kinser <tkinser@ufl.edu>, Benoît Boachon <benoit.boachon@gmail.com>, Natalia Dudareva <dudareva@purdue.edu>, Douglas E. Soltis <dsoltis@ufl.edu>, Pamela S. Soltis <psoltis@flmnh.ufl.edu>, Bjoern Hamberger <hamberge@msu.edu>, C. Robin Buell <buell@msu.edu>

*Correspondence should be addressed to: C. Robin Buell, buell@msu.edu

Manuscript type: Data note

Note: Reviewers can access the genome sequence and annotation using the following temporary URL: https://datadryad.org/review?doi=doi:10.5061/dryad.77b2422

Abstract

Background: Teak, a member of the Lamiaceae family, produces one of the most expensive hardwoods in the world. High demand coupled with deforestation have caused a decrease in natural teak forests, and future supplies will be reliant on teak plantations. Hence, selection of teak tree varieties for clonal propagation with superior growth performance is of great importance, and access to high-quality genetic and genomic resources can accelerate the selection process by identifying genes underlying desired traits.

Findings: To facilitate teak research and variety improvement, we generated a highly contiguous, chromosomal-scale genome assembly using high-coverage PacBio long reads coupled with high-throughput chromatin conformation capture (Hi-C). Of the 18 teak chromosomes, we generated 17 near-complete pseudomolecules with one chromosome present as two chromosome arm scaffolds. Genome annotation yielded 31,168 genes encoding 46,826 gene models, of which, 39,930 and 41,155 had Pfam domains and expression evidence, respectively. We identified 14 clusters of tandem-duplicated terpene synthases (TPSs), genes central to the biosynthesis of terpenes which are involved in plant defense and pollinator attraction. Transcriptome analysis revealed 10 TPSs highly expressed in woody tissues, of which, 8 were in tandem, revealing the importance of resolving tandemly duplicated genes and the quality of the assembly and annotation. We also validated the enzymatic activity of four TPSs to demonstrate the function of key TPSs.

Conclusions: In summary, this high-quality chromosomal-scale assembly and functional annotation of the teak genome will facilitate the discovery of candidate genes related to traits critical for sustainable production of teak and for anti-insecticidal natural products.

Keywords: Teak, chromosomal-scale assembly, terpene synthases, tandem-duplicated genes,

Data Description

Introduction

Teak (*Tectona grandis* L.f.; 2*n* = 2*x* = 36), a member of the angiosperm family Lamiaceae, produces timber of high value due to its durability, hardness, appearance, and resistance to biotic and abiotic stresses. Teak is one of the most expensive hardwoods in the world, with an average price for high-quality logs ranging from $600-1000/m^3 USD [1]. High demand coupled with deforestation have caused a decrease in natural teak forests, and future supplies will be reliant on teak plantations. Hence, selection of teak tree varieties for clonal propagation with superior growth performance is of great importance, and access to high-quality genetic and genomic resources can accelerate the selection process by identifying genes underlying desired traits. The only available genome assembly for teak (hereafter referred to as the "released assembly") was completed using short-reads and low-coverage (7x) nanopore long reads [2]; while improved compared to other short-read assembled plant genomes, the released assembly is still highly fragmented with an N50 scaffold length of 358 kbp.

DNA extraction and genome sequencing

Teak seeds were obtained from Sheffield's Seed Company [3]. High molecular weight DNA was extracted from young leaves of a 2-week-old plant grown in the greenhouse using a modified CTAB method [4]. Long read sequencing was done using Pacific Biosciences RSII and Sequel single-molecule sequencers at the University of Delaware Sequencing & Genotyping Center. Briefly, SMRTbell DNA libraries were constructed from genomic DNA using SMRTbell Template Prep Kit 1.0-SPv3 as per the manufacturer's instructions (Pacific Biosciences, Menlo Park, CA). The library was size selected using the BluePippin Size-selection system and protocol for 15 Kbp size selection (Sage Science, Amherst, MA). Following size selection, the average library fragment size was 25 kb based on the Fragment Analyzer sizing profile (Advanced Analytical Technologies, Arkeny, IA). The library was sequenced for 6 hours on 10 SMRT cells using P6-C4 chemistry on the PacBio RS II instrument (Pacific Biosciences, Menlo Park, CA) and 10 hours on 4 SMRT cells using 2.0 sequencing chemistry on the PacBio Sequel instrument (Pacific Biosciences, Menlo Park, CA). A total of ~4.7 million PacBio long reads were generated, which is an estimated ~104x coverage of the estimated 325 Mbp teak genome. Additionally, whole genome short-read sequencing libraries were generated using Illumina

TruSeq Nano DNA Library Preparation Kit (Cat. No. FC-121-4001) and sequenced to 150-nt paired end reads on Illumina HiSeq 4000.

Genome assembly and quality assessment

The raw reads were error-corrected (canu -correct) and trimmed (canu -trim) for low-quality bases and reads ≥ 1 kb were used to generate the initial assembly (canu -assemble) with a correctedErrorRate of 0.09% [5]. The assembly consists of 1,474 contigs with a total length of 338 Mbp, 20 Mbp larger than the released assembly (Table 1). The initial assembly was polished using the raw PacBio reads using Arrow [6], followed by three rounds of error correction with 643.7 million Illumina short reads (570x coverage, Table 2) using Pilon [7]. A Dovetail Hi-C library was prepared as described previously [8]. The initial PacBio assembly, shotgun reads, and Dovetail Hi-C library reads were used as input data for scaffolding using HiRise [9]. Shotgun and Dovetail Hi-C library sequences were aligned to the initial assembly using a modified SNAP read mapper [10]. The separation of aligned Dovetail Hi-C read pairs were analyzed by HiRise to produce a likelihood model for genomic distance between read pairs, and the model was used to identify and break putative mis-joins, to score prospective joins, and make joins above a threshold. The Hi-C scaffolding resulted in 936 scaffolds (referred to as "improved assembly", hereafter), with an N50 scaffold size of 18.5 Mbp, which is a 46x improvement of genome contiguity over the released assembly (Table 3). The 19 largest scaffolds (minimum length of 8.6 Mbp) represented 90% of the assembled 338 Mbp genome; of the 18 teak chromosomes, we generated 17 near-complete pseudomolecules with one chromosome present as two chromosome arm scaffolds (Figure 1). The completeness of our improved assembly was also demonstrated by the presence of tandem tracts of the telomere repeat sequence in nine of the 19 pseudomolecules; two pseudomolecules contained telomere tracks at both ends (Figure 1). A tandem array of 5S rRNA sequence (135 copies with each at 496 bp) was found in pseudomolecule 10 spanning >67.5 kbp, highlighting the power of long reads in resolving highly repetitive sequences. Around 98% of the whole genome shotgun reads aligned to the improved assembly, of which, 94 - 98% of the reads were properly paired (Table 2). The representation of genic sequences in our improved assembly was confirmed by detection of 94.4% of the Benchmarking Universal Single-Copy Orthologs (BUSCO [11]; C:92.3%[S:82.4%,D:9.9%],F:2.1%,M:5.6%, n:1440; Supplementary Table S1) and by alignment of 89% - 93% of transcriptome reads from

publicly available RNA-seq datasets derived from diverse tissues of other teak accessions (Supplementary Table S2).

Genome annotation

The genome was annotated as described previously [12]. A custom repeat library (CRL) was generated for teak by running RepeatModeler [13], excluding protein-coding genes from the repeat library, and adding the Viridiplantae RepBase repeats. Repeatmasking revealed that 32.02% of the improved assembly was identified as repetitive sequence, 3-fold more compared to that reported in the released assembly (11%). The improved assembly was masked using the CRL. RNA-seq alignments were used to train the *ab initio* gene finder, Augustus [14], and gene models were predicted on the hard-masked assembly. The predicted gene models were refined by running PASA2 [15], followed by manual curation, yielding 31,168 genes encoding 46,826 gene models, of which, 39,930 and 41,155 had Pfam domains and expression evidence, respectively.

Detection of whole genome duplication events

Whole genome duplications (WGD) can contribute to genetic innovations underlying chemical defense against co-evolving insect herbivores, as exemplified by evidence from studies of other plant groups (e.g., Brassicales [16]). To infer WGD events in teak, we used the DupPipe pipeline [17] to analyze coding sequences representing the longest isoforms of genes (Supplemental Information). Gaussian mixture models predicted three components within the observed *Ks* distribution of teak, with mean values at K_S = 0.22, 0.60, 1.36 (Supplementary Fig. S1A). Of these, a peak at K_S = 0.60 was corroborated as a significant feature by a SiZer analysis (Supplementary Fig. S1B), providing evidence for at least one WGD event in teak. Whether or not this WGD event is lineage-specific or shared by other Lamiaceae is a subject of active research.

The phenylpropanoid pathway genes and their expression

Teak is known for strong wood, and we were able to identify all of the genes involved in the phenylpropanoid pathway which leads to lignin formation (Supplementary Table S3). We identified physical clusters of genes in lignin biosynthetic pathway based on if: 1) there were no more than 10 genes in between on a single pseudomolecule and 2) the pairwise gene distance was less than 100 kbp. Notably, four of the 11 core genes in the phenylpropanoid pathway were

present in tandem copies, with shikimate O-hydroxycinnamoytransferase (HCT) having three tandem clusters of two copies each and one cluster of five copies (Fig. 2). For 20 of the 45 genes in the phenylpropanoid pathway, clear neofunctionalization at the expression level was observed for F5H, COMT, PAL, and HCT. Interestingly, cinnamyl CoA reductase (CCR), which catalyzes the first committed step of the lignin-specific branch, was in a physical cluster with five copies of HCT; within this physical cluster, only one of the five HCT genes (Tg16g10070) and CCR (Tg16g10210) were constitutively expressed in all tissues (Fig. 2).

Identification of terpene synthases (TPSs) and functional verification

Terpenes are a large class of specialized metabolites involved in plant defense and pollinator attraction [18]. Terpene synthases (TPSs) are key genes involved in terpenoid biosynthesis and are often found in physical clusters in the genome [19]. Through sequence similarity searches, 65 TPSs were identified, of which, 41 TPSs were located in 14 tandem clusters (Supplementary Table S4). Phylogenetic analysis of teak TPSs and those from *Arabidopsis thaliana* L. Heynh. and *Eucalyptus grandis* W. Hill ex Maiden indicate that multiple recent species-specific tandem duplication events contributed to an expansion in TPS number in teak, consistent with previous findings [20] (Fig. 3; Supplementary Information). Twelve teak TPSs were expressed in stem; seven of these are tandemly duplicated, suggesting these recent tandemly duplicated genes may retain similar functions (Supplementary Table S4). To validate our TPS annotation, four teak diterpene synthases (diTPSs) were amplified from leaf tissues and tested for functional verification through transient expression in *Nicotiana benthamiana* Domin (Supplemental Information). The results demonstrated that TgTPS6 (Tg14g12740) catalyzed the formation of *ent*-copalyl diphosphate, while TgTPS2 (Tg02g10330) converted that product to *ent*-kaurene in the first committed steps of gibberellic acid hormone biosynthesis (Fig. 4; Supplementary Fig. S2). TgTPS5 (Tg05g04010) and TgTPS1 (Tg05g04000) are located adjacent to each other on the genome and form the pathway to miltiradiene (Fig. 4), an intermediate in the biosynthesis of defense-related specialized metabolites found in many members of Lamiaceae.

Transcriptomic analysis of TPSs and cytochrome P450 enzymes

Transcriptomic analysis of diverse tissues of teak, including leaves, flowers, roots, seedling, and branch and stem secondary xylem of different ages, revealed seven putative monoterpene synthases from subfamily TPS-b (Fig. 5, clades I and II) and three putative sesquiterpene

synthases from subfamily TPS-a (Fig. 5, clade III) that were highly expressed in woody tissues, including 12- and 60-year-old branches and stems (Fig. 5). These TPSs are likely responsible for the synthesis of defense-related compounds, including unknown, specialized metabolites that contribute to the termite resistance and defense of wood tissues from other pests and pathogens in teak [21].

Most specialized metabolites, including terpenes, require cytochrome P450 enzymes (CYPs) that modify the terpene scaffold; similar to TPSs, CYPs are often found in physical clusters in the genome [10]. Through sequence similarity searches, 377 CYP genes were identified, of which, 248 (66%) occurred in physical clusters (Supplementary Table S4). In addition, many TPSs and CYPs were clustered together, i.e., of 65 TPSs and 377 CYPs, 20 TPSs and 31 CYPs were co-located in 12 physical clusters. For example, a cluster on pseudomolecule 5 consisted of two TPSs (TPS-e, TPS-c) and eight complete and two partial CYP genes (i.e., four copies of CYP76AH, four copies of CYP71D, and two copies of CYP714G). Similar to the pattern observed for lignin pathway genes, neofunctionalization of expression across tissues was observed for the CYP subfamily genes (Fig. 6). It is notable that a putative TPS-e (Tg05g04000) was constitutively expressed in all tissues examined and a putative TPS-c (Tg05g04010) was co-regulated with a putative CYP76AH31 (Tg05g04020) (Fig. 6). From a biochemical perspective, subfamily CYP76AH contains several P450s that are involved in (di)terpene specialized metabolism and occur in close physical proximity in other species [19,22]. In another species of Lamiaceae, *Salvia miltiorrhiza* Bunge, the best match for the teak TPS-c/CYP76AH31 cluster was the SmCPS1/CYP76AH12 gene cluster (Fig. 6), which is involved in the biosynthesis of tanshinone diterpenes and organized in several gene clusters, suggesting physical clustering is a major mechanism regulating expression of genes involved in the same biosynthetic pathway in plants [23].

Conclusion

In summary, we generated a chromosomal-scale assembly of the teak genome that, when coupled with high-quality functional annotation, will facilitate the discovery of candidate genes related to traits critical for sustainable production of teak and for anti-insecticidal natural products. Furthermore, the high contiguity of our improved assembly will permit comparative

genomics studies and exploration of physical gene clustering, facilitating discovery of key biosynthetic pathways.

Availability of supporting data

All sequences generated in this study, including PacBio long reads and Illumina short reads, were deposited in the NCBI SRA under BioProject PRJNA493753. The genome assembly, annotation files, and expression matrix can be accessed at Dryad (Provisional DOI: doi:10.5061/dryad.77b2422). For review purposes, these data can be viewed through this anonymous URL (https://datadryad.org/review?doi=doi:10.5061/dryad.77b2422).

Abbreviations

Cetyl trimethylammonium bromide (CTAB), single molecule real time sequencing (SMRT sequencing), custom repeat library (CRL), terpene synthase (TPS), di-terpene synthase (di-TPS), Whole genome duplications (WGD), RNA-sequencing (RNA-seq), cytochrome P450 enzymes (CYPs)

Competing interests

The authors have declared that no competing interests exists.

Funding

Funds for this study were provided by a grant to CRB, ND, DS, and PS from the National Science Foundation Plant Genome Research Program (IOS-1444499) and from Hatch funds to CRB.

Author contributions

C.R.B, B.H., and D.Z. designed the experiment, D.Z. and J.P.H. conducted genome assembly and annotation, D.Z. generated expression matrix and physical clustering of TPSs/CYPs, W.W.B. and S.R.J. conducted the TPS phylogeny and functional verification of 4 TPSs, G.G. and T.K. conducted whole-genome duplication analysis, B.B. analyzed TPS expression, C.R.B., B.H., P.S., D.S., and N.D. provided intellectual insights and supervised the work. All authors read and wrote part of the manuscript.

Acknowledgements

We thank Dr. David Nelson (The University of Tennessee, Health Science Center) for annotating the new P450s. We thank Krystle Wiegert-Rininger and Joshua Wood for taking care of the teak plants. We thank Brieanne Vaillancourt for handling the data storage and submission and providing valuable comments on the manuscript.

References

1 Food and Agriculture Organization of the United Nations. Global teak trade in the aftermath of Myanmar's log export ban. 2015.

2 Yasodha R, Vasudeva R, Balakrishnan S, et al. Draft genome of a high value tropical timber tree, Teak (Tectona grandis L. f): insights into SSR diversity, phylogeny and conservation. DNA Res 2018;25:409–419.

3 Sheffield's Seed Company. Available at https://sheffields.com/ Accessed March 7, 2017.

4 Doyle JJ. Isolation of plant DNA from fresh tissue. Focus. Focus (Madison) 1990;12:13–15.

5 Koren S, Walenz BP, Berlin K, et al. Canu: scalable and accurate long-read assembly via adaptive k-mer weighting and repeat separation. Genome Res 2017;27:722–736.

6 Pacfici Biosciences. SMRT tools. 2017

7 Walker BJ, Abeel T, Shea T, et al. Pilon: An Integrated Tool for Comprehensive Microbial Variant Detection and Genome Assembly Improvement. PLoS One 2014;9:e112963.

8 Lieberman-Aiden E, van Berkum NL, Williams L, et al. Comprehensive mapping of long-range interactions reveals folding principles of the human genome. Science 2009;326:289–293.

9 Putnam NH, O'Connell BL, Stites JC, et al. Chromosome-scale shotgun assembly using an in vitro method for long-range linkage. Genome Res 2016;26:342–350.

10 The UC Berkeley AMP Lab. Scalable Nucleotide Alignment Program. Available at http://snap.cs.berkeley.edu.

11 Simão FA, Waterhouse RM, Ioannidis P, et al. BUSCO: assessing genome assembly and annotation completeness with single-copy orthologs. Bioinformatics 2015;31:3210–3212.

12 Zhao D, Hamilton JP, Pham GM, et al. De novo genome assembly of *Camptotheca acuminata*, a natural source of the anti-cancer compound camptothecin. Gigascience 2017;6:1–7.

13 Smit A, Hubley R. *RepeatModeler Open-1.0.* 2008.

14 Stanke M, Waack S. Gene prediction with a hidden Markov model and a new intron submodel. Bioinformatics 2003;19:ii215-ii225.

15 Campbell MA, Haas BJ, Hamilton JP, et al. Comprehensive analysis of alternative splicing in rice and comparative analyses with Arabidopsis. BMC Genomics 2006;7:327.

16 Edger PP, Heidel-Fischer HM, Bekaert M, et al. The butterfly plant arms-race escalated by gene and genome duplications. Proc Natl Acad Sci 2015;112:8362–8366.

17 Barker MS, Dlugosch KM, Dinh L, et al. EvoPipes.net: Bioinformatic Tools for Ecological and Evolutionary Genomics. Evol Bioinforma 2010;6:EBO.S5861.

18 Dudareva N, Klempien A, Muhlemann JK, et al. Biosynthesis, function and metabolic engineering of plant volatile organic compounds. New Phytol 2013;198:16–32.

19 Boutanaev AM, Moses T, Zi J, et al. Investigation of terpene diversification across multiple sequenced plant genomes. Proc Natl Acad Sci U S A 2015;112:E81-8.

20 Mint Evolutionary Genomics Consortium. Phylogenomic Mining of the Mints Reveals Multiple Mechanisms Contributing to the Evolution of Chemical Diversity in Lamiaceae. Mol Plant 2018;11:1084–1096.

21 Pandey V, Singh AK, Sharma RP. Biodiversity of Insect Pests associated with Teak (Tectona grandis L.f.) in Eastern Uttar Pradesh of India. Res J For 2010;4:136–144.

22 Zi J, Matsuba Y, Hong YJ, et al. Biosynthesis of lycosantalonol, a cis-prenyl derived diterpenoid. J Am Chem Soc 2014;136:16951–16953.

23 Xu H, Song J, Luo H, et al. Analysis of the Genome Sequence of the Medicinal Plant Salvia miltiorrhiza. Mol Plant 2016;9:949–952.

Figure legends

Figure 1. Gene and repeat density across the 19 pseudomolecules in the assembly. Green dots denote telomere tracks.

Figure 2. Differential expression of tandem copies of genes in lignin biosynthetic pathway. stem12yr: stem secondary xylem of a 12-year-old teak tree; stem60yr: stem secondary xylem of a 60-year-old teak tree; branch12yr: branch secondary xylem of a 12-year-old teak tree; branch60yr: branch secondary xylem of a 60-year-old teak tree.

Figure 3. Maximum likelihood tree of peptide sequences of terpene synthase (TPS) family genes from the *Tectona grandis* (red branches), *Arabidopsis thaliana* (green branches), and *Eucalyptus grandis* (blue branches). Red dots denote teak TPSs expressed in stems.

Figure 4. Proposed diterpene pathway based on the functional verification.

Figure 5. Expression of terpene synthases (TPSs) in various tissues of teak. Six monoterpene synthases (clade a & b) and three putative sesquiterpene synthases (clade c) exhibited high expression in branches and stems of 12- and 60-year-old teak trees.

Figure 6. A physical cluster of TPS/CYP genes on pseudomolecule 5 and their expression in different tissues of teak. Horizontal arrows denote genes with their gene classification listed above and gene IDs below, where unfilled arrows denote partial genes and black arrows denote genes that are not TPS/CYP.

Tables

Table 1. Metrics of contigs assembled using PacBio reads.

Metrics	Initial assembly (bp)
Total contigs	1,474
Total length	338,318,549
Maximum contig size	21,267,566
Minimum contig size	1,168
N50 contig size	3,749,470
N90 contig size	52,675
Average contig size	229,524

Contig size	Total size (bp)	%Total assembly	# Contigs
≥1 Mbp	248,187,558	73.37	64
0.5 - 1 Mbp	267,412,682	79.06	91
0.1 - 0.5 Mbp	291,028,790	86.04	198
0.05 - 0.1 Mbp	305,851,391	90.42	420

Table 2. Whole genome shot-gun reads

Sample name	NCBI SRA Run ID	QC-passed reads	mapped	properly paired out of total reads
teak_TruSeq_01	SRR7984127	168,566,966	165,783,328 (98.35%)	163,390,358 (97.40%)
teak_TruSeq_02	SRR7984127	188,504,116	185,541,771 (98.43%)	182,934,854 (97.15%)
TEC_AA_01	SRR7984129	371,978,214	364,473,434 (97.98%)	357,722,188 (96.65%)
TEC_AA_02	SRR7984129	394,477,964	386,545,305 (97.99%)	379,620,884 (96.72%)
TEC_AB_01	SRR7984130	89,116,777	87,087,277 (97.72%)	84,001,838 (94.93%)
TEC_AB_02	SRR7984130	81,436,054	79,540,000 (97.67%)	76,733,986 (94.89%)

Table 3. Metrics of the assembled scaffolds.

	Current assembly
Total scaffolds	936
Assembly size (bp)	338,300,341
Maximum scaffold length (bp)	20,661,910
Minimum scaffold length (bp)	1,168
N50 scaffold size (bp)	16,483,567
Average scaffold size (bp)	361,432

Size cutoff	Size (bp)	% Assembly size	# Scaffolds
Scaffolds ≥ 1 Mb	304,435,280	89.99	19
Scaffolds ≥ 100 kb	308,724,809	91.26	41
Scaffolds ≥ 50 kb	314,467,503	92.96	134
Scaffolds ≥ 10 kb	338,276,936	99.99	931

Additional files

Supplementary tables

Table S1. BUSCO results.

This is available as a separate XLS file.

Table S2. Mapping of RNA-seq reads to the assembly.

This is available as a separate XLS file.

Table S3. Genes involved in the core phenylpropanoid biosynthetic pathway and their expression in teak.

This is available as a separate XLS file.

Table S4. Tandem clusters of candidate terpene synthases (TPSs) and cytochrome P450 enzymes (CYPs) in teak.

This is available as a separate XLS file.

Supplementary figures

Figure S1. Inference of ancient WGDs in *Tectona grandis*. (A) Histogram (K_S plot) showing the age distribution of putative paralogous gene pairs overlaid with mixture models of inferred WGD events. The mixture model with an inferred peak at K_S = 0.60 (red) was corroborated by SiZer analysis (Chaudhuri and Marron, 1999), while modeled peaks at K_S = 0.22, 1.36 (blue) were not. (B) SiZer map displaying significant features in the observed K_S distribution at varying bandwidths. As indicated in the key, colors signify either a significant increase (blue), significant decrease (red), or no significant change (purple) in the data distribution.

Figure S2. Activities of diterpene synthases after transient expression in *Nicotiana benthamiana*. On the left are total ion chromatograms of hexane extracts from plant leaves. On the right are mass spectra from individual peaks. Controls express CfDXS and CfGGPPS, but no recombinant TPS. Hexane extract from the moss *Physcomitrella patens* was used as a standard for *ent*-kaurene. *Zea mays* ZmAN2 (Genbank: AY562491) is a known *ent*-copalyl diphosphate synthase.

Coleus forskohlii CfTPS1 (Genbank: KF444506), and CfTPS3 (Genbank: KF444508) are known (+)-copalyl diphosphate and miltiradiene synthases, respectively.

Figure 1

Click here to download Figure Figure 1.pdf

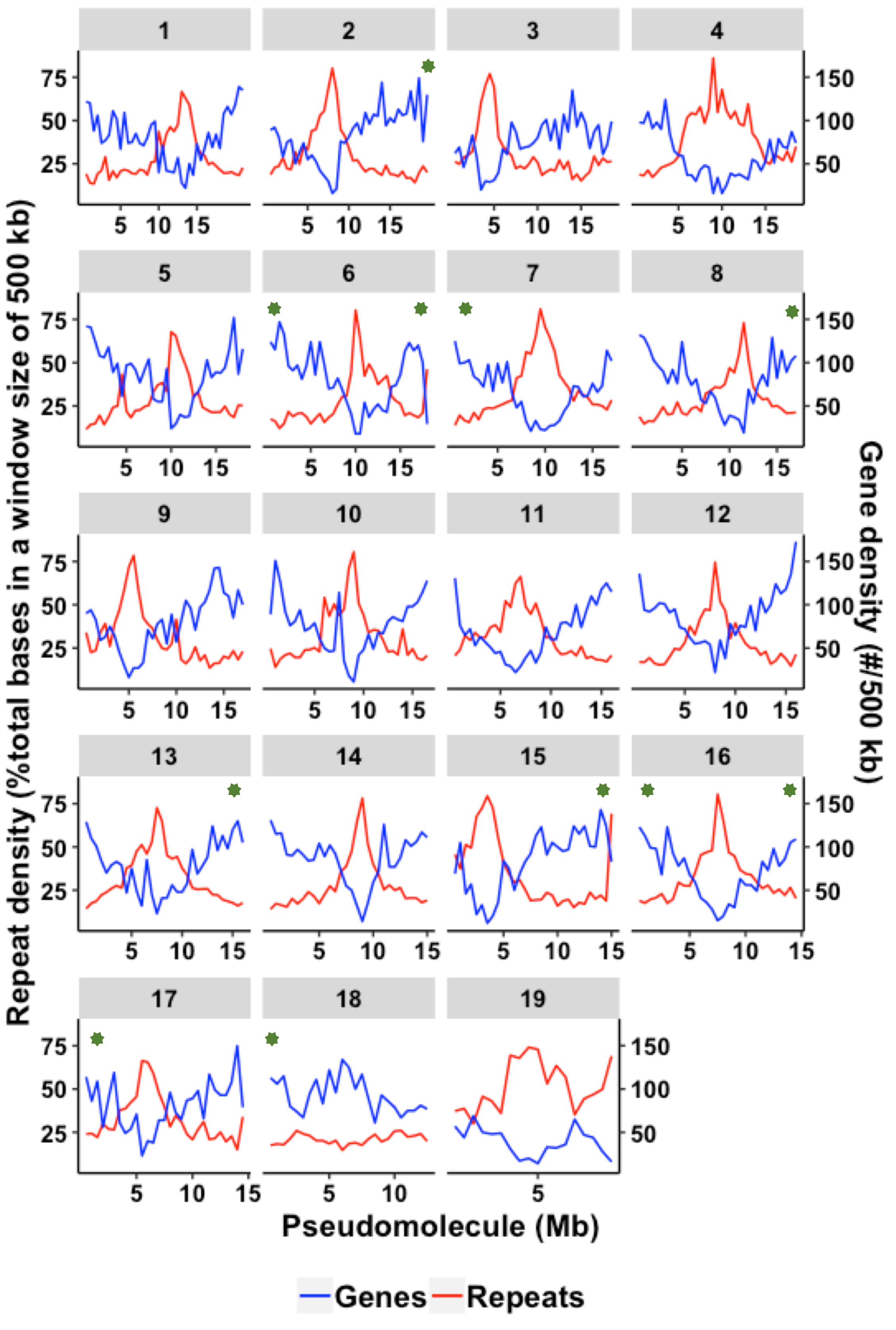

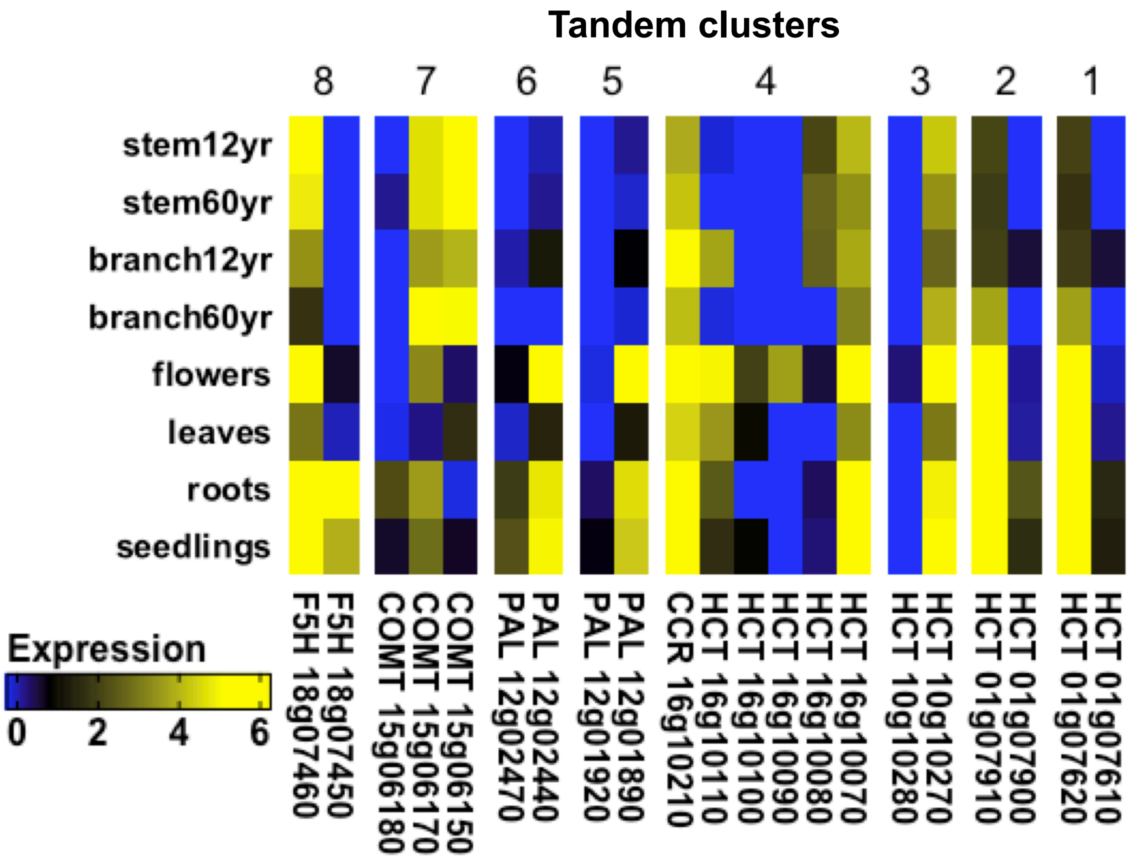
Tandem clusters
8
7
6
5
4
3
2
1
stem12yr
stem60yr
branch12yr
branch60yr
flowers
leaves
roots
seedlings
Expression
0 2 4 6
F5H 18g07460
F5H 18g07450
COMT 15g06180
COMT 15g06170
COMT 15g06150
PAL 12g02470
PAL 12g02440
PAL 12g01920
PAL 12g01890
CCR 16g10210
HCT 16g10110
HCT 16g10100
HCT 16g10090
HCT 16g10080
HCT 16g10070
HCT 10g10280
HCT 10g10270
HCT 01g07910
HCT 01g07900
HCT 01g07620
HCT 01g07610

Click here to download Figure Figure 3.pdf

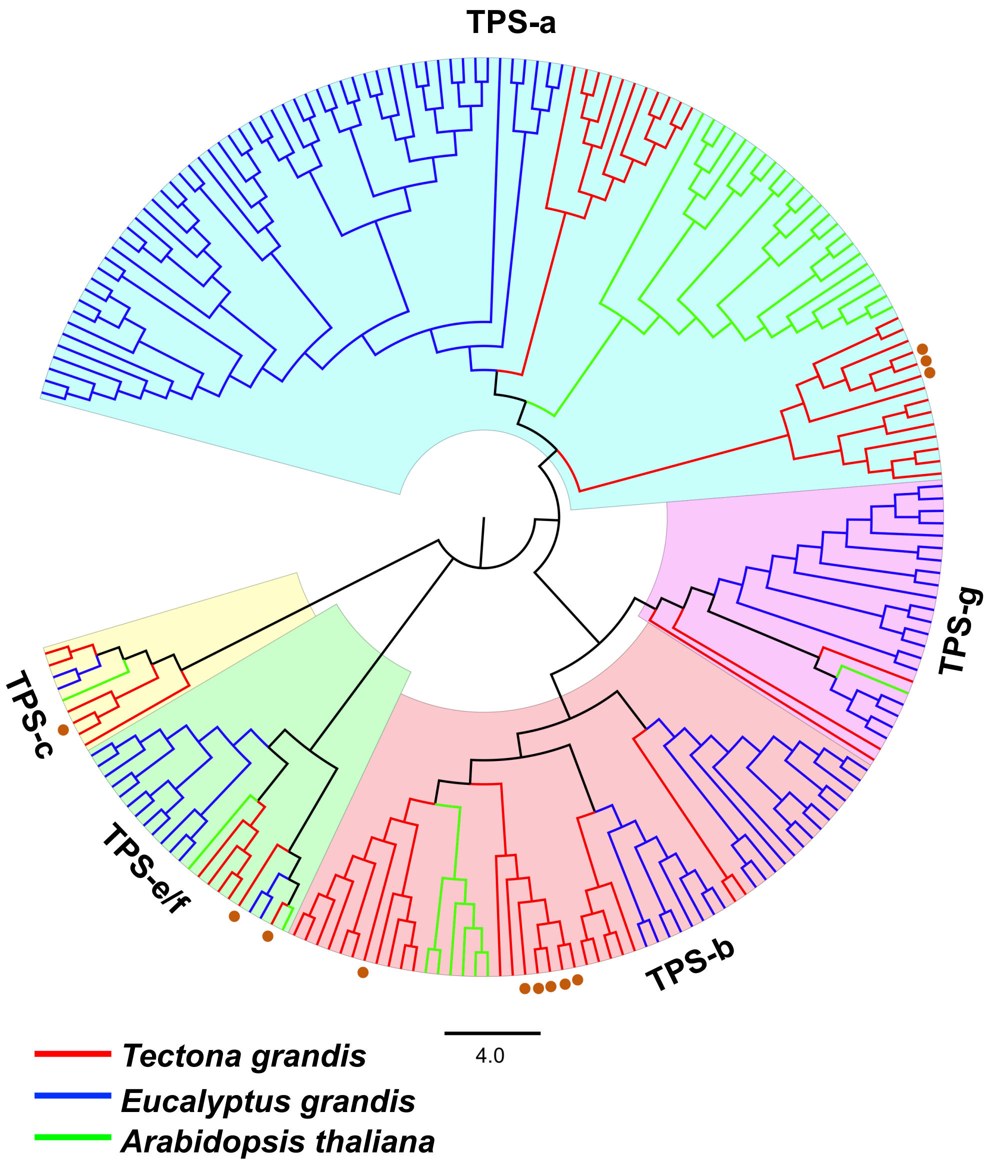

Figure 4

Click here to download Figure Figure 4.pdf

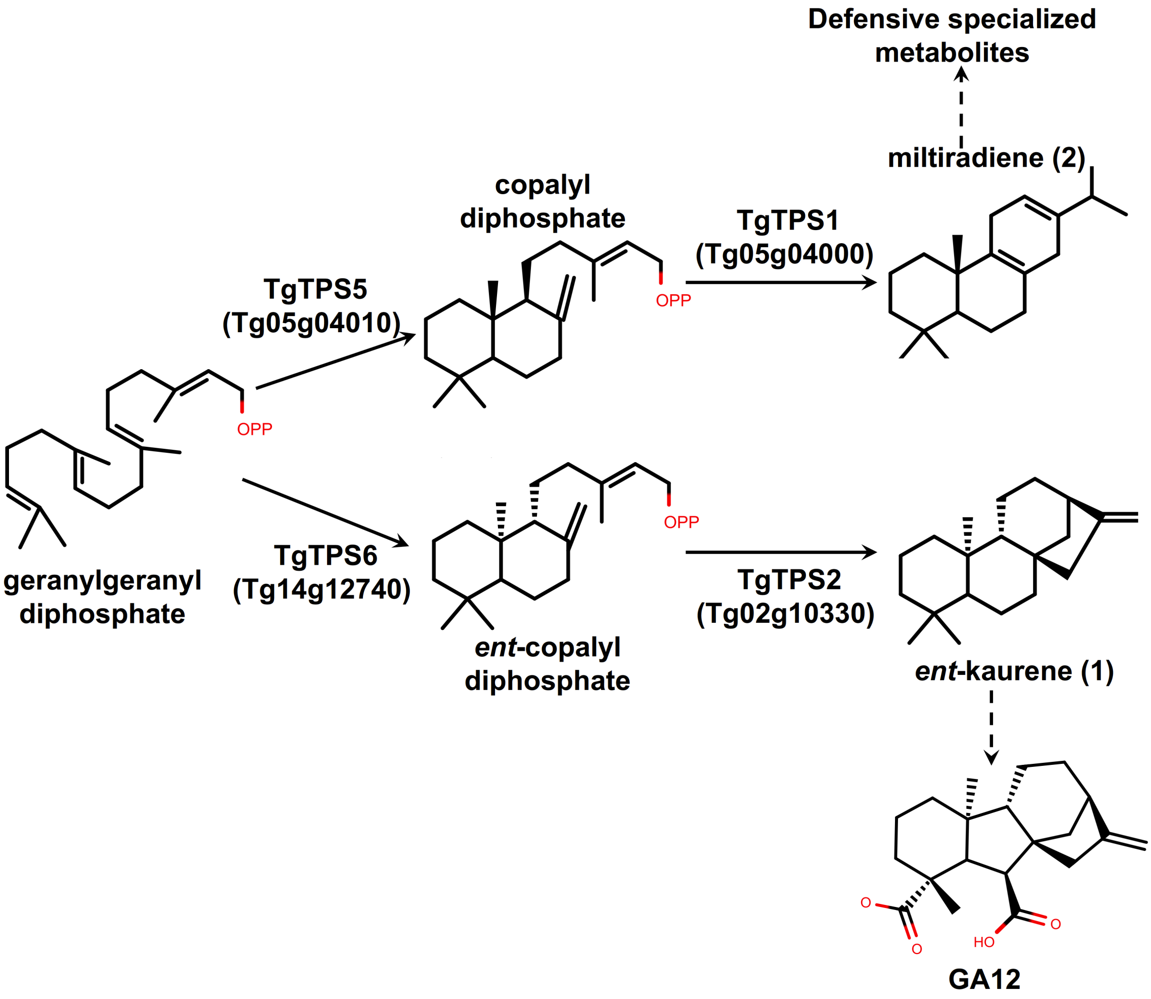

Figure 5

Click here to download Figure Figure 5.pdf

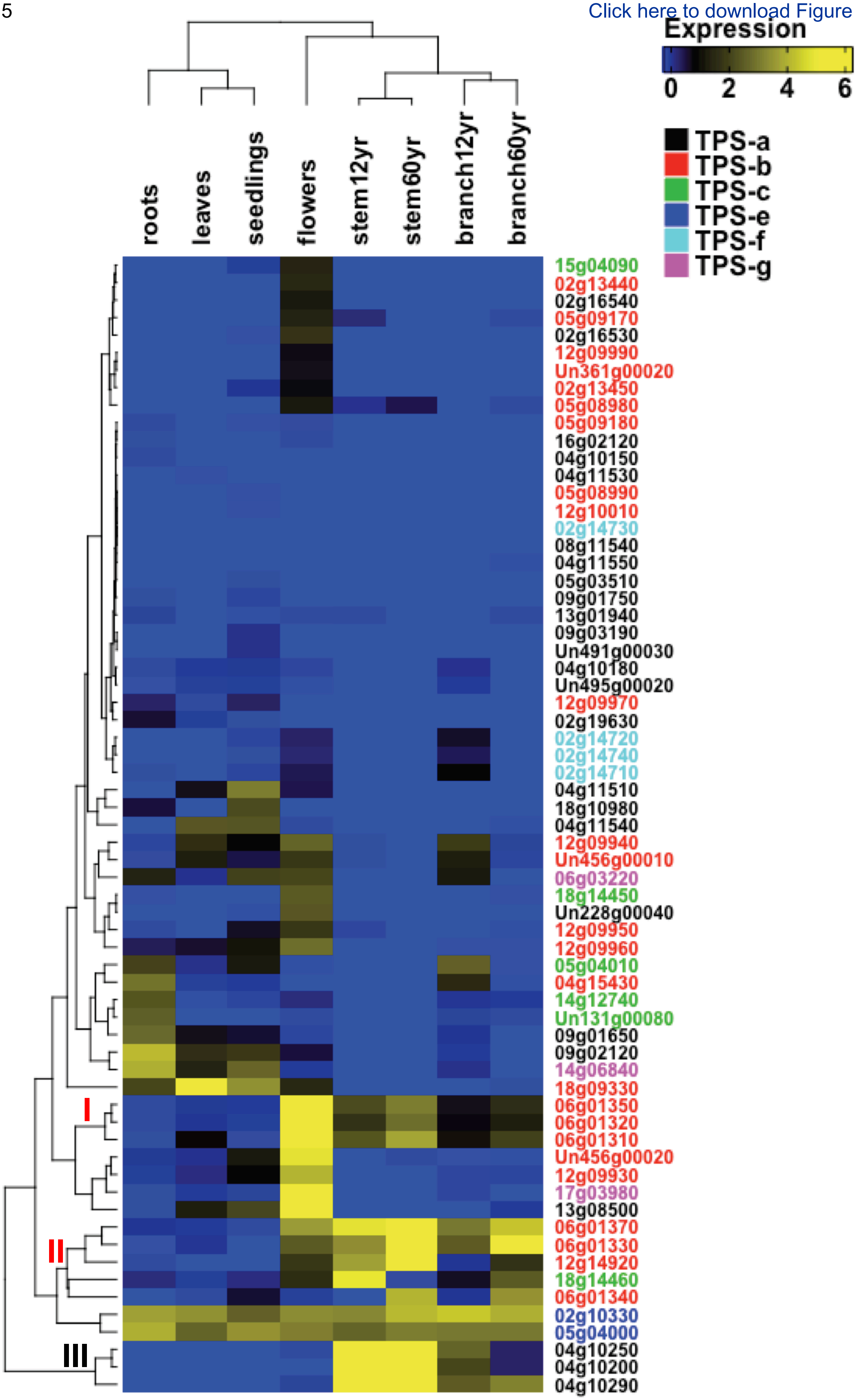

Figure 6

Click here to download Figure Figure 6.pdf

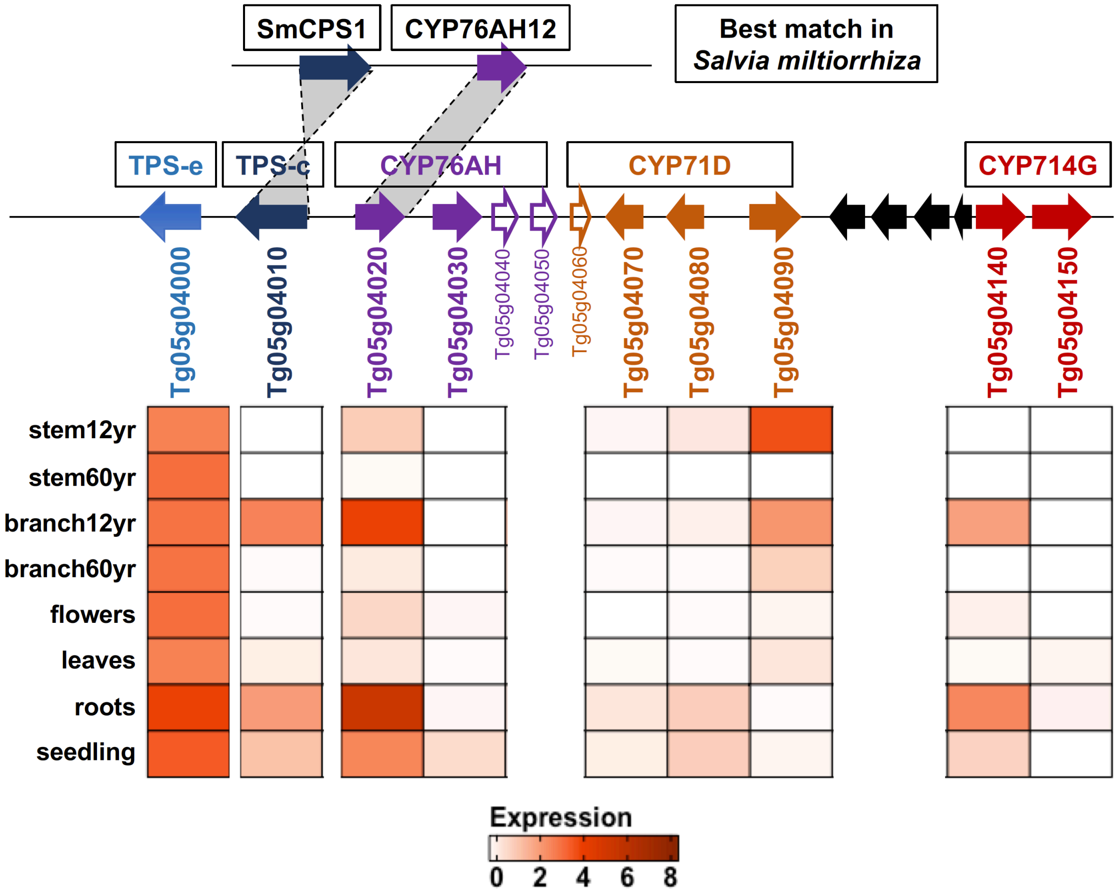

Supplementary Table S1

Click here to access/download
Supplementary Material
Table S1.xlsx

Supplementary Table S2

Click here to access/download
Supplementary Material
Table S2.xlsx

Supplementary Table S3

Click here to access/download
Supplementary Material
Table S3.xlsx

Supplementary Table S4

Click here to access/download
Supplementary Material
Table S4.xlsx

Supplementary Methods and Figures

Click here to access/download
Supplementary Material
Zhao-Teak-sup_RB_DZ.docx
