# Supplementary material for: A chromosomal-scale genome assembly of Tectona grandis reveals the importance of tandem gene duplication and enables discovery of genes in natural product biosynthetic pathways
Source: Gigascience. 2019 Jan 30;8(3):giz005. doi: 10.1093/gigascience/giz005 (PMC6394206; doi:10.1093/gigascience/giz005)
Supplement: giga-d-18-00458_revision_1.pdf [file giz005_giga-d-18-00458_revision_1.pdf]

GigaScience

A chromosomal-scale genome assembly of Tectona grandis reveals the importance of tandem gene duplication and enables discovery of genes in natural product biosynthetic pathways

--Manuscript Draft--

**Manuscript Number:**	GIGA-D-18-00458R1
**Full Title:**	A chromosomal-scale genome assembly of Tectona grandis reveals the importance of tandem gene duplication and enables discovery of genes in natural product biosynthetic pathways
**Article Type:**	Data Note
**Funding Information:**	US NSF (IOS-1444499) — Prof. C Robin Buell<br>Hatch funds (NA) — Prof. C Robin Buell
**Abstract:**	Background: Teak, a member of the Lamiaceae family, produces one of the most expensive hardwoods in the world. High demand coupled with deforestation have caused a decrease in natural teak forests, and future supplies will be reliant on teak plantations. Hence, selection of teak tree varieties for clonal propagation with superior growth performance is of great importance, and access to high-quality genetic and genomic resources can accelerate the selection process by identifying genes underlying desired traits.<br><br>Findings: To facilitate teak research and variety improvement, we generated a highly contiguous, chromosomal-scale genome assembly using high-coverage PacBio long reads coupled with high-throughput chromatin conformation capture. Of the 18 teak chromosomes, we generated 17 near-complete pseudomolecules with one chromosome present as two chromosome arm scaffolds. Genome annotation yielded 31,168 genes encoding 46,826 gene models, of which, 39,930 and 41,155 had Pfam domain and expression evidence, respectively. We identified 14 clusters of tandem-duplicated terpene synthases (TPSs), genes central to the biosynthesis of terpenes which are involved in plant defense and pollinator attraction. Transcriptome analysis revealed 10 TPSs highly expressed in woody tissues, of which, 8 were in tandem, revealing the importance of resolving tandemly duplicated genes and the quality of the assembly and annotation. We also validated the enzymatic activity of four TPSs to demonstrate the function of key TPSs.<br><br>Conclusions: In summary, this high-quality chromosomal-scale assembly and functional annotation of the teak genome will facilitate the discovery of candidate genes related to traits critical for sustainable production of teak and for anti-insecticidal natural products.
**Corresponding Author:**	C Robin Buell<br>Michigan State University<br>East Lansing, Michigan UNITED STATES
**Corresponding Author Secondary Information:**	
**Corresponding Author's Institution:**	Michigan State University
**Corresponding Author's Secondary Institution:**	
**First Author:**	Dongyan Zhao
**First Author Secondary Information:**	
**Order of Authors:**	Dongyan Zhao
	John P. Hamilton
	Wajid Waheed Bhat

Powered by Editorial Manager® and ProduXion Manager® from Aries Systems Corporation

	Sean R. Johnson
	Grant T. Godden
	Taliesin J. Kinser
	Benoît Boachon
	Natalia Dudareva
	Douglas E. Soltis
	Pamela S. Soltis
	Bjoern Hamberger
	C Robin Buell
**Order of Authors Secondary Information:**	

Response to Reviewers:

Dear Editor and Reviewers,

Thank you very much for your time and effort to review this manuscript. We appreciate all the constructive comments and suggestions and have provided a point-by-point response below (in blue).

Both reviewers mention that more in-depth information regarding the methods is needed. Publishing articles with highly reproducible methods and data is one of our main goals at GigaScience, please take care to fully address the reviewers' comments in a revised manuscript.

Author response: We provided detailed information on methods as requested by you and reviewers. We uploaded a marked up version of the manuscript that highlightstexts revisions made in our manuscript.

On a minor note, our genome Data Notes usually show a photo of an example of the sequenced species as "Figure 1", please consider to include this as well (as part of the article, the photo will be published under our creative commons licence, please make sure you have the rights to include it under these terms).

Author response: We provided a photo of a young teak tree as Figure 1 and made changes to other figures accordingly.

Reviewer reports:

Reviewer #1: In their manuscript, Dongyan Zhao et al., present the genome assembly of Tectona grandis realised using the most recent sequencing technologies, followed by the identification and validation of genes important to wood formation, a trait of interest in teak. The manuscript is well written and the analyses appear to have been robustly conducted, but their lack of details prevents me for being more convinced. This is my only significant comment to the manuscript as it stands, it is otherwise very well written and should be a good resource for the community. As a note, I do find that the title is too boldly written considering the content presented and that the readership would gain from a more fitting title (i.e. the pathways discussed in the manuscript are well known and there is not proof as of yet that their update knowledge in teak will lead to a more sustainable teak production).

Author response: Thank you for the constructive comments. We modified the title to “A chromosomal-scale genome assembly of Tectona grandis reveals the importance of tandem gene duplication and enables discovery of genes in natural product biosynthetic pathway”.

Major comment
1) The description of the transcriptome analysis is completely missing in the main text, as are the details of which datasets were retrieved. Overall, I would wish for the supplementary document to contain the details of all analyses, including the software used, their versions and any non-default parameters - as was done for the WGD

analysis. The supplementary information available from the FTP hints that more comprehensive analyses were done that is reported in the main text (e.g. classification of the gene models in different confidence bins, etc.). Such details should be made more readily visible as they would improve the manuscript' impact.

Author response: We have provided more details in the main text as suggested including adding all the versions of the software. Please see the point-to-point response in the details below.

Minor comments
1) p. 3 l.38 The number of scaffolds of the released assembly could also be given. As a stand alone figure, an N50 value is not particularly informative.

Author response: The total number of scaffolds and maximum scaffold length have been added to the main text as shown below; additional information is in Tables 1 and 2.

"The only available genome assembly for teak (hereafter referred to as the "released assembly") was completed using short-reads and low-coverage (7x) nanopore long reads [2]; while improved compared to other short-read assembled plant genomes, the released assembly is still highly fragmented, comprising 2,993 scaffolds with the maximum and N50 scaffold length of 1.7 Mb and 358 kbp, respectively."

2) p.4 l. 83 Detail the BUSCO categories (write them in full)

Author response: We have edited the as suggested.
"The representation of genic sequences in our improved assembly was confirmed by detection of 94.4% of the Benchmarking Universal Single-Copy Orthologs (BUSCO v2.0 [11]; Complete:92.3%[Single-copy:82.4%,Duplicated:9.9%], Fragmented:2.1%, Missing:5.6%, Total BUSCO groups searched:1440; Supplementary Table S1)"

3) p.5. l.88 Briefly describe the annotation process. Further in the same paragraph detail which evidences were used for Augustus (maybe discuss why Maker-P was not used) and which datasets and parameters were used for PASA2. Also provide any custom scripts in a public repository that were used for the manual curation of the genes and gene models.

Author response: The annotation process is now thoroughly described in the text.

"A custom repeat library (CRL) was generated for teak by running RepeatModeler (v1.0.8) [13], excluding protein-coding genes using ProtExcluder (v1.1) [14], and adding the Viridiplantae RepBase repeats [15]. The improved assembly was masked with the CRL using RepeatMasker (v4.0.6) with default parameters [16], which revealed that 32.02% of the improved assembly was identified as repetitive sequence, 3-fold more compared to that reported in the released assembly (11%). To generate transcript evidence for genome annotation, raw RNA-seq reads from a previous study were downloaded from NCBI (SRA SRP059970) and adapters and low-quality bases were removed using Cutadapt (v1.8.1) [17] requiring a minimum base quality of 20 and minimum size of 20-nt. The processed reads were aligned to the improved assembly using TopHat2 (v2.0.13) [18] with default parameters. Genome-guided transcript assemblies for each aligned RNA-seq library were created using Trinity (v2.2.0) [19] using the default parameters. Gene models were predicted using Augustus (v3.1) [20] by first training Augustus with the leaf RNA-seq alignments, then generating gene predictions on the hard-masked genome. The predicted gene models were refined by running PASA2 (v2.1.0) [21] using the genome-guided transcript assemblies and two rounds of annotation comparison. Genes of interest (e.g., terpene synthases as described below) were manually curated using Apollo (v1.11.8) [22]. The final working set of annotations was comprised of 31,168 loci and 46,826 gene models. Functional annotation was assigned using BLAST [23] searches against the Arabidopsis thaliana (L.) Heynh annotation (TAIR10) [24] and Swiss-Prot plant proteins (downloaded on Nov. 17, 2016), and a search against Pfam (v31) [25] using HMMER (v3.1b2) [26] with a cutoff of 1e-5. A high confidence subset of the working gene model set was identified by identifying models with an FPKM (fragments per kilobase of exon model per million

reads mapped, a normalized estimation of gene expression abundance) > 0 in any of the RNA-Seq libraries or a match in Pfam (v31). The high confidence gene model set is comprised of 41,155 gene models and 39,930 loci. "

4) p5. l. 104 provide more details about what a SiZer analysis is. In general, extend the methods to contain the parameters used by the different tools, when non default (e.g. those for DupPipe line 100).

Author response: We have edited the text as suggested; see below.
"These components were further compared with results from a SiZer analysis [29] (implemented with the 'multimode' R statistical package [30]), which distinguishes true data features from noise by testing for significant increases or decreases, or no significant changes across an observed KS distribution at various bandwidths (Supplemental Information)."

"To infer WGD events in teak, we used the DupPipe pipeline with default settings [28] to analyze coding sequences representing the longest isoforms of genes (Supplemental Information)."

5) Detail how the phenylpropanoid pathway genes were identified. Similarly, detail how this was achieved for the TPSs, including tools, versions, non default parameters.

Author response: We have edited the text as suggested; see below.

"Using phenylpropanoid pathway genes in A. thaliana [31] as bait, the corresponding candidate genes in teak were identified based on orthology analysis between teak and A. thaliana using OrthoFinder v2.0 with default parameters [32]."

"A sequence similarity search using BLASTP (v2.2.31+ with default parameters) [23] was performed using the teak peptide models against a set of reference TPS peptides (Supplementary Table S5). After filtering out teak peptides shorter than 350 amino acids or having less than 30% identity to the most similar reference sequence, 65 candidate TPSs were identified, of which, 41 TPSs were located in 14 tandem clusters (Supplementary Table S6)."

6) p. 12 l. 259 "asterisks" or "stars" rather than "dots"

Author response: We have edited the text as suggested,

7) Figure 2, how was the expression calculated and what metrics is represented? Same for figure 5 and 6

Author response: We added more details on the transcription analysis in the main text as suggested.

"To generate transcript evidence for genome annotation, raw RNA-seq reads from a previous study were downloaded from NCBI (SRA SRP059970) and adapters and low-quality bases were removed using Cutadapt (v1.8.1) [17] requiring a minimum base quality of 20 and minimum size of 20-nt. The processed reads were aligned to the improved assembly using TopHat2 (v2.0.13) [18] with default parameters."

"To better understand the potential function of these tandem gene clusters, normalized estimation of expression abundances (FPKM) of the annotated teak genes were quantified for the RNA-seq experiments (SRA SRP059970) described above using Cufflinks (v2.2.1) with default parameters [34]. Except for the 12-year-old branch (replicate 1 showed low correlation with other branch samples), the two biological replicates for other branch and stem samples showed high correlations ($r>0.94$, $p<0.0001$, Supplementary Table S4) of gene expression levels; therefore, replicate 2 for the 12-year-old branch and one replicate for other woody tissues were used for downstream analyses."

8) In Figure 2, use the gene name described in the text in addition to the gene IDs.

Author response: Gene name abbreviations were added after the gene IDs in the

Powered by Editorial Manager® and ProduXion Manager® from Aries Systems Corporation

figure (Now Figure 3).

9) p. 6 second paragraph and Figure 3. Discussing the gene family expansion in the light of the WGD would be of interest.

Author response: This is a good suggestion. However, discussion of gene family expansion in the light of WGD would require additional phylogenomic analyses that are beyond the scope of our current manuscript. We plan to investigate this topic in more detail within the context of additional genomes from the Lamiaceae.

10) What do the red and black bar represent in Figure 5? Add the information to the legend.

Author response: These are roman numbers (I, II, and III), which highlight the gene clusters with TPS expression in woody tissues. Information has been added to the legend and main text to clarify this.

11) In Figure 6, the coordinates as well as the scaffold should be indicated in the schematic gene representation

Author response: The scaffold number and coordinates of the region were added in the figure (now Figure 7).

12) Supplementary Table 1 should contain the BUSCO results for the released assembly (Illumina + nanopore)

Author response: We have provided the BUSCO results from the released assembly in Supplementary Table 1.

13) Supplementary Table 3 and 4; add the expression unit in the column header or as a caption.

Author response: We added the expression units in the caption as suggested. Two new supplementary tables were added, so the original Table S4 is now Table S6.

Reviewer #2: The improved version of the teak genome reported here will be a good resource for the forest tree community and for teak in particular. The genome is a great improvement on the previous version and the methods are appropriate. Additional analysis of terpene synthase and phenylpropanoid pathway genes, particularly looking at occurence in tandem copies, highlights the utility of a contiguous, well annotated genome for furthering teak research. Overall, I found the report to be very clear and concise.

My main request to the authors is to expand the depth of the methods, particularly:

- software versions are not given for any packages used, which are typically reported for reproducibility and clarity
Author response: Software versions and other related detailed have been added as suggested.

- line 66 - "modified SNAP read mapper" - how was it modified? Can you make the modifications public?

Author response: The Hi-C scaffolding was performed by Dovetail. They have provided more details ontheir pipeline. The modification to SNAP is “the four non-genomic bases were deleted prior to the mapping.” which has been added to the main text as shown below.
“Shotgun and Dovetail Hi-C library sequences were aligned to the initial assembly using a SNAP read mapper [10] where the four non-genomic bases were deleted prior to the mapping.”

- line 95 - "followed by manual curation" - This is very vague - it needs a bit more

Powered by Editorial Manager® and ProduXion Manager® from Aries Systems Corporation

description of what type of manual curtion and which genes

Author response: More details were added as suggested, which is shown below.

“Genes of interest (e.g., terpene synthases as described below) were manually curated using Apollo (v1.11.8) [22]. ”

- methods for pfam domain identification are missing (hmmer version and pfam db version)

Author response: More details were added as suggested.

“Functional annotation was assigned using BLAST [23] searches against the Arabidopsis thaliana annotation (TAIR10) [24] and Swiss-Prot plant proteins (downloaded on Nov. 17, 2016), and a search against Pfam (v31) [25] using HMMER (v3.1b2) [26] with the cutoff of 1e-5.”

- RNASeq mapping details are missing (what software?) and how was data normalized

Author response: More details were added as suggested.

“To generate transcript evidence for genome annotation, raw RNA-seq reads from a previous study were downloaded from NCBI (SRA SRP059970) and adapters and low-quality bases were removed using Cutadapt (v1.8.1) [17] requiring a minimum base quality of 20 and minimum size of 20-nt. The processed reads were aligned to the improved assembly using TopHat2 (v2.0.13) [18] with default parameters.”

“To better understand the potential function of these tandem gene clusters, normalized estimation of expression abundances (FPKM) of the annotated teak genes were quantified for the RNA-seq experiments (SRA SRP059970) described above using Cufflinks (v2.2.1) with default parameters [34].”

- The RNASeq data used is published: Galeano E et al., "Large-scale transcriptional profiling of lignified tissues in Tectona grandis.", BMC Plant Biol, 2015 Sep 15;15:221

This paper should be cited along with the SRA accessions.

Author response: This citation was added as suggested.

- For figure 2, were expression profiles from biological replicates averaged or normalized in some other way?

Author response: More details were added in the main text to clarify this question, which is also shown below.

“Except for the 12-year-old branch (replicate 1 showed low correlation with other branch samples), the two biological replicates for other branch and stem samples showed high correlations ($r>0.94$, $p<0.0001$, Supplementary Table S4) of gene expression levels; therefore, replicate 2 for the 12-year-old branch and one replicate for other woody tissues were used for downstream analyses. “

- Expression profile colors vary from figure to figure with blue/black/yellow in figures 2 and 5, then white/red in figure 6. It would be good if they were consistent. Also, I find a two color scheme much easier to interpret over the three color blue/black/yellow.

Author Response: All figures are with the same color profiles (blue/black/yellow).

I checked a set of 4 of the Dryad files (teak_hc_models_HiC.cdna_con_sorted_modiGeneID.fa, teak_hc_models_HiC.pep_con_sorted_modiGeneID.fa, teak_hc_models_HiC_con_sorted_modiGeneID.gff, teak_tectona_grandis_26Jun2018_7GlFM_fmt_tp.fa) - all were consistently and properly formatted and matched the details in the paper.

	While Dryad is great, it is still worthwhile to submit the genome and annotation to NCBI or EMBL, where it will be more discoverable and users can take advantage of the many tools available for searching/downloading/exploring sequences.<br><br>Author Response: Thank you for your suggestion. In addition to Dryad, we have now deposited the associated data in the GigaScience database, where readers can easily obtain the files.
**Additional Information:**	
**Question**	**Response**
Are you submitting this manuscript to a special series or article collection?	No
**Experimental design and statistics**<br><br>Full details of the experimental design and statistical methods used should be given in the Methods section, as detailed in our Minimum Standards Reporting Checklist. Information essential to interpreting the data presented should be made available in the figure legends.<br><br>Have you included all the information requested in your manuscript?	Yes
**Resources**<br><br>A description of all resources used, including antibodies, cell lines, animals and software tools, with enough information to allow them to be uniquely identified, should be included in the Methods section. Authors are strongly encouraged to cite Research Resource Identifiers (RRIDs) for antibodies, model organisms and tools, where possible.<br><br>Have you included the information requested as detailed in our Minimum Standards Reporting Checklist?	Yes
**Availability of data and materials**<br><br>All datasets and code on which the conclusions of the paper rely must be either included in your submission or deposited in publicly available repositories	Yes

Powered by Editorial Manager® and ProduXion Manager® from Aries Systems Corporation

(where available and ethically appropriate), referencing such data using a unique identifier in the references and in the "Availability of Data and Materials" section of your manuscript.<br><br>Have you have met the above requirement as detailed in our Minimum Standards Reporting Checklist?	

Manuscript

Click here to access/download;Manuscript;Zhao-Teak-manuscript-gigaSci-revised_10_01_19.docx

Click here to view linked References

A chromosomal-scale genome assembly of *Tectona grandis* reveals the importance of tandem gene duplication and enables discovery of genes in natural product biosynthetic pathways

Dongyan Zhao[1], John P. Hamilton[1], Wajid Waheed Bhat[2,3], Sean R. Johnson[2], Grant T. Godden[4], Taliesin J. Kinser[4,5], Benoît Boachon[6], Natalia Dudareva[6], Douglas E. Soltis[4,5], Pamela S. Soltis[4], Bjoern Hamberger[2], C. Robin Buell[1,7,8,*]

[1]Department of Plant Biology, Michigan State University, East Lansing, MI 48824, USA

[2]Department of Biochemistry and Molecular Biology, Michigan State University, East Lansing, MI 48824, USA

[3]Department of Pharmacology and Toxicology, Michigan State University, East Lansing, MI 48824, USA

[4]Florida Museum of Natural History, University of Florida, Gainesville, FL 32611, USA

[5]Department of Biology, University of Florida, Gainesville, FL 32611, USA

[6]Department of Biochemistry, Purdue University, West Lafayette, IN 47907, USA

[7]Plant Resilience Institute, Michigan State University, East Lansing, MI 48872, USA

[8]MSU AgBioResearch, Michigan State University, East Lansing, MI 48872, USA

Email addresses: Dongyan Zhao <zhaodon4@msu.edu>, John P. Hamilton <jham@msu.edu>, Wajid Waheed Bhat <bhatwaji@msu.edu>, Sean R. Johnson <seanRjohnson@gmail.com>, Grant T. Godden <g0ddengr@ufl.edu>, Taliesin J. Kinser <tkinser@ufl.edu>, Benoît Boachon <benoit.boachon@gmail.com>, Natalia Dudareva <dudareva@purdue.edu>, Douglas E. Soltis <dsoltis@ufl.edu>, Pamela S. Soltis <psoltis@flmnh.ufl.edu>, Bjoern Hamberger <hamberge@msu.edu>, C. Robin Buell <buell@msu.edu>

*Correspondence should be addressed to: C. Robin Buell, buell@msu.edu

Manuscript type: Data note

Abstract

Background: Teak, a member of the Lamiaceae family, produces one of the most expensive hardwoods in the world. High demand coupled with deforestation have caused a decrease in natural teak forests, and future supplies will be reliant on teak plantations. Hence, selection of teak tree varieties for clonal propagation with superior growth performance is of great importance, and access to high-quality genetic and genomic resources can accelerate the selection process by identifying genes underlying desired traits.

Findings: To facilitate teak research and variety improvement, we generated a highly contiguous, chromosomal-scale genome assembly using high-coverage PacBio long reads coupled with high-throughput chromatin conformation capture. Of the 18 teak chromosomes, we generated 17 near-complete pseudomolecules with one chromosome present as two chromosome arm scaffolds. Genome annotation yielded 31,168 genes encoding 46,826 gene models, of which, 39,930 and 41,155 had Pfam domain and expression evidence, respectively. We identified 14 clusters of tandem-duplicated terpene synthases (TPSs), genes central to the biosynthesis of terpenes which are involved in plant defense and pollinator attraction. Transcriptome analysis revealed 10 TPSs highly expressed in woody tissues, of which, 8 were in tandem, revealing the importance of resolving tandemly duplicated genes and the quality of the assembly and annotation. We also validated the enzymatic activity of four TPSs to demonstrate the function of key TPSs.

Conclusions: In summary, this high-quality chromosomal-scale assembly and functional annotation of the teak genome will facilitate the discovery of candidate genes related to traits critical for sustainable production of teak and for anti-insecticidal natural products.

Keywords: Teak, chromosomal-scale assembly, terpene synthases, tandem-duplicated genes

Data Description

Introduction

Teak (*Tectona grandis* L.f.; 2*n* = 2*x* = 36), a member of the angiosperm family Lamiaceae, produces timber of high value due to its durability, hardness, appearance, and resistance to biotic and abiotic stresses (Fig. 1). Teak is one of the most expensive hardwoods in the world, with an average price for high-quality logs ranging from \$600-1000/m^3 USD [1]. High demand coupled with deforestation have caused a decrease in natural teak forests, and future supplies will be reliant on teak plantations. Hence, selection of teak tree varieties for clonal propagation with superior growth performance is of great importance, and access to high-quality genetic and genomic resources can accelerate the selection process by identifying genes underlying desired traits. The only available genome assembly for teak (hereafter referred to as the "released assembly") was completed using short-reads and low-coverage (7x) nanopore long reads [2]; while improved compared to other short-read assembled plant genomes, the released assembly is still highly fragmented, comprising 2,993 scaffolds with the maximum and N50 scaffold length of 1.7 Mb and 358 kbp, respectively.

DNA extraction and genome sequencing

Teak seeds were obtained from Sheffield's Seed Company [3]. High molecular weight DNA was extracted from young leaves of a 2-week-old plant grown in the greenhouse using a modified CTAB method [4]. Long read sequencing was done using Pacific Biosciences RSII and Sequel single-molecule sequencers at the University of Delaware Sequencing & Genotyping Center. Briefly, SMRTbell DNA libraries were constructed from genomic DNA using the SMRTbell Template Prep Kit 1.0-SPv3 as per the manufacturer's instructions (Pacific Biosciences, Menlo Park, CA). The library was size selected using the BluePippin Size-selection system and protocol for 15 Kbp size selection (Sage Science, Amherst, MA). Following size selection, the average library fragment size was 25 kb based on the Fragment Analyzer sizing profile (Advanced Analytical Technologies, Arkeny, IA). The library was sequenced for 6 hours on 10 SMRT cells using P6-C4 chemistry on the PacBio RS II instrument (Pacific Biosciences, Menlo Park, CA) and 10 hours on 4 SMRT cells using 2.0 sequencing chemistry on the PacBio Sequel instrument (Pacific Biosciences, Menlo Park, CA). A total of ~4.7 million PacBio long reads were generated, which is ~104x coverage of the estimated 325 Mbp teak genome. Additionally,

whole genome short-read sequencing libraries were generated using Illumina TruSeq Nano DNA Library Preparation Kit (Cat. No. FC-121-4001) and sequenced to 150-nt paired end reads on Illumina HiSeq 4000.

Genome assembly and quality assessment

The raw reads were error-corrected using Canu (Canu, RRID:SCR_015880) v1.6 [5] (canu -correct) and trimmed (canu -trim) for low-quality bases and reads ≥ 1 kb were used to generate the initial assembly (canu -assemble) with a correctedErrorRate of 0.09%. The assembly consists of 1,474 contigs with a total length of 338 Mbp, 20 Mbp larger than the released assembly (Tables 1 and 2). The initial assembly was polished using the raw PacBio reads using Arrow in the SMRT Analysis package v5.0.1.9585 [6], followed by three rounds of error correction with 643.7 million Illumina short reads (570x coverage, Table 3) using Pilon (Pilon, RRID:SCR_014731) v1.13 [7]. A Dovetail Hi-C library was prepared as described previously [8]. The resulting library had a double restriction site signature, where four non-genomic bases were introduced. The initial PacBio assembly, shotgun reads, and Dovetail Hi-C library reads were used as input data for scaffolding using HiRise [9]. Shotgun and Dovetail Hi-C library sequences were aligned to the initial assembly using a SNAP read mapper [10] where the four non-genomic bases were deleted prior to the mapping. The separation of aligned Dovetail Hi-C read pairs were analyzed by HiRise to produce a likelihood model for genomic distance between read pairs, and the model was used to identify and break putative mis-joins, to score prospective joins, and make joins above a threshold. The Hi-C scaffolding resulted in 936 scaffolds (referred to as “improved assembly”, hereafter), with an N50 scaffold size of 18.5 Mbp, which is a 46x improvement of genome contiguity over the released assembly (Tables 1 and 2). The 19 largest scaffolds (minimum length of 8.6 Mbp) represented 90% of the assembled 338 Mbp genome; of the 18 teak chromosomes, we generated 17 near-complete pseudomolecules with one chromosome present as two chromosome arm scaffolds (Fig. 2). The completeness of our improved assembly was also demonstrated by the presence of tandem tracts of the telomere repeat sequence in nine of the 19 pseudomolecules; two pseudomolecules contained telomere tracks at both ends (Fig. 2). A tandem array of 5S rRNA sequence (135 copies with each at 496 bp) was found in pseudomolecule 10 spanning >67.5 kbp, highlighting the power of long reads in resolving highly repetitive sequences. Around 98% of the whole genome shotgun reads

aligned to the improved assembly, of which, 94 - 98% of the reads were properly paired (Table 3). The representation of genic sequences in our improved assembly was confirmed by detection of 94.4% of the Benchmarking Universal Single-Copy Orthologs (BUSCO, RRID:SCR_015008) v2.0 [11]; Complete:92.3%[Single-copy:82.4%,Duplicated:9.9%], Fragmented:2.1%, Missing:5.6%, Total BUSCO groups searched:1440; Supplementary Table S1) and by alignment of 89% - 93% of transcriptome reads from publicly available RNA-seq datasets derived from diverse tissues of other teak accessions [12] (NCBI SRA SRP059970; Supplementary Table S2).

Genome annotation

A custom repeat library (CRL) was generated for teak by running RepeatModeler (RepeatModeler, RRID:SCR_015027) v1.0.8 [13], excluding protein-coding genes using ProtExcluder (v1.1) [14], and adding the Viridiplantae RepBase repeats [15]. The improved assembly was masked with the CRL using RepeatMasker (RepeatMasker, RRID:SCR_012954) v4.0.6 with default parameters [16], which revealed that 32.02% of the improved assembly was identified as repetitive sequence, 3-fold more compared to that reported in the released assembly (11%). To generate transcript evidence for genome annotation, raw RNA-seq reads from a previous study were downloaded from NCBI (SRA SRP059970) and adapters and low-quality bases were removed using Cutadapt (v1.8.1) [17] requiring a minimum base quality of 20 and minimum size of 20-nt. The processed reads were aligned to the improved assembly using TopHat2 (v2.0.13) [18] with default parameters. Genome-guided transcript assemblies for each aligned RNA-seq library were created using Trinity (Trinity, RRID:SCR_013048) v2.2.0 [19] using the default parameters. Gene models were predicted using Augustus (Augustus: Gene Prediction, RRID:SCR_008417) v3.1 [20] by first training Augustus with the leaf RNA-seq alignments, then generating gene predictions on the hard-masked genome. The predicted gene models were refined by running PASA2 v2.1.0 [21] using the genome-guided transcript assemblies and two rounds of annotation comparison. Genes of interest (e.g., terpene synthases as described below) were manually curated using Apollo v1.11.8 [22]. The final working set of annotations was comprised of 31,168 loci and 46,826 gene models. Functional annotation was assigned using BLAST [23] searches against the *Arabidopsis thaliana* (L.) Heynh annotation (TAIR10) [24] and Swiss-Prot plant proteins (downloaded on Nov. 17, 2016), and a search against Pfam v31 [25] using HMMER v3.1b2 [26] with a cutoff of 1e-5. A high confidence subset of the working gene model set was identified by identifying models with an FPKM

(fragments per kilobase of exon model per million reads mapped, a normalized estimation of gene expression abundance) > 0 in any of the RNA-Seq libraries or a match in Pfam (v31). The high confidence gene model set is comprised of 41,155 gene models and 39,930 loci.

Detection of whole genome duplication events

Whole-genome duplications (WGD) can contribute to genetic innovations underlying chemical defense against co-evolving insect herbivores, as exemplified by evidence from studies of other plant groups (e.g., Brassicales [27]). To infer WGD events in teak, we used the DupPipe pipeline with default settings [28] to analyze coding sequences representing the longest isoforms of genes (Supplemental Information). Gaussian mixture models predicted three components within the observed *Ks* distribution of teak, with mean values at K_S = 0.22, 0.60, 1.36 (Supplementary Fig. S1A). These components were further compared with results from a SiZer analysis [29] (implemented with the 'multimode' R statistical package [30]), which distinguishes true data features from noise by testing for significant increases or decreases, or no significant changes across an observed K_S distribution at various bandwidths (Supplemental Information). Of the three peaks identified with mixture models, only a peak at K_S = 0.60 was corroborated as a significant feature by a SiZer analysis (Supplementary Fig. S1B), providing evidence for at least one WGD event in teak. Whether or not this WGD event is lineage-specific or shared by other Lamiaceae is a subject of active research.

The phenylpropanoid pathway genes and their expression

Teak is known for strong wood, and we were able to identify all of the genes involved in the phenylpropanoid pathway which leads to lignin formation (Supplementary Table S3). Using phenylpropanoid pathway genes in *A. thaliana* [31] as bait, the corresponding candidate genes in teak were identified based on orthology analysis between teak and *A. thaliana* using OrthoFinder v2.0 with default parameters [32]. The phenylpropanoid pathway genes are often found in physical clusters [33] and we defined physical clusters of genes if: 1) there were no more than 10 genes in between on a single pseudomolecule and 2) the pairwise gene distance was less than 100 kbp. Notably, four of the 11 core genes in the phenylpropanoid pathway were present in tandem copies, with shikimate O-hydroxycinnamoytransferase (HCT) having three tandem clusters of two copies each and one cluster of five copies (Fig. 3). To better understand the potential function of these tandem gene clusters, normalized estimation of expression

abundances (FPKM) of the annotated teak genes were quantified for the RNA-seq experiments (SRA SRP059970) described above using Cufflinks (Cufflinks, RRID:SCR_014597) v2.2.1 with default parameters [34]. Except for the 12-year-old branch (replicate 1 showed low correlation with other branch samples), the two biological replicates for other branch and stem samples showed high correlations (*r*>0.94, p<0.0001, Supplementary Table S4) of gene expression levels; therefore, replicate 2 for the 12-year-old branch and one replicate for other woody tissues were used for downstream analyses. For 20 of the 45 genes in the phenylpropanoid pathway, clear neofunctionalization at the expression level was observed for F5H, COMT, PAL, and HCT. Interestingly, cinnamyl CoA reductase (CCR), which catalyzes the first committed step of the lignin-specific branch, was in a physical cluster with five copies of HCT; within this physical cluster, only one of the five HCT genes (Tg16g10070) and CCR (Tg16g10210) were constitutively expressed in all tissues (Fig. 3).

Identification of terpene synthases (TPSs) and functional verification

Terpenes are a large class of specialized metabolites involved in plant defense and pollinator attraction [35]. Terpene synthases (TPSs) are key genes involved in terpenoid biosynthesis and are often found in physical clusters in the genome [36]. A sequence similarity search using BLASTP (v2.2.31+ with default parameters) [23] was performed using the teak peptide models against a set of reference TPS peptides (Supplementary Table S5). After filtering out teak peptides shorter than 350 amino acids or having less than 30% identity to the most similar reference sequence, 65 candidate TPSs were identified, of which, 41 TPSs were located in 14 tandem clusters (Supplementary Table S6). Phylogenetic analysis of teak TPSs and those from *A. thaliana* and *Eucalyptus grandis* W. Hill ex Maiden indicate that multiple recent species-specific tandem duplication events contributed to an expansion in TPS number in teak, consistent with previous findings [37] (Fig. 4; Supplementary Information). Twelve teak TPSs were expressed in stems; seven of these are tandemly duplicated, suggesting these recent tandemly duplicated genes may retain similar functions (Supplementary Table S6). To validate our TPS annotation, four teak diterpene synthases (diTPSs) were amplified from leaf tissues and tested for functional verification through transient expression in *Nicotiana benthamiana* Domin (Supplementary Information). The results demonstrated that TgTPS6 (Tg14g12740) catalyzed the formation of *ent*-copalyl diphosphate, while TgTPS2 (Tg02g10330) converted that product to *ent*-kaurene in the first committed steps of gibberellic acid hormone biosynthesis (Fig. 5; Supplementary Fig.

S2). TgTPS5 (Tg05g04010) and TgTPS1 (Tg05g04000) are located adjacent to each other on the genome and form the pathway to miltiradiene (Fig. 5), an intermediate in the biosynthesis of defense-related specialized metabolites found in many members of Lamiaceae.

Transcriptomic analysis of TPSs and cytochrome P450 enzymes

Transcriptomic analysis of diverse tissues of teak, including leaves, flowers, roots, seedling, and branch and stem secondary xylem of different ages, revealed seven putative monoterpene synthases from subfamily TPS-b (Fig. 6, clades I and II) and three putative sesquiterpene synthases from subfamily TPS-a (Fig. 6, clade III) that were highly expressed in woody tissues, including 12- and 60-year-old branches and stems (Fig. 6). These TPSs are likely responsible for the synthesis of defense-related compounds, including unknown, specialized metabolites that contribute to the termite resistance and defense of wood tissues from other pests and pathogens in teak [38]. Most specialized metabolites, including terpenes, require cytochrome P450 enzymes (CYPs) that modify the terpene scaffold; similar to TPSs, CYPs are often found in physical clusters in the genome [10]. Through sequence similarity searches, 377 CYP genes were identified, of which, 248 (66%) occurred in physical clusters (Supplementary Table S5). In addition, many TPSs and CYPs were clustered together, i.e., of 65 TPSs and 377 CYPs, 20 TPSs and 31 CYPs were co-located in 12 physical clusters. For example, a cluster on pseudomolecule 5 consisted of two TPSs (TPS-e, TPS-c) and eight complete and two partial CYP genes (i.e., four copies of CYP76AH, four copies of CYP71D, and two copies of CYP714G). Similar to the pattern observed for lignin pathway genes, neofunctionalization of expression across tissues was observed for the CYP subfamily genes (Fig. 7). It is notable that a putative TPS-e (Tg05g04000) was constitutively expressed in all tissues examined and a putative TPS-c (Tg05g04010) was co-regulated with a putative CYP76AH31 (Tg05g04020) (Fig. 7). From a biochemical perspective, subfamily CYP76AH contains several P450s that are involved in (di)terpene specialized metabolism and occur in close physical proximity in other species [36,39]. In another species of Lamiaceae, *Salvia miltiorrhiza* Bunge, the best match for the teak TPS-c/CYP76AH31 cluster was the SmCPS1/CYP76AH12 gene cluster (Fig. 7), which is involved in the biosynthesis of tanshinone diterpenes and organized in several gene clusters, suggesting physical clustering is a major mechanism regulating expression of genes involved in the same biosynthetic pathway in plants [40].

Conclusion

In summary, we generated a chromosomal-scale assembly of the teak genome that, when coupled with high-quality functional annotation, will facilitate the discovery of candidate genes related to traits critical for sustainable production of teak and for anti-insecticidal natural products. Furthermore, the high contiguity of our improved assembly will permit comparative genomics studies and exploration of physical gene clustering, facilitating discovery of key biosynthetic pathways.

Availability of supporting data

All sequences generated in this study, including PacBio long reads and Illumina short reads, were deposited in the NCBI SRA under BioProject PRJNA493753. The genome assembly, annotation files, expression matrix, and other supporting data can be accessed at the *GigaScience* database GigaDB [41] and via Dryad [42].

Abbreviations

caffeic acid O-methyltransferases (COMT), Ccaffeoyl Shikimate Esterase (CSE), cetyl trimethylammonium bromide (CTAB), cinnamate 4- hydroxylase (C4H), cinnamyl CoA reductase (CCR), cinnamyl-alcohool dehydrogenases (CAD)cinnamoyl CoA O-methyltransferases (CCoAOMT), coumarate CoA ligases (4CL), custom repeat library (CRL), cytochrome P450 enzymes (CYPs), di-terpene synthase (di-TPS), ferulate 5-hydroxylases (F5H), p-coumarate 3-hydroxylase (C3H), phenylalanine ammonia lyase (PAL), RNA-sequencing (RNA-seq), shikimate O-hydroxycinnamoytransferase (HCT), single molecule real time sequencing (SMRT sequencing), terpene synthase (TPS), whole genome duplications (WGD)

Competing interests

The authors have declared that no competing interests exists.

Funding

Funds for this study were provided by a grant to CRB, ND, DS, and PS from the National Science Foundation Plant Genome Research Program (IOS-1444499) and from Hatch funds to CRB.

Author contributions

C.R.B, B.H., and D.Z. designed the experiment, D.Z. and J.P.H. conducted genome assembly and annotation, D.Z. generated expression matrix and physical clustering of TPSs/CYPs, W.W.B. and S.R.J. conducted the TPS phylogeny and functional verification of 4 TPSs, G.G. and T.K. conducted whole-genome duplication analysis, B.B. analyzed TPS expression, C.R.B., B.H., P.S., D.S., and N.D. provided intellectual insights and supervised the work. All authors read and wrote part of the manuscript.

Acknowledgements

We thank Dr. David Nelson (The University of Tennessee, Health Science Center) for annotating the new P450s. We thank Krystle Wiegert-Rininger and Joshua Wood for taking care of the teak plants. We thank Brieanne Vaillancourt for handling the data storage and submission and providing valuable comments on the manuscript.

References

[1] Food and Agriculture Organization of the United Nations, Global teak trade in the aftermath of Myanmar's log export ban, 2015. http://www.fao.org/3/a-i5023e.pdf.

[2] R. Yasodha, R. Vasudeva, S. Balakrishnan, A.R. Sakthi, N. Abel, N. Binai, B. Rajashekar, V.K.W. Bachpai, C. Pillai, S.A. Dev, Draft genome of a high value tropical timber tree, Teak (Tectona grandis L. f): insights into SSR diversity, phylogeny and conservation., DNA Res. 25 (2018) 409–419.

[3] Sheffield's Seed Company, Sheffield's Seed Company,. https://sheffields.com/.

[4] J.J. Doyle, Isolation of plant DNA from fresh tissue. Focus, Focus (Madison). 12 (1990) 13–15.

[5] S. Koren, B.P. Walenz, K. Berlin, J.R. Miller, N.H. Bergman, A.M. Phillippy, Canu: scalable and accurate long-read assembly via adaptive k-mer weighting and repeat separation., Genome Res. 27 (2017) 722–736.

[6] Pacfici Biosciences, SMRT tools. https://www.pacb.com/wp-content/uploads/SMRT-Tools-Reference-Guide-v4.0.0.pdf.

[7] B.J. Walker, T. Abeel, T. Shea, M. Priest, A. Abouelliel, S. Sakthikumar, C.A. Cuomo, Q. Zeng, J. Wortman, S.K. Young, A.M. Earl, Pilon: An Integrated Tool for Comprehensive Microbial Variant Detection and Genome Assembly Improvement, PLoS One. 9 (2014) e112963.

[8] E. Lieberman-Aiden, N.L. van Berkum, L. Williams, M. Imakaev, T. Ragoczy, A. Telling, I. Amit, B.R. Lajoie, P.J. Sabo, M.O. Dorschner, R. Sandstrom, B. Bernstein, M.A. Bender, M. Groudine, A. Gnirke, J. Stamatoyannopoulos, L.A. Mirny, E.S. Lander, J. Dekker, Comprehensive mapping of long-range interactions reveals folding principles of the human genome., Science. 326 (2009) 289–293.

[9] N.H. Putnam, B.L. O'Connell, J.C. Stites, B.J. Rice, M. Blanchette, R. Calef, C.J. Troll, A. Fields, P.D. Hartley, C.W. Sugnet, D. Haussler, D.S. Rokhsar, R.E. Green, Chromosome-scale shotgun assembly using an in vitro method for long-range linkage, Genome Res. 26 (2016) 342–350.

[10] The UC Berkeley AMP Lab, Scalable Nucleotide Alignment Program,. http://snap.cs.berkeley.edu.

[11] F.A. Simão, R.M. Waterhouse, P. Ioannidis, E. V. Kriventseva, E.M. Zdobnov, BUSCO: assessing genome assembly and annotation completeness with single-copy orthologs, Bioinformatics. 31 (2015) 3210–3212.

[12] E. Galeano, T.S. Vasconcelos, M. Vidal, M.K. Mejia-Guerra, H. Carrer, Large-scale transcriptional profiling of lignified tissues in *Tectona grandis*, BMC Plant Biol. 15 (2015) 221.

[13] A. Smit, R. Hubley, *RepeatModeler Open-1.0.*, (2008). http://www.repeatmasker.org.

[14] ProtExcluder, https://doi.org/10.1104/pp.113.230144.

[15] J. Jurka, Repeats in genomic DNA: mining and meaning, Curr. Opin. Struct. Biol. 8 (1998) 333–337.

[16] A. Smit, R. Hubley, P. Green, *RepeatMasker Open-4.0.*, (2013).

[17] M. Martin, Cutadapt removes adapter sequences from high-throughput sequencing reads, EMBnet.Journal. 17 (2011) 10–12.

[18] D. Kim, G. Pertea, C. Trapnell, H. Pimentel, R. Kelley, S.L. Salzberg, TopHat2: accurate alignment of transcriptomes in the presence of insertions, deletions and gene fusions, Genome Biol. 14 (2013) R36.

[19] N. Manfred G. Grabherr, Brian J. Haas, Moran Yassour, Joshua Z. Levin, Dawn A. Thompson, Ido Amit, Xian Adiconis, Lin Fan, Raktima Raychowdhury, Qiandong Zeng, Zehua Chen, Evan Mauceli, Nir Hacohen, Andreas Gnirke, Nicholas Rhind, Federica di Palma, Bruce W., and A.R. Friedman, Trinity: reconstructing a full-length transcriptome without a genome from RNA-Seq data, Nat. Biotechnol. 29 (2013) 644–652.

[20] M. Stanke, S. Waack, Gene prediction with a hidden Markov model and a new intron submodel, Bioinformatics. 19 (2003) ii215-ii225.

[21] M.A. Campbell, B.J. Haas, J.P. Hamilton, S.M. Mount, C.R. Buell, Comprehensive analysis of alternative splicing in rice and comparative analyses with Arabidopsis, BMC Genomics. 7 (2006) 327.

[22] E. Lee, N. Harris, M. Gibson, R. Chetty, S. Lewis, Apollo: a community resource for genome annotation editing, Bioinformatics. 25 (2009) 1836–1837.

[23] C. Camacho, G. Coulouris, V. Avagyan, N. Ma, J. Papadopoulos, K. Bealer, T.L. Madden, BLAST+: architecture and applications, BMC Bioinformatics. 10 (2009) 421.

[24] The Arabidopsis Information Resource, https://arabidopsis.org.

[25] S. El-Gebali, J. Mistry, A. Bateman, S.R. Eddy, A. Luciani, S.C. Potter, M. Qureshi, L.J. Richardson, G.A. Salazar, A. Smart, E.L.L. Sonnhammer, L. Hirsh, L. Paladin, D. Piovesan, S.C.E. Tosatto, R.D. Finn, The Pfam protein families database in 2019, Nucleic Acids Res. (2018).

[26] HMMER,. http://hmmer.org/.

[27] P.P. Edger, H.M. Heidel-Fischer, M. Bekaert, J. Rota, G. Glöckner, A.E. Platts, D.G. Heckel, J.P. Der, E.K. Wafula, M. Tang, J.A. Hofberger, A. Smithson, J.C. Hall, M. Blanchette, T.E. Bureau, S.I. Wright, C.W. dePamphilis, M.E. Schranz, M.S. Barker, G.C. Conant, N. Wahlberg, H. Vogel, J.C. Pires, C.W. Wheat, The butterfly plant arms-race escalated by gene and genome duplications, Proc. Natl. Acad. Sci. 112 (2015) 8362–8366.

[28] M.S. Barker, K.M. Dlugosch, L. Dinh, R.S. Challa, N.C. Kane, M.G. King, L.H. Rieseberg, EvoPipes.net: Bioinformatic Tools for Ecological and Evolutionary Genomics, Evol. Bioinforma. 6 (2010) EBO.S5861.

[29] P. Chaudhuri, J.S. Marron, SiZer for Exploration of Structures in Curves, J. Am. Stat. Assoc. 94 (1999) 807.

[30] J. Ameijeiras-Alonso, R.M. Crujeiras, A. Rodríguez-Casal, Multimode: An R Package for Mode Assessment, (2018). http://arxiv.org/abs/1803.00472.

[31] R. Caspi, R. Billington, C.A. Fulcher, I.M. Keseler, A. Kothari, M. Krummenacker, M. Latendresse, P.E. Midford, Q. Ong, W.K. Ong, S. Paley, P. Subhraveti, P.D. Karp, The MetaCyc database of metabolic pathways and enzymes, Nucleic Acids Res. 46 (2018) D633–D639.

[32] D.M. Emms, S. Kelly, OrthoFinder: solving fundamental biases in whole genome comparisons dramatically improves orthogroup inference accuracy, Genome Biol. 16

(2015) 157.

[33] A.A. Myburg, D. Grattapaglia, G.A. Tuskan, U. Hellsten, R.D. Hayes, J. Grimwood, J. Jenkins, E. Lindquist, H. Tice, D. Bauer, D.M. Goodstein, I. Dubchak, A. Poliakov, E. Mizrachi, A.R.K. Kullan, S.G. Hussey, D. Pinard, K. van der Merwe, P. Singh, I. van Jaarsveld, O.B. Silva-Junior, R.C. Togawa, M.R. Pappas, D.A. Faria, C.P. Sansaloni, C.D. Petroli, X. Yang, P. Ranjan, T.J. Tschaplinski, C.-Y. Ye, T. Li, L. Sterck, K. Vanneste, F. Murat, M. Soler, H.S. Clemente, N. Saidi, H. Cassan-Wang, C. Dunand, C.A. Hefer, E. Bornberg-Bauer, A.R. Kersting, K. Vining, V. Amarasinghe, M. Ranik, S. Naithani, J. Elser, A.E. Boyd, A. Liston, J.W. Spatafora, P. Dharmwardhana, R. Raja, C. Sullivan, E. Romanel, M. Alves-Ferreira, C. Külheim, W. Foley, V. Carocha, J. Paiva, D. Kudrna, S.H. Brommonschenkel, G. Pasquali, M. Byrne, P. Rigault, J. Tibbits, A. Spokevicius, R.C. Jones, D.A. Steane, R.E. Vaillancourt, B.M. Potts, F. Joubert, K. Barry, G.J. Pappas, S.H. Strauss, P. Jaiswal, J. Grima-Pettenati, J. Salse, Y. Van de Peer, D.S. Rokhsar, J. Schmutz, The genome of *Eucalyptus grandis*, Nature. 510 (2014) 356–362.

[34] C. Trapnell, A. Roberts, L. Goff, G. Pertea, D. Kim, D.R. Kelley, H. Pimentel, S.L. Salzberg, J.L. Rinn, L. Pachter, Differential gene and transcript expression analysis of RNA-seq experiments with TopHat and Cufflinks, Nat. Protoc. 7 (2012) 562–578.

[35] N. Dudareva, A. Klempien, J.K. Muhlemann, I. Kaplan, Biosynthesis, function and metabolic engineering of plant volatile organic compounds, New Phytol. 198 (2013) 16–32.

[36] A.M. Boutanaev, T. Moses, J. Zi, D.R. Nelson, S.T. Mugford, R.J. Peters, A. Osbourn, Investigation of terpene diversification across multiple sequenced plant genomes., Proc. Natl. Acad. Sci. U. S. A. 112 (2015) E81-8.

[37] Mint Evolutionary Genomics Consortium, Phylogenomic Mining of the Mints Reveals Multiple Mechanisms Contributing to the Evolution of Chemical Diversity in Lamiaceae, Mol. Plant. 11 (2018) 1084–1096.

[38] V. Pandey, A.K. Singh, R.P. Sharma, Biodiversity of Insect Pests associated with Teak (*Tectona grandis* L.f.) in Eastern Uttar Pradesh of India, Res. J. For. 4 (2010) 136–144.

[39] J. Zi, Y. Matsuba, Y.J. Hong, A.J. Jackson, D.J. Tantillo, E. Pichersky, R.J. Peters,

Biosynthesis of lycosantalonol, a cis-prenyl derived diterpenoid, J. Am. Chem. Soc. 136 (2014) 16951–3.

[40] H. Xu, J. Song, H. Luo, Y. Zhang, Q. Li, Y. Zhu, J. Xu, Y. Li, C. Song, B. Wang, W. Sun, G. Shen, X. Zhang, J. Qian, A. Ji, Z. Xu, X. Luo, L. He, C. Li, C. Sun, H. Yan, G. Cui, X. Li, X. Li, J. Wei, J. Liu, Y. Wang, A. Hayward, D. Nelson, Z. Ning, R.J. Peters, X. Qi, S. Chen, Analysis of the Genome Sequence of the Medicinal Plant *Salvia miltiorrhiza*, Mol. Plant. 9 (2016) 949–52.

[41] Zhao D, Hamilton JP, Bhat WW, Johnson SR, Godden GT, Kinser TJ, et al. Supporting data for "A chromosomal-scale genome assembly of Tectona grandis reveals the importance of tandem gene duplication and enables discovery of genes in natural product biosynthetic pathways" GigaScience Database 2019. http://dx.doi.org/10.5524/100550

[42] Zhao D, Hamilton JP, Bhat WW, Johnson SR, Godden GT, Kinser TJ, et al. Data from: "A chromosomal-scale genome assembly of Tectona grandis reveals the importance of tandem gene duplication and enables discovery of genes in natural product biosynthetic pathways". Dryad Digital Repository 2019. https://doi.org/10.5061/dryad.77b2422

Figure legends

Figure 1. A young teak tree.
Photo taken by Phong Ek [CC BY 2.0 (https://creativecommons.org/licenses/by/2.0)], via Wikimedia Commons

Figure 2. Gene and repeat density across the 19 pseudomolecules in the assembly. Green asterisks denote telomere tracks.

Figure 3. Differential expression of tandem copies of genes in lignin biosynthetic pathway. stem12yr: stem secondary xylem of a 12-year-old teak tree; stem60yr: stem secondary xylem of a 60-year-old teak tree; branch12yr: branch secondary xylem of a 12-year-old teak tree; branch60yr: branch secondary xylem of a 60-year-old teak tree.

Figure 4. Maximum likelihood tree of peptide sequences of terpene synthase (TPS) family genes from the *Tectona grandis* (red branches), *Arabidopsis thaliana* (green branches), and *Eucalyptus grandis* (blue branches). Red dots denote teak TPSs expressed in stems.

Figure 5. Proposed diterpene pathway based on functional validation.

Figure 6. Expression of terpene synthases (TPSs) in various tissues of teak. Six monoterpene synthases (clade I & II as denoted on the nodes) and three putative sesquiterpene synthases (clade III) exhibited high expression in branches and stems of 12- and 60-year-old teak trees.

Figure 7. A physical cluster of TPS/CYP genes on pseudomolecule 5 and their expression in different tissues of teak. Horizontal arrows denote genes with their gene classification listed above and gene IDs below, where unfilled arrows denote partial genes and black arrows denote genes that are not TPS/CYP.

Tables

Table 1. Metrics of contigs and scaffolds of the current assembly.

	Initial assembly using PacBio reads (contigs)	Assembly after Hi-C scaffolding (scaffolds)
Total sequences	1,474	936
Total size (bp)	338,318,549	338,300,341
Maximum sequence size (bp)	21,267,566	20,661,910
Minimum sequence size (bp)	1,168	1,168
N50 sequence size (bp)	3,749,470	16,483,567
N90 sequence size (bp)	52,675	463,203
Average sequence size (bp)	229,524	361,432

Table 2. Cumulative size of contigs and scaffolds of the current assembly.

Initial assembly using PacBio reads (contigs)			
Contig size	Total size (bp)	%Total assembly	# Contigs
≥1 Mbp	248,187,558	73.37	64
≥0.5 Mbp	267,412,682	79.06	91
≥0.1 Mbp	291,028,790	86.04	198
≥0.05 Mbp	305,851,391	90.42	420

Assembly after Hi-C scaffolding (scaffolds)			
Scaffold size	Total size (bp)	%Total assembly	# Scaffolds
≥1 Mbp	304,435,280	89.99	19
≥0.5 Mbp	304,435,280	89.99	19
≥0.1 Mbp	308,724,809	91.26	41
≥0.05 Mbp	314,467,503	92.96	134

412 **Table 3**. Whole genome shotgun reads.

Sample name	NCBI SRA Run ID	QC-passed reads	Mapped	Properly paired out of total reads
Teak_TruSeq_01	SRR7984127	168,566,966	165,783,328 (98.35%)	163,390,358 (97.40%)
Teak_TruSeq_02	SRR7984127	188,504,116	185,541,771 (98.43%)	182,934,854 (97.15%)
TEC_AA_01	SRR7984129	371,978,214	364,473,434 (97.98%)	357,722,188 (96.65%)
TEC_AA_02	SRR7984129	394,477,964	386,545,305 (97.99%)	379,620,884 (96.72%)
TEC_AB_01	SRR7984130	89,116,777	87,087,277 (97.72%)	84,001,838 (94.93%)
TEC_AB_02	SRR7984130	81,436,054	79,540,000 (97.67%)	76,733,986 (94.89%)

413

414

Additional files

Supplementary tables

Table S1. BUSCO results.

This is available as a separate XLS file.

Table S2. Mapping of RNA-seq reads to the assembly.

This is available as a separate XLS file.

Table S3. Genes involved in the core phenylpropanoid biosynthetic pathway and their expression abundance (FPKM: fragments per kilobase of exon model per million reads mapped) in *Tectona grandis*.

This is available as a separate XLS file.

Table S4. Gene expression correlations between tissues and biological replicates (NCBI SRA SRP059970)

This is available as a separate XLS file.

Table S5. Terpene synthases (TPSs) used as references for identification of teak TPSs.

This is available as a separate XLS file.

Table S6. Tandem clusters of candidate terpene synthases and CYPs and their expression abundance (FPKM: fragments per kilobase of exon model per million reads mapped) in *Tectona grandis*.

This is available as a separate XLS file.

Supplementary figures

Figure S1. Inference of ancient WGDs in *Tectona grandis*. (A) Histogram (K_S plot) showing the age distribution of putative paralogous gene pairs overlaid with mixture models of inferred WGD events. The mixture model with an inferred peak at K_S = 0.60 (red) was corroborated by SiZer analysis (Chaudhuri and Marron, 1999), while modeled peaks at K_S = 0.22, 1.36 (blue) were not. (B) SiZer map displaying significant features in the observed K_S distribution at varying

bandwidths. As indicated in the key, colors signify either a significant increase (blue), significant decrease (red), or no significant change (purple) in the data distribution.

Figure S2. Activities of diterpene synthases after transient expression in *Nicotiana benthamiana*. On the left are total ion chromatograms of hexane extracts from plant leaves. On the right are mass spectra from individual peaks. Controls express CfDXS and CfGGPPS, but no recombinant TPS. Hexane extract from the moss *Physcomitrella patens* was used as a standard for *ent*-kaurene. *Zea mays* ZmAN2 (Genbank: AY562491) is a known *ent*-copalyl diphosphate synthase. *Coleus forskohlii* CfTPS1 (Genbank: KF444506), and CfTPS3 (Genbank: KF444508) are known (+)-copalyl diphosphate and miltiradiene synthases, respectively.

Figure 1

Click here to access/download;Figure;Figure 1.pdf

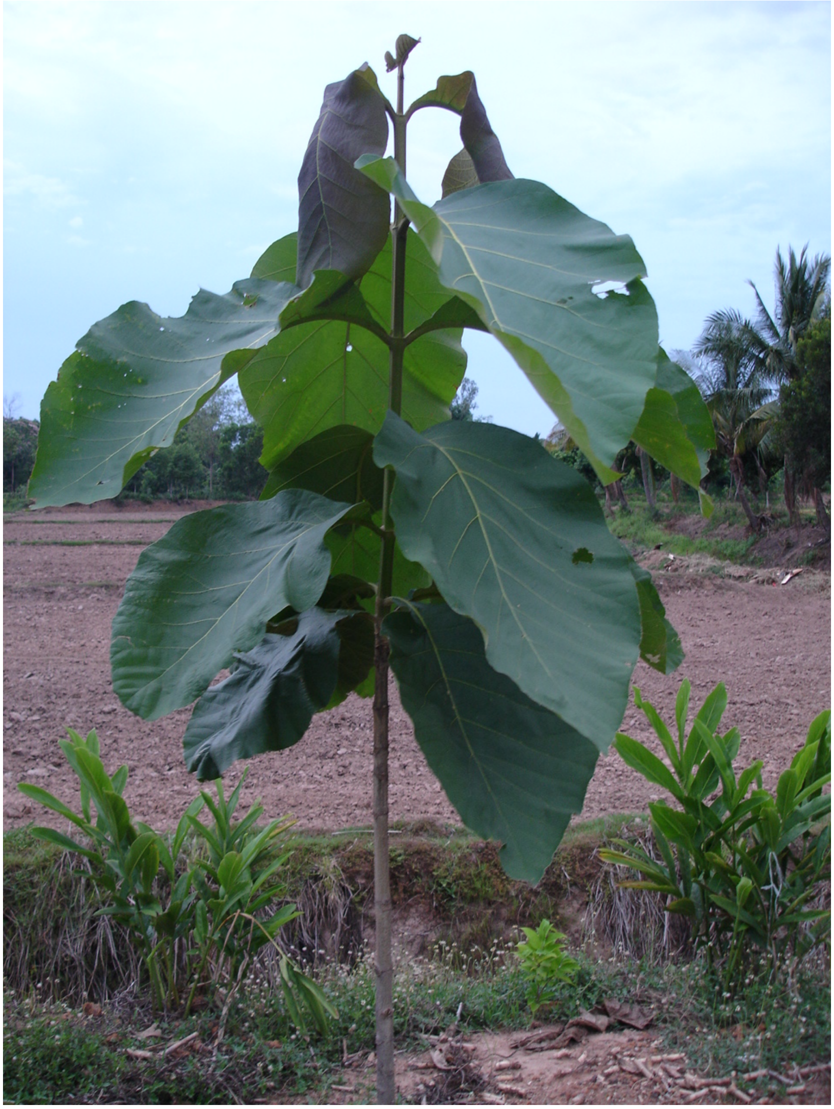

Figure 2

Click here to access/download;Figure;Figure 2.pdf

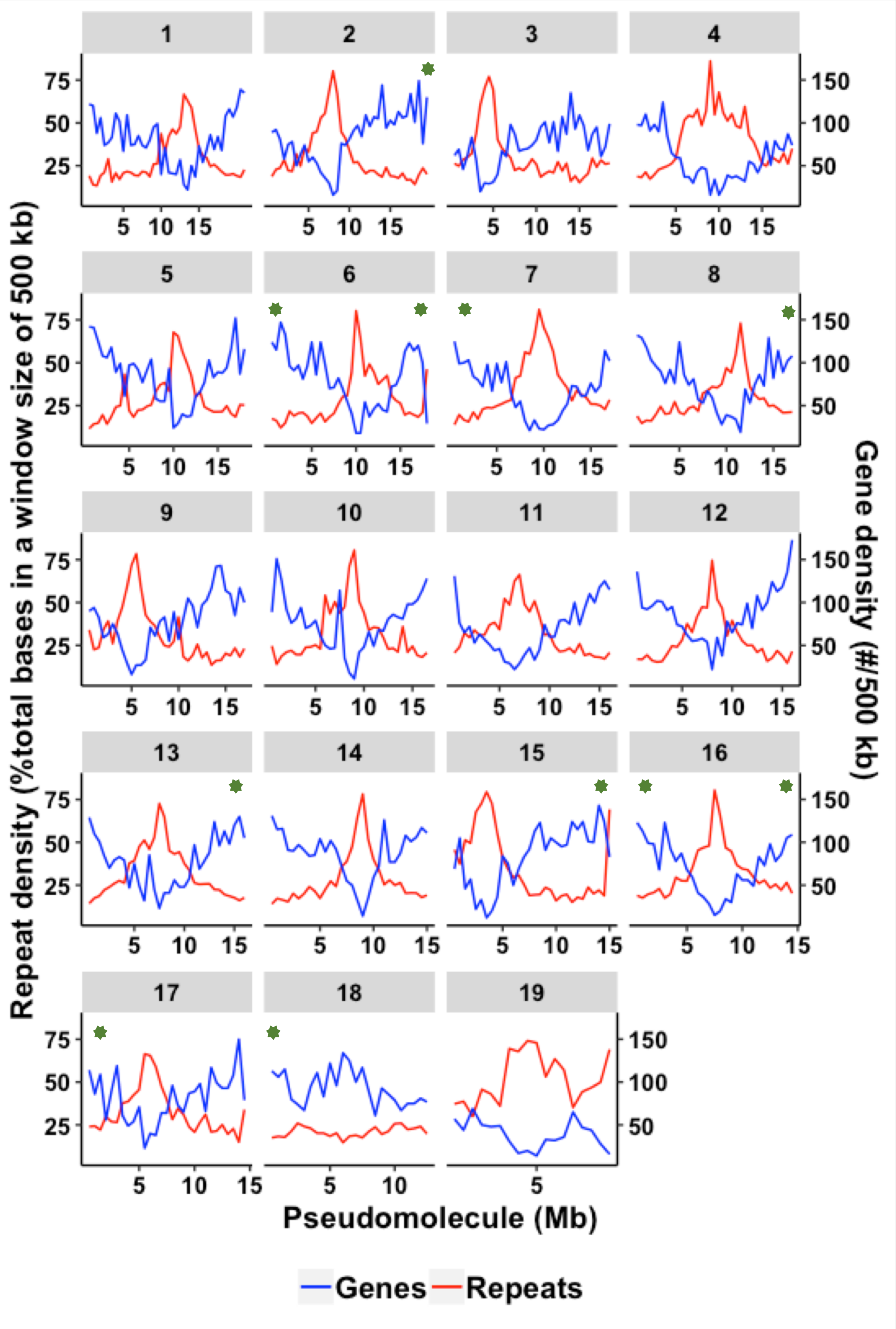

Figure 3

Click here to access/download;Figure;Figure 3.pdf

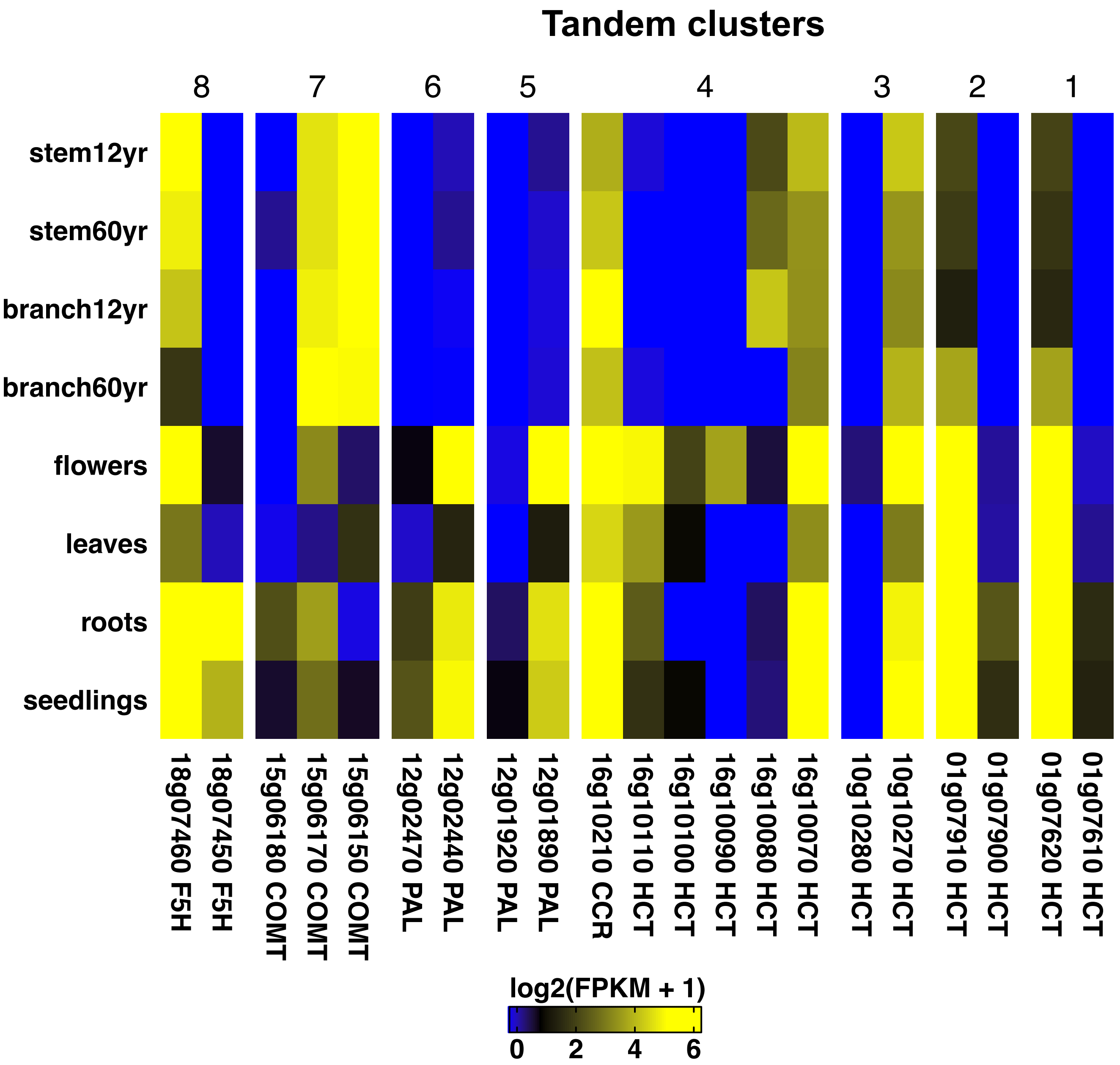

Click here to access/download;Figure;Figure 4.pdf

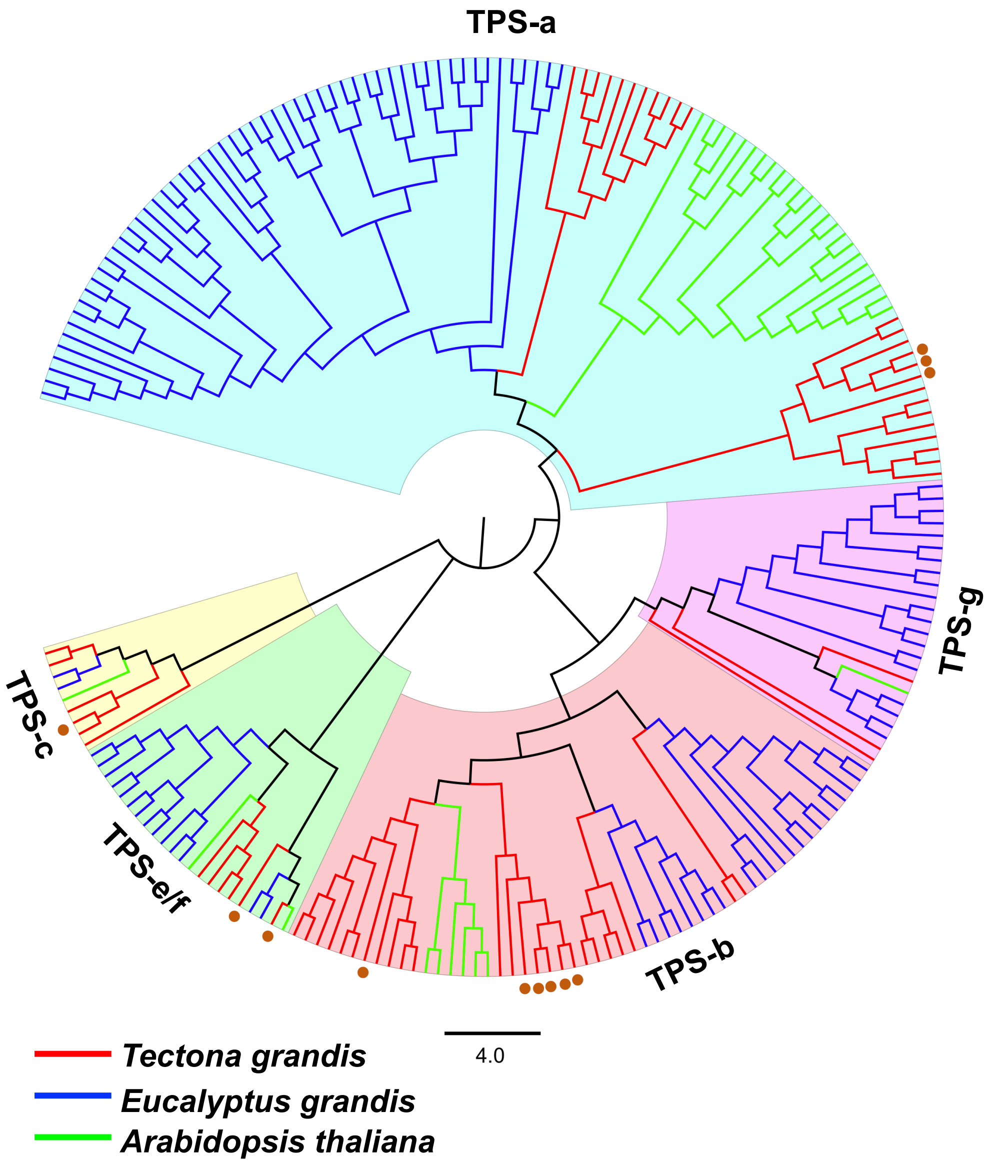

 Click here to access/download;Figure;Figure 5.pdf

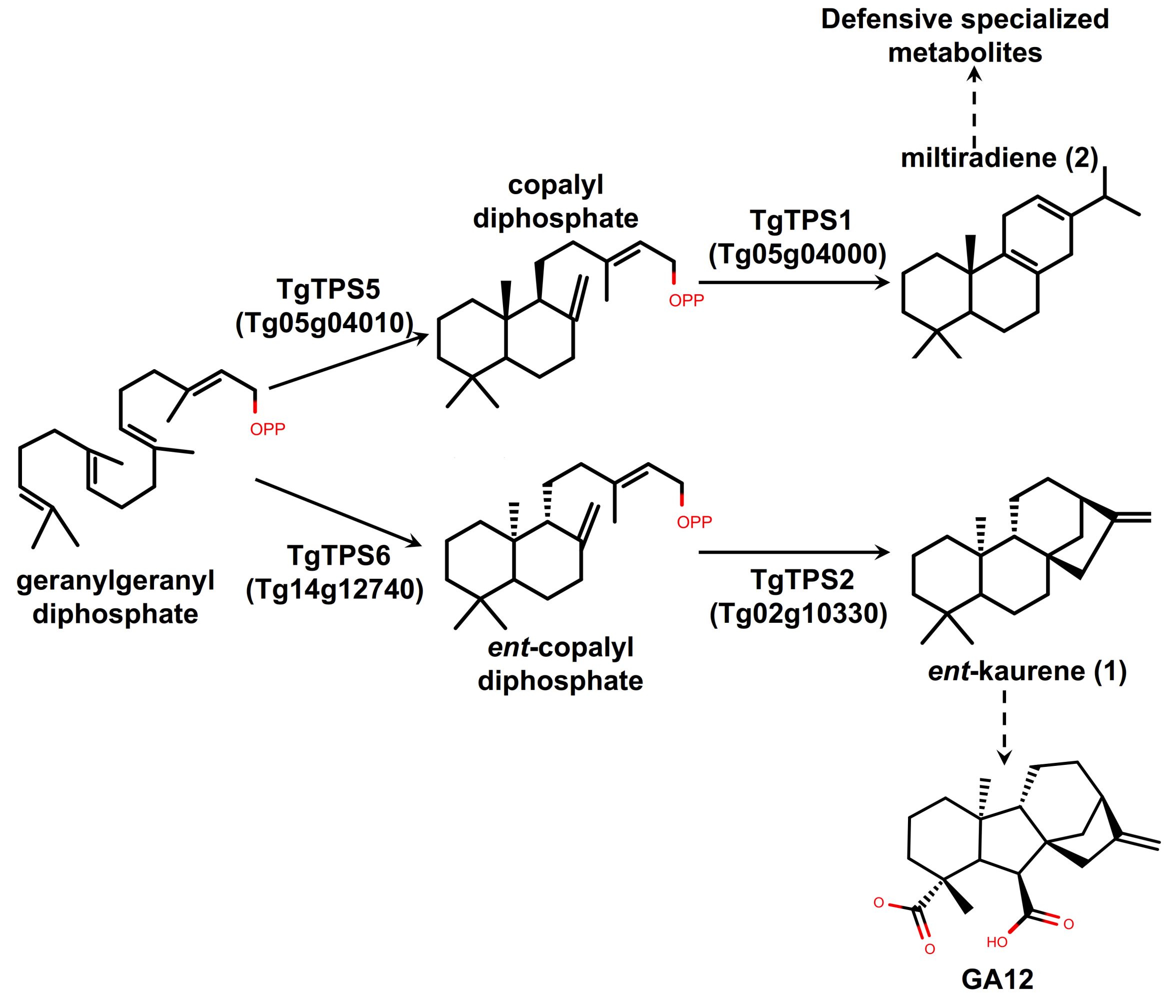

Figure 6

Click here to access/download;Figure;Figure 6.pdf

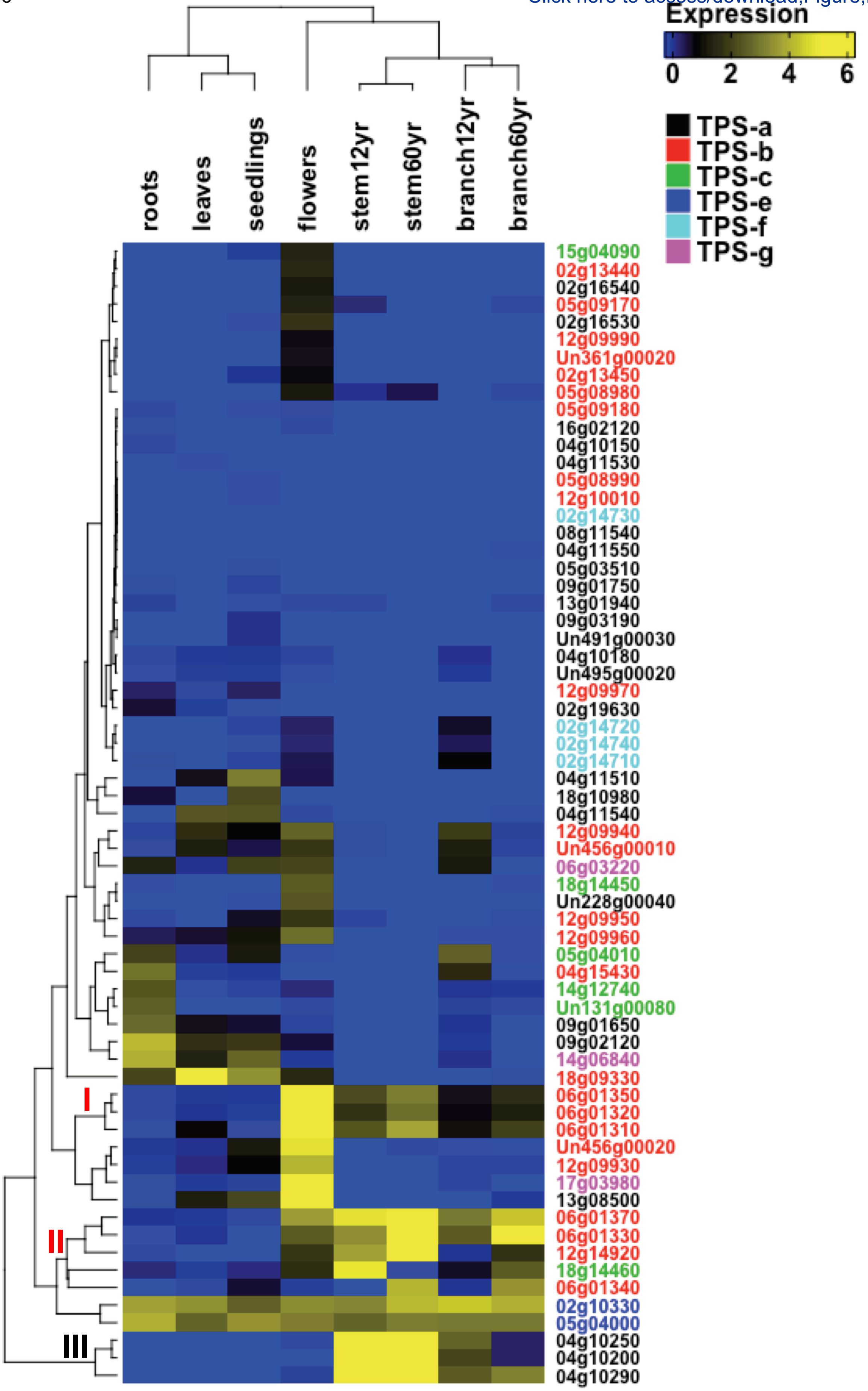

Click here to access/download;Figure;Figure 7.pdf

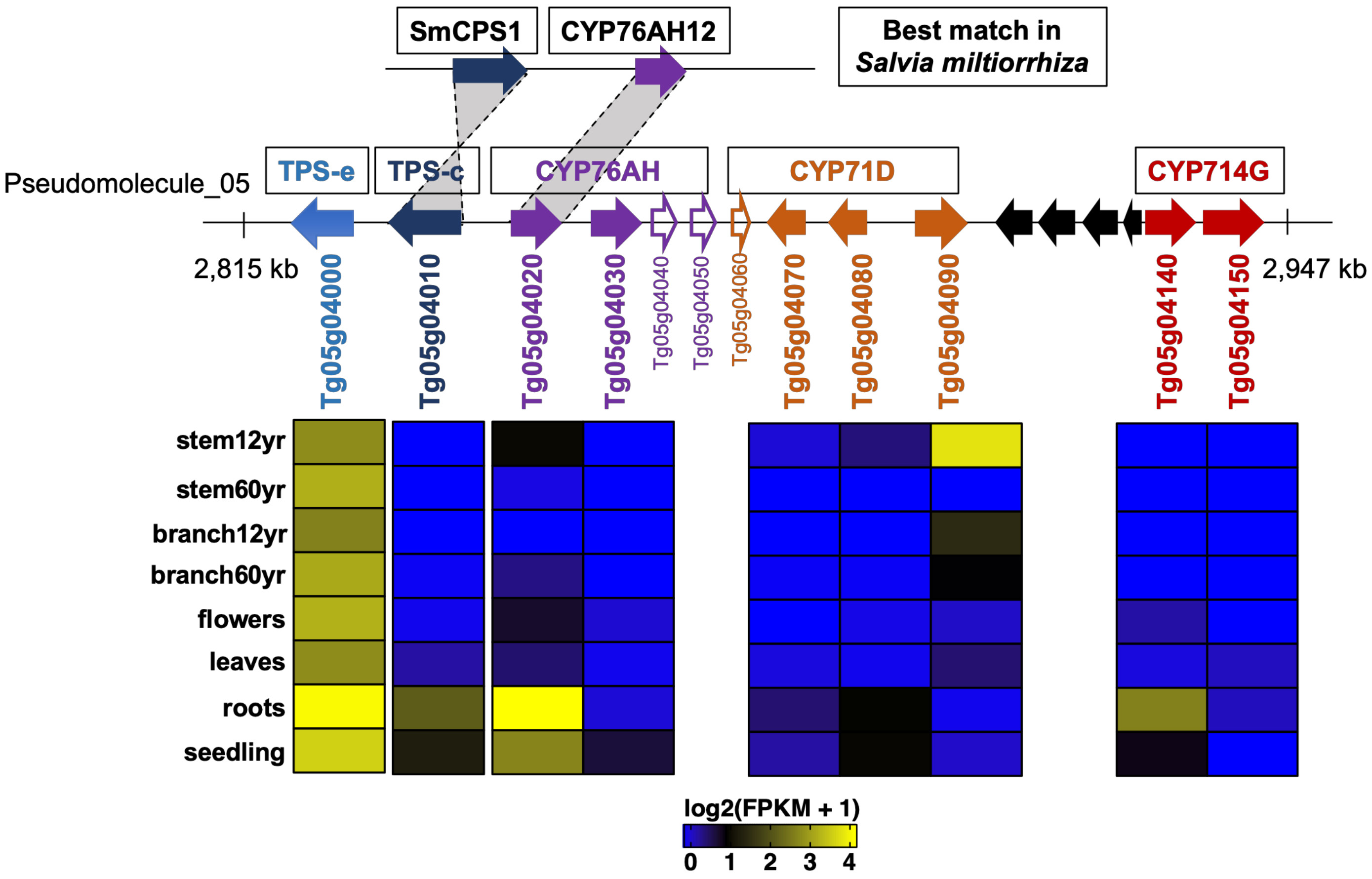

Click here to access/download
Supplementary Material
Zhao-Teak-supplemental-information-revision-Dec23.docx

Supplementary Table 1

Click here to access/download
Supplementary Material
Table S1.xlsx

Supplementary Table 2

Click here to access/download
Supplementary Material
Table S2.xlsx

Supplementary Table 3

Click here to access/download
Supplementary Material
Table S3.xlsx

Supplementary Table 4

Click here to access/download
Supplementary Material
Table S4.xlsx

Supplementary Table 5

Click here to access/download
Supplementary Material
Table S5.xlsx

Supplementary Table 6

Click here to access/download
Supplementary Material
Table S6.xlsx

Marked up version for review

Click here to access/download
Supplementary Material
Markup_version.pdf

 Click here to access/download;Personal Cover;CoverLetter_Response2Reviews.pdf

Dear Editor,

Thank you very much for handling our manuscript entitled "**A chromosomal-scale genome assembly of *Tectona grandis* enables discovery of natural product biosynthetic pathway genes key to development of sustainable teak production**" (GIGA-D-18-00458), which we have modified to “A chromosomal-scale genome assembly of *Tectona grandis* reveals the importance of tandem gene duplication and enables discovery of genes in natural product biosynthetic pathway” to better reflect its content. We appreciate the constructive comments from you and the reviewers. As you can see in our revised manuscript, we thoroughly addressed the reviewers’ comments and feel our manuscript is much improved. Here are the main revisions:

1. As suggested by you and reviewers, we added substantial description of the methods in the manuscript to help readers/researchers understand and reproduce our work.

2. A image of a teak tree is provided as Figure 1.

3. We deposited the associated datasets in GigaDB for easy access for readers/researchers.

We have also provided a point-by-point response to each of the review comments for your convenience and have uploaded a marked up version of our manuscript highlighting the revisions as well. We hope the revised manuscript is acceptable now for publication.

Thank you very much for your consideration.

Sincerely,

C. Robin Buell, Ph.D.
Michigan State University Foundation Endowed Professor & William J Beal Distinguished Faculty
Director, Plant Resilience Institute
Department of Plant Biology l MSU AgBioResearch l MSU Plant Resilience Institute
Michigan State University, S150 Plant Biology Laboratories, 612 Wilson Road, Rm 166, East Lansing MI 48824
Phone: (517) 353 5597 l Fax: (517) 353 1926 l Email: buell@msu.edu
Skype: robin.buell l buell-lab.plantbiology.msu.edu

Detailed Response to Reviewer Comments

Dear Editor and Reviewers,

Thank you very much for your time and effort to review this manuscript. We appreciate all the constructive comments and suggestions and have provided a point-by-point response below (in blue).

Both reviewers mention that more in-depth information regarding the methods is needed. Publishing articles with highly reproducible methods and data is one of our main goals at GigaScience, please take care to fully address the reviewers' comments in a revised manuscript.

Author response: We provided detailed information on methods as requested by you and reviewers. We uploaded a marked up version of the manuscript that highlightstexts revisions made in our manuscript.

On a minor note, our genome Data Notes usually show a photo of an example of the sequenced species as "Figure 1", please consider to include this as well (as part of the article, the photo will be published under our creative commons licence, please make sure you have the rights to include it under these terms).

Author response: We provided a photo of a young teak tree as Figure 1 and made changes to other figures accordingly.

Reviewer reports:
Reviewer #1: In their manuscript, Dongyan Zhao et al., present the genome assembly of Tectona grandis realised using the most recent sequencing technologies, followed by the identification and validation of genes important to wood formation, a trait of interest in teak. The manuscript is well written and the analyses appear to have been robustly conducted, but their lack of details prevents me for being more convinced. This is my only significant comment to the manuscript as it stands, it is otherwise very well written and should be a good resource for the community. As a note, I do find that the title is too boldly written considering the content presented and that the readership would gain from a more fitting title (i.e. the pathways discussed in the manuscript are well known and there is not proof as of yet that their update knowledge in teak will lead to a more sustainable teak production).

Author response: Thank you for the constructive comments. We modified the title to “A chromosomal-scale genome assembly of Tectona grandis reveals the importance of tandem gene duplication and enables discovery of genes in natural product biosynthetic pathway”.

Major comment

1) The description of the transcriptome analysis is completely missing in the main text, as are the details of which datasets were retrieved. Overall, I would wish for the supplementary document to contain the details of all analyses, including the software used, their versions and any non-default parameters - as was done for the WGD analysis. The supplementary information available from the FTP hints that more comprehensive analyses were done that is reported in the main text (e.g. classification of the gene models in different confidence bins, etc.). Such details should be made more readily visible as they would improve the manuscript' impact.

Author response: We have provided more details in the main text as suggested including adding all the versions of the software. Please see the point-to-point response in the details below.

Minor comments

1) p. 3 l.38 The number of scaffolds of the released assembly could also be given. As a stand alone figure, an N50 value is not particularly informative.

Author response: The total number of scaffolds and maximum scaffold length have been added to the main text as shown below; additional information is in Tables 1 and 2.

"The only available genome assembly for teak (hereafter referred to as the "released assembly") was completed using short-reads and low-coverage (7x) nanopore long reads [2]; while improved compared to other short-read assembled plant genomes, the released assembly is still highly fragmented, comprising 2,993 scaffolds with the maximum and N50 scaffold length of 1.7 Mb and 358 kbp, respectively."

2) p.4 l. 83 Detail the BUSCO categories (write them in full)

Author response: We have edited the as suggested.

"The representation of genic sequences in our improved assembly was confirmed by detection of 94.4% of the Benchmarking Universal Single-Copy Orthologs (BUSCO v2.0 [11]; Complete:92.3%[Single-copy:82.4%,Duplicated:9.9%], Fragmented:2.1%, Missing:5.6%, Total BUSCO groups searched:1440; Supplementary Table S1)"

3) p.5. l.88 Briefly describe the annotation process. Further in the same paragraph detail which evidences were used for Augustus (maybe discuss why Maker-P was not used) and which datasets and parameters were used for PASA2. Also provide any custom scripts in a public repository that were used for the manual curation of the genes and gene models.

Author response: The annotation process is now thoroughly described in the text.

"A custom repeat library (CRL) was generated for teak by running RepeatModeler (v1.0.8) [13], excluding protein-coding genes using ProtExcluder (v1.1) [14], and adding the Viridiplantae RepBase repeats [15]. The improved assembly was masked with the CRL using RepeatMasker (v4.0.6) with default parameters [16], which revealed that 32.02% of the improved assembly was identified as repetitive sequence, 3-fold more compared to that reported in the released assembly (11%). To generate transcript evidence for genome annotation, raw RNA-seq reads from a previous study were downloaded from NCBI (SRA SRP059970) and adapters and low-quality bases were removed using Cutadapt (v1.8.1) [17] requiring a minimum base quality of 20 and minimum size of 20-nt. The processed reads were aligned to the improved assembly using TopHat2 (v2.0.13) [18] with default parameters. Genome-guided transcript assemblies for each aligned RNA-seq library were created using Trinity (v2.2.0) [19] using the default parameters. Gene models were predicted using Augustus (v3.1) [20] by first training Augustus with the leaf RNA-seq alignments, then generating gene predictions on the hard-masked genome. The predicted gene models were refined by running PASA2 (v2.1.0) [21] using the genome-guided transcript assemblies and two rounds of annotation comparison. Genes of interest (e.g., terpene synthases as described below) were manually curated using Apollo (v1.11.8) [22]. The final working set of annotations was comprised of 31,168 loci and 46,826 gene models. Functional annotation was assigned using BLAST [23] searches against the Arabidopsis thaliana (L.) Heynh annotation (TAIR10) [24] and Swiss-Prot plant proteins (downloaded on Nov. 17, 2016), and a search against Pfam (v31) [25] using HMMER (v3.1b2) [26] with a cutoff of 1e-5. A high confidence subset of the working gene model set was identified by identifying models with an FPKM (fragments per kilobase of exon model per million reads mapped, a normalized estimation of gene expression abundance) > 0 in any of the RNA-Seq libraries or a match in Pfam (v31). The high confidence gene model set is comprised of 41,155 gene models and 39,930 loci. "

4) p5. l. 104 provide more details about what a SiZer analysis is. In general, extend the methods to contain the parameters used by the different tools, when non default (e.g. those for DupPipe line 100).

Author response: We have edited the text as suggested; see below.

"These components were further compared with results from a SiZer analysis [29] (implemented with the 'multimode' R statistical package [30]), which distinguishes true data features from noise by testing for significant increases or decreases, or no significant changes across an observed KS distribution at various bandwidths (Supplemental Information)."

“To infer WGD events in teak, we used the DupPipe pipeline with default settings [28] to analyze coding sequences representing the longest isoforms of genes (Supplemental Information).”

5) Detail how the phenylpropanoid pathway genes were identified. Similarly, detail how this was achieved for the TPSs, including tools, versions, non default parameters.

Author response: We have edited the text as suggested; see below.

“Using phenylpropanoid pathway genes in A. thaliana [31] as bait, the corresponding candidate genes in teak were identified based on orthology analysis between teak and A. thaliana using OrthoFinder v2.0 with default parameters [32].”

“A sequence similarity search using BLASTP (v2.2.31+ with default parameters) [23] was performed using the teak peptide models against a set of reference TPS peptides (Supplementary Table S5). After filtering out teak peptides shorter than 350 amino acids or having less than 30% identity to the most similar reference sequence, 65 candidate TPSs were identified, of which, 41 TPSs were located in 14 tandem clusters (Supplementary Table S6).”

6) p. 12 l. 259 "asterisks" or "stars" rather than "dots"

Author response: We have edited the text as suggested,

7) Figure 2, how was the expression calculated and what metrics is represented? Same for figure 5 and 6

Author response: We added more details on the transcription analysis in the main text as suggested.

“To generate transcript evidence for genome annotation, raw RNA-seq reads from a previous study were downloaded from NCBI (SRA SRP059970) and adapters and low-quality bases were removed using Cutadapt (v1.8.1) [17] requiring a minimum base quality of 20 and minimum size of 20-nt. The processed reads were aligned to the improved assembly using TopHat2 (v2.0.13) [18] with default parameters.”

“To better understand the potential function of these tandem gene clusters, normalized estimation of expression abundances (FPKM) of the annotated teak genes were quantified for the RNA-seq experiments (SRA SRP059970) described above using Cufflinks (v2.2.1) with default parameters [34]. Except for the 12-year-old branch (replicate 1 showed low correlation with other branch samples), the two biological replicates for other branch and stem samples showed high correlations ($r>0.94$, $p<0.0001$, Supplementary Table S4) of gene expression levels; therefore, replicate 2 for

the 12-year-old branch and one replicate for other woody tissues were used for downstream analyses."

8) In Figure 2, use the gene name described in the text in addition to the gene IDs.

Author response: Gene name abbreviations were added after the gene IDs in the figure (Now Figure 3).

9) p. 6 second paragraph and Figure 3. Discussing the gene family expansion in the light of the WGD would be of interest.

Author response: This is a good suggestion. However, discussion of gene family expansion in the light of WGD would require additional phylogenomic analyses that are beyond the scope of our current manuscript. We plan to investigate this topic in more detail within the context of additional genomes from the Lamiaceae.

10) What do the red and black bar represent in Figure 5? Add the information to the legend.

Author response: These are roman numbers (I, II, and III), which highlight the gene clusters with TPS expression in woody tissues. Information has been added to the legend and main text to clarify this.

11) In Figure 6, the coordinates as well as the scaffold should be indicated in the schematic gene representation

Author response: The scaffold number and coordinates of the region were added in the figure (now Figure 7).

12) Supplementary Table 1 should contain the BUSCO results for the released assembly (Illumina + nanopore)

Author response: We have provided the BUSCO results from the released assembly in Supplementary Table 1.

13) Supplementary Table 3 and 4; add the expression unit in the column header or as a caption.

Author response: We added the expression units in the caption as suggested. Two new supplementary tables were added, so the original Table S4 is now Table S6.

Reviewer #2: The improved version of the teak genome reported here will be a good resource for the forest tree community and for teak in particular. The genome is a great

improvement on the previous version and the methods are appropriate. Additional analysis of terpene synthase and phenylpropanoid pathway genes, particularly looking at occurence in tandem copies, highlights the utility of a contiguous, well annotated genome for furthering teak research. Overall, I found the report to be very clear and concise.

My main request to the authors is to expand the depth of the methods, particularly:

- software versions are not given for any packages used, which are typically reported for reproducibility and clarity

Author response: Software versions and other related detailed have been added as suggested.

- line 66 - "modified SNAP read mapper" - how was it modified? Can you make the modifications public?

Author response: The Hi-C scaffolding was performed by Dovetail. They have provided more details ontheir pipeline. The modification to SNAP is "the four non-genomic bases were deleted prior to the mapping." which has been added to the main text as shown below.

"Shotgun and Dovetail Hi-C library sequences were aligned to the initial assembly using a SNAP read mapper [10] where the four non-genomic bases were deleted prior to the mapping."

- line 95 - "followed by manual curation" - This is very vague - it needs a bit more description of what type of manual curtion and which genes

Author response: More details were added as suggested, which is shown below.

"Genes of interest (e.g., terpene synthases as described below) were manually curated using Apollo (v1.11.8) [22]. "

- methods for pfam domain identification are missing (hmmer version and pfam db version)

Author response: More details were added as suggested.

"Functional annotation was assigned using BLAST [23] searches against the Arabidopsis thaliana annotation (TAIR10) [24] and Swiss-Prot plant proteins (downloaded on Nov. 17, 2016), and a search against Pfam (v31) [25] using HMMER (v3.1b2) [26] with the cutoff of 1e-5."

- RNASeq mapping details are missing (what software?) and how was data normalized

Author response: More details were added as suggested.

"To generate transcript evidence for genome annotation, raw RNA-seq reads from a previous study were downloaded from NCBI (SRA SRP059970) and adapters and low-quality bases were removed using Cutadapt (v1.8.1) [17] requiring a minimum base quality of 20 and minimum size of 20-nt. The processed reads were aligned to the improved assembly using TopHat2 (v2.0.13) [18] with default parameters."

"To better understand the potential function of these tandem gene clusters, normalized estimation of expression abundances (FPKM) of the annotated teak genes were quantified for the RNA-seq experiments (SRA SRP059970) described above using Cufflinks (v2.2.1) with default parameters [34]."

- The RNASeq data used is published: Galeano E et al., "Large-scale transcriptional profiling of lignified tissues in Tectona grandis.", BMC Plant Biol, 2015 Sep 15;15:221 This paper should be cited along with the SRA accessions.

Author response: This citation was added as suggested.

- For figure 2, were expression profiles from biological replicates averaged or normalized in some other way?

Author response: More details were added in the main text to clarify this question, which is also shown below.

"Except for the 12-year-old branch (replicate 1 showed low correlation with other branch samples), the two biological replicates for other branch and stem samples showed high correlations (r>0.94, p<0.0001, Supplementary Table S4) of gene expression levels; therefore, replicate 2 for the 12-year-old branch and one replicate for other woody tissues were used for downstream analyses. "

- Expression profile colors vary from figure to figure with blue/black/yellow in figures 2 and 5, then white/red in figure 6. It would be good if they were consistent. Also, I find a two color scheme much easier to interpret over the three color blue/black/yellow.

Author Response: All figures are with the same color profiles (blue/black/yellow).

I checked a set of 4 of the Dryad files (teak_hc_models_HiC.cdna_con_sorted_modiGeneID.fa, teak_hc_models_HiC.pep_con_sorted_modiGeneID.fa, teak_hc_models_HiC_con_sorted_modiGeneID.gff, teak_tectona_grandis_26Jun2018_7GIFM_fmt_tp.fa) - all were consistently and properly formatted and matched the details in the paper.

While Dryad is great, it is still worthwhile to submit the genome and annotation to NCBI

or EMBL, where it will be more discoverable and users can take advantage of the many tools available for searching/downloading/exploring sequences.

Author Response: Thank you for your suggestion. In addition to Dryad, we have now deposited the associated data in the GigaScience database, where readers can easily obtain the files.
