# Supplementary material for: A chromosomal-scale genome assembly of Tectona grandis reveals the importance of tandem gene duplication and enables discovery of genes in natural product biosynthetic pathways
Source: Gigascience. 2019 Jan 30;8(3):giz005. doi: 10.1093/gigascience/giz005 (PMC6394206; doi:10.1093/gigascience/giz005)
Supplement: Supplemental Files [file giz005_supplemental_files.zip › Zhao-Teak-supplemental-information-revision-Dec23.docx]

**Supplementary information**

**Inference of whole-genome duplication events from coding sequences**

Synonymous substitutions are assumed to accrue at a relatively constant rate across protein-coding genes. As a result, the fixation rate of these substitutions reflects the overall mutation rate and the fraction of synonymous substitutions per synonymous site (*K*_S_) between a given pair of paralogous sequences can be used to estimate their time of duplication [1]. At the genome level, *K*_S_ distributions representing all paralogous gene pairs can reveal interesting temporal patterns of punctuated gene duplication that are indicative of ancient whole-genome duplication (WGD) events [2]. To infer WGD events in *T. grandis*, we used the DupPipe pipeline [3] with its default settings to analyze coding sequences representing the longest isoforms of genes filtered from our set of high-confidence gene models. Putative paralogous gene pairs were identified with reciprocal BLAST searches, and their synonymous divergence (*K*_S_) was estimated in DupPipe from protein-guided DNA alignments with PAML and the F3 × 4 model [4]. After removing putative paralogous gene pairs with *K*_S_ values < 0.1 and > 2 to facilitate reliable inference of WGD events [5], significant peaks in the observed *K*_S_ distribution were inferred with Gaussian mixture models, as implemented with the expectation-maximization (EM) algorithm [6] in the mixtools R package [7]. The most likely number of Gaussian components (*k*) that fit the *K*_S_ distribution was tested with parametric bootstrap analyses (100 bootstraps) of the likelihood ratio statistic using the ‘boot.comp’ function. The number of components (*k*) tested ranged from 1 to 10, and each null hypothesis of a *k-*component fit was compared with that of an alternative (*k* + 1)-component fit until the null hypothesis could not be rejected using a significance level of *α* = 0.05. The components obtained with Gaussian mixture models were further compared with results from a SiZer analysis [8], which was implemented with the ‘multimode’ R statistical package [9]. Values of *K*_S_ ≤ 2 and bandwidths ranging from *K*_S_ = 0.01 to *K*_S_ = 2 were used to identify significant (*α* = 0.05) features in the observed *K*_S_ distribution. SiZer tests for significant increases or decreases, or no significant changes across a distribution at various bandwidths to distinguish true data features from noise. Shifts from significant increases to significant decreases in a data distribution signify true peaks, and peaks from Gaussian mixture models corroborated by SiZer tests were inferred as WGD events.

**Phylogenetic tree**

Teak TPSs were identified by Blastp (v. 2.2.31+) [10] using a set of reference terpene synthases against the teak gene models. Hits with less than 350 amino acids or less than 30% identity to the reference sequences were filtered out. TPSs of *Arabidopsis thaliana* and *Eucalyptus grandis* were from Myburg *et al*. [11]. Sequences were aligned using the MUSCLE program from MEGA 7.0.26 [12]. A maximum likelihood tree was generated using Jones-Taylor-Thornton model with MEGA 7.0.26 [12]. The tree figure was generated using FigTree v1.4.3 [13].

**diTPS cloning**

From leaf tissue ground under liquid nitrogen, cDNA was prepared using the Spectrum Plant Total RNA Kit (Sigma-Aldrich, St. Louis, MO, USA), the DNA-free™ DNA Removal Kit (Thermo Fisher Scientific, Waltham, MA, USA), and the RevertAid First Strand cDNA Synthesis Kit (Thermo Fisher Scientific, Waltham, MA, USA), in that order. After first cloning into pJET1.2 (Thermo Fisher Scientific, Waltham, MA, USA), TPSs were cloned into pEAQ-HT [14] using In-Fusion® HD Cloning Plus (Takara Bio, California, USA) for transient expression in *Nicotiana benthamiana*.

Cloning primers used were: TgTPS1_Forward, ATGTTACTCGGCATCAACCTC; TgTPS1_Reverse, TCAGCTTTTTCCACTTGCATTAT; TgTPS2_Forward, ATGTCCGTTCTGCTCTCGAG; TgTPS2_Reverse, TTAATATTCATTCAGAACAATAGGTTCATG; TgTPS5_Forward, ATGGCGTCTCTATCCACTCAGATTA; TgTPS5_Reverse, TCACACAACTGGTTCGAAAAGTAC; TgTPS6_Forward, TTCTCTCTCAACACCACCACCAACA; TgTPS6_Reverse, ACATGTGCATAGTCAGAGCACTCTT; pEAQ_TgTPS1_Forward, ttctgcccaaattcgATGTTACTCGGCATCAACCTC; pEAQ_TgTPS1_Reverse, agttaaaggcctcgaTCAGCTTTTTCCACTTGC; pEAQ_TGTPS2_Forward, ttctgcccaaattcgATGTCCGTTCTGCTCTCGA; pEAQ_TgTPS2_Reverse, agttaaaggcctcgaTTAATATTCATTCAGAACAATAGG; pEAQ_TgTPS5_Forward, ttctgcccaaattcgATGGCGTCTCTATCCACTCAG; pEAQ_TgTPS5_Reverse, agttaaaggcctcgaTCACACAACTGGTTCGAAAAG; pEAQ_TgTPS6_Forward, ttctgcccaaattcgATGTCGTCTAATTTTGTTCCCA; pEAQ_TgTPS6_Reverse, agttaaaggcctcgaTCAGAGCACTCTTTCAAAGAGTA.

**Functional characterization of diTPSs by transient expression in *N. benthamiana***

DiTPS candidates and control genes were transiently expressed in *N. benthamiana* leaves as previously described [15]. To increase product accumulation, diTPSs were co-expressed with genes from earlier in the pathway, CfDXS (genbank accession: KP889115) and CfGGPPS [16,17]. Five days after infiltration, 120 mg of leaf tissue were extracted over-night in 750 µL of hexane with 2 ng/µL of 1-eicosene as an internal standard. Extracts were analyzed by GC-MS.

GC-MS analyses were performed on an Agilent 7890A GC with an Agilent VF-5ms column (30 m x 250 µm x 0.25 µm, with 10m EZ-Guard) and an Agilent 5975C detector. The inlet was set to 275°C splitless injection, He carrier gas with column flow of 1 mL/min. The oven program was 80°C hold 0.5 min, 50 °C/min to 250°C, 10°C/min to 280°C, 50°C/min to 320°C hold 4 min. All analyses were done in duplicate. Original raw GC-MS data were deposited to Zenodo (DOI: 10.5281/zenodo.1467449).

**References**

[1] S. Maere, S. De Bodt, J. Raes, T. Casneuf, M. Van Montagu, M. Kuiper, Y. Van de Peer, Modeling gene and genome duplications in eukaryotes., Proc. Natl. Acad. Sci. U. S. A. 102 (2005) 5454–9.

[2] M. Lynch, J.S. Conery, The evolutionary fate and consequences of duplicate genes, Science. 290 (2000) 1151–1155. http://www.ncbi.nlm.nih.gov/pubmed/11073452.

[3] M.S. Barker, K.M. Dlugosch, L. Dinh, R.S. Challa, N.C. Kane, M.G. King, L.H. Rieseberg, EvoPipes.net: Bioinformatic Tools for Ecological and Evolutionary Genomics, Evol. Bioinforma. 6 (2010) EBO.S5861.

[4] Z. Yang, Phylogenetic analysis by maximum likelihood, Mol. Biol. Evol. 24 (2007) 1586–1591.

[5] K. Vanneste, Y. Van de Peer, S. Maere, Inference of Genome Duplications from Age Distributions Revisited, Mol. Biol. Evol. 30 (2013) 177–190.

[6] G.J. McLachlan, D. Peel, Finite mixture models, Wiley-Interscience, New York, 2000.

[7] T. Benaglia, D. Chauveau, D.R. Hunter, D. Young, mixtools: An *R* Package for Analyzing Finite Mixture Models, J. Stat. Softw. 32 (2009) 1–29.

[8] P. Chaudhuri, J.S. Marron, SiZer for Exploration of Structures in Curves, J. Am. Stat. Assoc. 94 (1999) 807.

[9] J. Ameijeiras-Alonso, R.M. Crujeiras, A. Rodríguez-Casal, Multimode: An R Package for Mode Assessment, (2018). http://arxiv.org/abs/1803.00472.

[10] C. Camacho, G. Coulouris, V. Avagyan, N. Ma, J. Papadopoulos, K. Bealer, T.L. Madden, BLAST+: architecture and applications., BMC Bioinformatics. 10 (2009) 421.

[11] A.A. Myburg, D. Grattapaglia, G.A. Tuskan, U. Hellsten, R.D. Hayes, J. Grimwood, J. Jenkins, E. Lindquist, H. Tice, D. Bauer, D.M. Goodstein, I. Dubchak, A. Poliakov, E. Mizrachi, A.R.K. Kullan, S.G. Hussey, D. Pinard, K. van der Merwe, P. Singh, I. van Jaarsveld, O.B. Silva-Junior, R.C. Togawa, M.R. Pappas, D.A. Faria, C.P. Sansaloni, C.D. Petroli, X. Yang, P. Ranjan, T.J. Tschaplinski, C.-Y. Ye, T. Li, L. Sterck, K. Vanneste, F. Murat, M. Soler, H.S. Clemente, N. Saidi, H. Cassan-Wang, C. Dunand, C.A. Hefer, E. Bornberg-Bauer, A.R. Kersting, K. Vining, V. Amarasinghe, M. Ranik, S. Naithani, J. Elser, A.E. Boyd, A. Liston, J.W. Spatafora, P. Dharmwardhana, R. Raja, C. Sullivan, E. Romanel, M. Alves-Ferreira, C. Külheim, W. Foley, V. Carocha, J. Paiva, D. Kudrna, S.H. Brommonschenkel, G. Pasquali, M. Byrne, P. Rigault, J. Tibbits, A. Spokevicius, R.C. Jones, D.A. Steane, R.E. Vaillancourt, B.M. Potts, F. Joubert, K. Barry, G.J. Pappas, S.H. Strauss, P. Jaiswal, J. Grima-Pettenati, J. Salse, Y. Van de Peer, D.S. Rokhsar, J. Schmutz, The genome of *Eucalyptus grandis*, Nature. 510 (2014) 356–362.

[12] S. Kumar, G. Stecher, K. Tamura, MEGA7: Molecular Evolutionary Genetics Analysis Version 7.0 for Bigger Datasets, Mol. Biol. Evol. 33 (2016) 1870–1874.

[13] FigTree, http://tree.bio.ed.ac.uk/software/figtree/.

[14] F. Sainsbury, E.C. Thuenemann, G.P. Lomonossoff, pEAQ: versatile expression vectors for easy and quick transient expression of heterologous proteins in plants, Plant Biotechnol. J. 7 (2009) 682–693.

[15] S.R. Johnson, W.W. Bhat, J. Bibik, A. Turmo, B. Hamberger, B. Evolutionary Mint Genomics Consortium, Hamberger, A database-driven approach finds novel diterpene synthase activities in Lamiaceae, J. Biol. Chem. ﻿10.1074/jbc.RA118.006025

[16] I. Pateraki, J. Andersen-Ranberg, B. Hamberger, A.M. Heskes, H.J. Martens, P. Zerbe, S.S. Bach, B.L. Møller, J. Bohlmann, B. Hamberger, Manoyl Oxide (13R), the Biosynthetic Precursor of Forskolin, Is Synthesized in Specialized Root Cork Cells in *Coleus forskohlii*, Plant Physiol. 164 (2014) 1222–1236.

[17] J. Andersen-Ranberg, K.T. Kongstad, M.T. Nielsen, N.B. Jensen, I. Pateraki, S.S. Bach, B. Hamberger, P. Zerbe, D. Staerk, J. Bohlmann, B.L. Møller, B. Hamberger, Expanding the landscape of diterpene structural diversity through stereochemically controlled combinatorial biosynthesis, Angew. Chemie Int. Ed. 55 (2016) 2142–2146.

**Supplemental Figures**

**Figure S1**. Inference of ancient WGDs in *Tectona grandis*. (A) Histogram (*K*_S_ plot) showing the age distribution of putative paralogous gene pairs overlaid with mixture models of inferred WGD events. The mixture model with an inferred peak at *K*_S_ = 0.60 (red) was corroborated by the SiZer analysis (Chaudhuri and Marron, 1999), while modeled peaks at *K*_S_ = 0.22, 1.36 (blue) were not. (B) SiZer map displaying significant features in the observed *K*_S_ distribution at varying bandwidths. As indicated in the key, colors signify either a significant increase (blue), significant decrease (red), or no significant change (purple) in the data distribution.


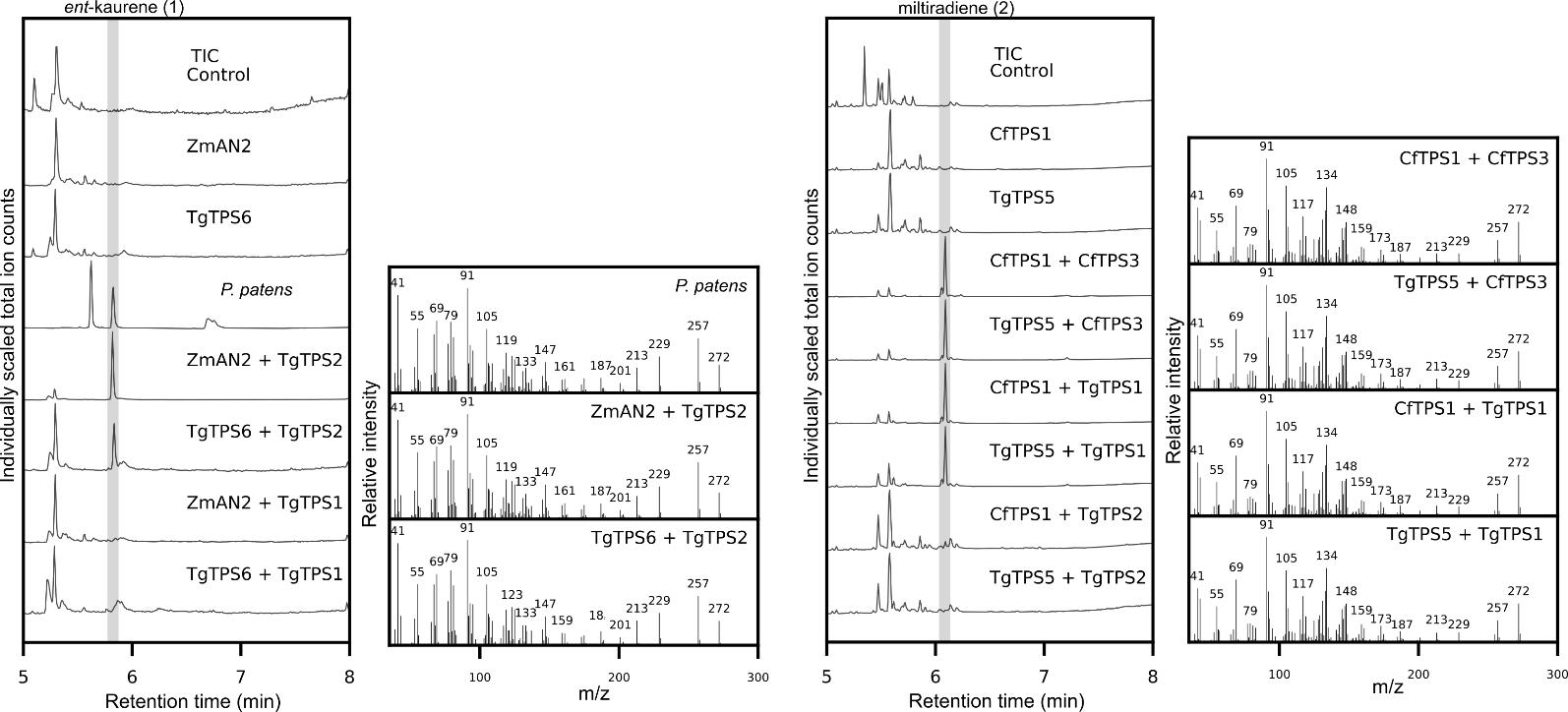


**Figure S2**. Activities of diterpene synthases after transient expression in *Nicotiana benthamiana*. On the left are total ion chromatograms of hexane extracts from plant leaves. On the right are mass spectra from individual peaks. Controls express CfDXS and CfGGPPS, but no recombinant TPS. Hexane extract from the moss *Physcomitrella patens* was used as a standard for *ent*-kaurene. *Zea mays* ZmAN2 (Genbank: AY562491) is a known *ent*-copalyl diphosphate synthase. *Coleus forskohlii* CfTPS1 (Genbank: KF444506), and CfTPS3 (Genbank: KF444508) are known (+)-copalyl diphosphate and miltiradiene synthases, respectively.
