# Supplementary material for: A chromosomal-scale genome assembly of Tectona grandis reveals the importance of tandem gene duplication and enables discovery of genes in natural product biosynthetic pathways
Source: Gigascience. 2019 Jan 30;8(3):giz005. doi: 10.1093/gigascience/giz005 (PMC6394206; doi:10.1093/gigascience/giz005)
Supplement: response_to_reviewer_comments_original_submission.pdf [file giz005_response_to_reviewer_comments_original_submission.pdf]

Author's Response To Reviewer Comments

Close

Dear Editor and Reviewers,

Thank you very much for your time and effort to review this manuscript. We appreciate all the constructive comments and suggestions and have provided a point-by-point response below (in blue).

Both reviewers mention that more in-depth information regarding the methods is needed. Publishing articles with highly reproducible methods and data is one of our main goals at GigaScience, please take care to fully address the reviewers' comments in a revised manuscript.

Author response: We provided detailed information on methods as requested by you and reviewers. We uploaded a marked up version of the manuscript that highlightstexts revisions made in our manuscript.

On a minor note, our genome Data Notes usually show a photo of an example of the sequenced species as "Figure 1", please consider to include this as well (as part of the article, the photo will be published under our creative commons licence, please make sure you have the rights to include it under these terms).

Author response: We provided a photo of a young teak tree as Figure 1 and made changes to other figures accordingly.

Reviewer reports:

Reviewer #1: In their manuscript, Dongyan Zhao et al., present the genome assembly of Tectona grandis realised using the most recent sequencing technologies, followed by the identification and validation of genes important to wood formation, a trait of interest in teak. The manuscript is well written and the analyses appear to have been robustly conducted, but their lack of details prevents me for being more convinced. This is my only significant comment to the manuscript as it stands, it is otherwise very well written and should be a good resource for the community. As a note, I do find that the title is too boldly written considering the content presented and that the readership would gain from a more fitting title (i.e. the pathways discussed in the manuscript are well known and there is not proof as of yet that their update knowledge in teak will lead to a more sustainable teak production).

Author response: Thank you for the constructive comments. We modified the title to “A chromosomal-scale genome assembly of Tectona grandis reveals the importance of tandem gene duplication and enables discovery of genes in natural product biosynthetic pathway”.

Major comment
1) The description of the transcriptome analysis is completely missing in the main text, as are

the details of which datasets were retrieved. Overall, I would wish for the supplementary document to contain the details of all analyses, including the software used, their versions and any non-default parameters - as was done for the WGD analysis. The supplementary information available from the FTP hints that more comprehensive analyses were done that is reported in the main text (e.g. classification of the gene models in different confidence bins, etc.). Such details should be made more readily visible as they would improve the manuscript' impact.

Author response: We have provided more details in the main text as suggested including adding all the versions of the software. Please see the point-to-point response in the details below.

Minor comments

1) p. 3 l.38 The number of scaffolds of the released assembly could also be given. As a stand alone figure, an N50 value is not particularly informative.

Author response: The total number of scaffolds and maximum scaffold length have been added to the main text as shown below; additional information is in Tables 1 and 2.

"The only available genome assembly for teak (hereafter referred to as the "released assembly") was completed using short-reads and low-coverage (7x) nanopore long reads [2]; while improved compared to other short-read assembled plant genomes, the released assembly is still highly fragmented, comprising 2,993 scaffolds with the maximum and N50 scaffold length of 1.7 Mb and 358 kbp, respectively."

2) p.4 l. 83 Detail the BUSCO categories (write them in full)

Author response: We have edited the as suggested.
"The representation of genic sequences in our improved assembly was confirmed by detection of 94.4% of the Benchmarking Universal Single-Copy Orthologs (BUSCO v2.0 [11]; Complete:92.3%[Single-copy:82.4%,Duplicated:9.9%], Fragmented:2.1%, Missing:5.6%, Total BUSCO groups searched:1440; Supplementary Table S1)"

3) p.5. l.88 Briefly describe the annotation process. Further in the same paragraph detail which evidences were used for Augustus (maybe discuss why Maker-P was not used) and which datasets and parameters were used for PASA2. Also provide any custom scripts in a public repository that were used for the manual curation of the genes and gene models.

Author response: The annotation process is now thoroughly described in the text.

"A custom repeat library (CRL) was generated for teak by running RepeatModeler (v1.0.8) [13], excluding protein-coding genes using ProtExcluder (v1.1) [14], and adding the Viridiplantae RepBase repeats [15]. The improved assembly was masked with the CRL using RepeatMasker (v4.0.6) with default parameters [16], which revealed that 32.02% of the improved assembly was identified as repetitive sequence, 3-fold more compared to that reported in the released assembly (11%). To generate transcript evidence for genome annotation, raw

RNA-seq reads from a previous study were downloaded from NCBI (SRA SRP059970) and adapters and low-quality bases were removed using Cutadapt (v1.8.1) [17] requiring a minimum base quality of 20 and minimum size of 20-nt. The processed reads were aligned to the improved assembly using TopHat2 (v2.0.13) [18] with default parameters. Genome-guided transcript assemblies for each aligned RNA-seq library were created using Trinity (v2.2.0) [19] using the default parameters. Gene models were predicted using Augustus (v3.1) [20] by first training Augustus with the leaf RNA-seq alignments, then generating gene predictions on the hard-masked genome. The predicted gene models were refined by running PASA2 (v2.1.0) [21] using the genome-guided transcript assemblies and two rounds of annotation comparison. Genes of interest (e.g., terpene synthases as described below) were manually curated using Apollo (v1.11.8) [22]. The final working set of annotations was comprised of 31,168 loci and 46,826 gene models. Functional annotation was assigned using BLAST [23] searches against the Arabidopsis thaliana (L.) Heynh annotation (TAIR10) [24] and Swiss-Prot plant proteins (downloaded on Nov. 17, 2016), and a search against Pfam (v31) [25] using HMMER (v3.1b2) [26] with a cutoff of 1e-5. A high confidence subset of the working gene model set was identified by identifying models with an FPKM (fragments per kilobase of exon model per million reads mapped, a normalized estimation of gene expression abundance) > 0 in any of the RNA-Seq libraries or a match in Pfam (v31). The high confidence gene model set is comprised of 41,155 gene models and 39,930 loci. ”

4) p5. l. 104 provide more details about what a SiZer analysis is. In general, extend the methods to contain the parameters used by the different tools, when non default (e.g. those for DupPipe line 100).

Author response: We have edited the text as suggested; see below.
“These components were further compared with results from a SiZer analysis [29] (implemented with the ‘multimode’ R statistical package [30]), which distinguishes true data features from noise by testing for significant increases or decreases, or no significant changes across an observed KS distribution at various bandwidths (Supplemental Information).”

“To infer WGD events in teak, we used the DupPipe pipeline with default settings [28] to analyze coding sequences representing the longest isoforms of genes (Supplemental Information).”

5) Detail how the phenylpropanoid pathway genes were identified. Similarly, detail how this was achieved for the TPSs, including tools, versions, non default parameters.

Author response: We have edited the text as suggested; see below.

“Using phenylpropanoid pathway genes in A. thaliana [31] as bait, the corresponding candidate genes in teak were identified based on orthology analysis between teak and A. thaliana using OrthoFinder v2.0 with default parameters [32].”

“A sequence similarity search using BLASTP (v2.2.31+ with default parameters) [23] was performed using the teak peptide models against a set of reference TPS peptides (Supplementary Table S5). After filtering out teak peptides shorter than 350 amino acids or

having less than 30% identity to the most similar reference sequence, 65 candidate TPSs were identified, of which, 41 TPSs were located in 14 tandem clusters (Supplementary Table S6)."

6) p. 12 l. 259 "asterisks" or "stars" rather than "dots"

Author response: We have edited the text as suggested,

7) Figure 2, how was the expression calculated and what metrics is represented? Same for figure 5 and 6

Author response: We added more details on the transcription analysis in the main text as suggested.

"To generate transcript evidence for genome annotation, raw RNA-seq reads from a previous study were downloaded from NCBI (SRA SRP059970) and adapters and low-quality bases were removed using Cutadapt (v1.8.1) [17] requiring a minimum base quality of 20 and minimum size of 20-nt. The processed reads were aligned to the improved assembly using TopHat2 (v2.0.13) [18] with default parameters."

"To better understand the potential function of these tandem gene clusters, normalized estimation of expression abundances (FPKM) of the annotated teak genes were quantified for the RNA-seq experiments (SRA SRP059970) described above using Cufflinks (v2.2.1) with default parameters [34]. Except for the 12-year-old branch (replicate 1 showed low correlation with other branch samples), the two biological replicates for other branch and stem samples showed high correlations ($r>0.94$, $p<0.0001$, Supplementary Table S4) of gene expression levels; therefore, replicate 2 for the 12-year-old branch and one replicate for other woody tissues were used for downstream analyses."

8) In Figure 2, use the gene name described in the text in addition to the gene IDs.

Author response: Gene name abbreviations were added after the gene IDs in the figure (Now Figure 3).

9) p. 6 second paragraph and Figure 3. Discussing the gene family expansion in the light of the WGD would be of interest.

Author response: This is a good suggestion. However, discussion of gene family expansion in the light of WGD would require additional phylogenomic analyses that are beyond the scope of our current manuscript. We plan to investigate this topic in more detail within the context of additional genomes from the Lamiaceae.

10) What do the red and black bar represent in Figure 5? Add the information to the legend.

Author response: These are roman numbers (I, II, and III), which highlight the gene clusters with TPS expression in woody tissues. Information has been added to the legend and main text to clarify this.

11) In Figure 6, the coordinates as well as the scaffold should be indicated in the schematic gene representation

Author response: The scaffold number and coordinates of the region were added in the figure (now Figure 7).

12) Supplementary Table 1 should contain the BUSCO results for the released assembly (Illumina + nanopore)

Author response: We have provided the BUSCO results from the released assembly in Supplementary Table 1.

13) Supplementary Table 3 and 4; add the expression unit in the column header or as a caption.

Author response: We added the expression units in the caption as suggested. Two new supplementary tables were added, so the original Table S4 is now Table S6.

Reviewer #2: The improved version of the teak genome reported here will be a good resource for the forest tree community and for teak in particular. The genome is a great improvement on the previous version and the methods are appropriate. Additional analysis of terpene synthase and phenylpropanoid pathway genes, particularly looking at occurence in tandem copies, highlights the utility of a contiguous, well annotated genome for furthering teak research. Overall, I found the report to be very clear and concise.

My main request to the authors is to expand the depth of the methods, particularly:

- software versions are not given for any packages used, which are typically reported for reproducibility and clarity
Author response: Software versions and other related detailed have been added as suggested.

- line 66 - "modified SNAP read mapper" - how was it modified? Can you make the modifications public?

Author response: The Hi-C scaffolding was performed by Dovetail. They have provided more details ontheir pipeline. The modification to SNAP is “the four non-genomic bases were deleted prior to the mapping.” which has been added to the main text as shown below.
“Shotgun and Dovetail Hi-C library sequences were aligned to the initial assembly using a SNAP read mapper [10] where the four non-genomic bases were deleted prior to the mapping.”

- line 95 - "followed by manual curation" - This is very vague - it needs a bit more description of what type of manual curtion and which genes

Author response: More details were added as suggested, which is shown below.

"Genes of interest (e.g., terpene synthases as described below) were manually curated using Apollo (v1.11.8) [22]. "

- methods for pfam domain identification are missing (hmmer version and pfam db version)

Author response: More details were added as suggested.

"Functional annotation was assigned using BLAST [23] searches against the Arabidopsis thaliana annotation (TAIR10) [24] and Swiss-Prot plant proteins (downloaded on Nov. 17, 2016), and a search against Pfam (v31) [25] using HMMER (v3.1b2) [26] with the cutoff of 1e-5."

- RNASeq mapping details are missing (what software?) and how was data normalized

Author response: More details were added as suggested.

"To generate transcript evidence for genome annotation, raw RNA-seq reads from a previous study were downloaded from NCBI (SRA SRP059970) and adapters and low-quality bases were removed using Cutadapt (v1.8.1) [17] requiring a minimum base quality of 20 and minimum size of 20-nt. The processed reads were aligned to the improved assembly using TopHat2 (v2.0.13) [18] with default parameters."

"To better understand the potential function of these tandem gene clusters, normalized estimation of expression abundances (FPKM) of the annotated teak genes were quantified for the RNA-seq experiments (SRA SRP059970) described above using Cufflinks (v2.2.1) with default parameters [34]."

- The RNASeq data used is published: Galeano E et al., "Large-scale transcriptional profiling of lignified tissues in Tectona grandis.", BMC Plant Biol, 2015 Sep 15;15:221

This paper should be cited along with the SRA accessions.

Author response: This citation was added as suggested.

- For figure 2, were expression profiles from biological replicates averaged or normalized in some other way?

Author response: More details were added in the main text to clarify this question, which is also shown below.

"Except for the 12-year-old branch (replicate 1 showed low correlation with other branch samples), the two biological replicates for other branch and stem samples showed high correlations ($r>0.94$, $p<0.0001$, Supplementary Table S4) of gene expression levels; therefore, replicate 2 for the 12-year-old branch and one replicate for other woody tissues were used for downstream analyses. "

- Expression profile colors vary from figure to figure with blue/black/yellow in figures 2 and 5, then white/red in figure 6. It would be good if they were consistent. Also, I find a two color scheme much easier to interpret over the three color blue/black/yellow.

Author Response: All figures are with the same color profiles (blue/black/yellow).

I checked a set of 4 of the Dryad files (teak_hc_models_HiC.cdna_con_sorted_modiGeneID.fa, teak_hc_models_HiC.pep_con_sorted_modiGeneID.fa, teak_hc_models_HiC_con_sorted_modiGeneID.gff, teak_tectona_grandis_26Jun2018_7GlFM_fmt_tp.fa) - all were consistently and properly formatted and matched the details in the paper.

While Dryad is great, it is still worthwhile to submit the genome and annotation to NCBI or EMBL, where it will be more discoverable and users can take advantage of the many tools available for searching/downloading/exploring sequences.

Author Response: Thank you for your suggestion. In addition to Dryad, we have now deposited the associated data in the GigaScience database, where readers can easily obtain the files.

Close
