# Supplementary material for: A chromosomal-scale genome assembly of Tectona grandis reveals the importance of tandem gene duplication and enables discovery of genes in natural product biosynthetic pathways
Source: Gigascience. 2019 Jan 30;8(3):giz005. doi: 10.1093/gigascience/giz005 (PMC6394206; doi:10.1093/gigascience/giz005)
Supplement: reviewer_1_report_original_submission -- Nicolas Delhomme, Ph. D. rer. nat. — 12/10/2018 Reviewed [file giz005_reviewer_1_report_original_submission.pdf]

**Reviewer Report**

**Title: A chromosomal-scale genome assembly of Tectona grandis reveals the importance of tandem gene duplication and enables discovery of genes in natural product biosynthetic pathways**

**Version: Original Submission** **Date:** 12/10/2018

**Reviewer name: Nicolas Delhomme, Ph. D. rer. nat.**

**Reviewer Comments to Author:**

In their manuscript, Dongyan Zhao et al., present the genome assembly of Tectona grandis realised using the most recent sequencing technologies, followed by the identification and validation of genes important to wood formation, a trait of interest in teak. The manuscript is well written and the analyses appear to have been robustly conducted, but their lack of details prevents me for being more convinced. This is my only significant comment to the manuscript as it stands, it is otherwise very well written and should be a good resource for the community. As a note, I do find that the title is too boldly written considering the content presented and that the readership would gain from a more fitting title (i.e. the pathways discussed in the manuscript are well known and there is not proof as of yet that their update knowledge in teak will lead to a more sustainable teak production).

Major comment

1) The description of the transcriptome analysis is completely missing in the main text, as are the details of which datasets were retrieved. Overall, I would wish for the supplementary document to contain the details of all analyses, including the software used, their versions and any non-default parameters - as was done for the WGD analysis. The supplementary information available from the FTP hints that more comprehensive analyses were done that is reported in the main text (e.g. classification of the gene models in different confidence bins, etc.). Such details should be made more readily visible as they would improve the manuscript' impact.

Minor comments

1) p. 3 l.38 The number of scaffolds of the released assembly could also be given. As a stand alone figure, an N50 value is not particularly informative.
2) p.4 l. 83 Detail the BUSCO categories (write them in full)
3) p.5. l.88 Briefly describe the annotation process. Further in the same paragraph detail which evidences were used for Augustus (maybe discuss why Maker-P was not used) and which datasets and parameters were used for PASA2. Also provide any custom scripts in a public repository that were used for the manual curation of the genes and gene models.
4) p5. l. 104 provide more details about what a SiZer analysis is. In general, extend the methods to contain the parameters used by the different tools, when non default (e.g. those for DupPipe line 100).
5) Detail how the phenylpropanoid pathway genes were identified. Similarly, detail how this was achieved for the TPSs, including tools, versions, non default parameters.
6) p. 12 l. 259 "asterisks" or "stars" rather than "dots"
7) Figure 2, how was the expression calculated and what metrics is represented? Same for figure 5 and 6
8) In Figure 2, use the gene name described in the text in addition to the gene IDs.

9) p. 6 second paragraph and Figure 3. Discussing the gene family expansion in the light of the WGD would be of interest.
10) What do the red and black bar represent in Figure 5? Add the information to the legend.
11) In Figure 6, the coordinates as well as the scaffold should be indicated in the schematic gene representation
12) Supplementary Table 1 should contain the BUSCO results for the released assembly (Illumina + nanopore)
13) Supplementary Table 3 and 4; add the expression unit in the column header or as a caption.
This is a signed review by Dr. Nicolas Delhomme, researcher at the Swedish University for Agricultural Sciences, and manager of the bioinformatics facility at the UmeÃ¥ Plant Science Centre.

**Methods**

Are the methods appropriate to the aims of the study, are they well described, and are necessary controls included? Choose an item.

**Conclusions**

Are the conclusions adequately supported by the data shown? Choose an item.

**Reporting Standards**

Does the manuscript adhere to the journal's guidelines on minimum standards of reporting? Choose an item.

Choose an item.

**Statistics**

Are you able to assess all statistics in the manuscript, including the appropriateness of statistical tests used? Choose an item.

**Quality of Written English**

Please indicate the quality of language in the manuscript: Choose an item.

**Declaration of Competing Interests**

Please complete a declaration of competing interests, considering the following questions:

- Have you in the past five years received reimbursements, fees, funding, or salary from an organisation that may in any way gain or lose financially from the publication of this manuscript, either now or in the future?
- Do you hold any stocks or shares in an organisation that may in any way gain or lose financially from the publication of this manuscript, either now or in the future?

- Do you hold or are you currently applying for any patents relating to the content of the manuscript?
- Have you received reimbursements, fees, funding, or salary from an organization that holds or has applied for patents relating to the content of the manuscript?
- Do you have any other financial competing interests?
- Do you have any non-financial competing interests in relation to this paper?

If you can answer no to all of the above, write 'I declare that I have no competing interests' below. If your reply is yes to any, please give details below.

I declare that I have no competing interests

I agree to the open peer review policy of the journal. I understand that my name will be included on my report to the authors and, if the manuscript is accepted for publication, my named report including any attachments I upload will be posted on the website along with the authors' responses. I agree for my report to be made available under an Open Access Creative Commons CC-BY license (http://creativecommons.org/licenses/by/4.0/). I understand that any comments which I do not wish to be included in my named report can be included as confidential comments to the editors, which will not be published.

I agree to the open peer review policy of the journal

To further support our reviewers, we have joined with Publons, where you can gain additional credit to further highlight your hard work (see: https://publons.com/journal/530/gigascience). On publication of this paper, your review will be automatically added to Publons, you can then choose whether or not to claim your Publons credit. I understand this statement.

Yes Choose an item.
