# Supplementary material for: A chromosomal-scale genome assembly of Tectona grandis reveals the importance of tandem gene duplication and enables discovery of genes in natural product biosynthetic pathways
Source: Gigascience. 2019 Jan 30;8(3):giz005. doi: 10.1093/gigascience/giz005 (PMC6394206; doi:10.1093/gigascience/giz005)
Supplement: reviewer_2_report_original_submission -- Meg Staton — 12/11/2018 Reviewed [file giz005_reviewer_2_report_original_submission.pdf]

Reviewer Report

Title: A chromosomal-scale genome assembly of Tectona grandis reveals the importance of tandem gene duplication and enables discovery of genes in natural product biosynthetic pathways

Version: Original Submission **Date:** 12/11/2018

Reviewer name: Meg Staton

Reviewer Comments to Author:

The improved version of the teak genome reported here will be a good resource for the forest tree community and for teak in particular. The genome is a great improvement on the previous version and the methods are appropriate. Additional analysis of terpene synthase and phenylpropanoid pathway genes, particularly looking at occurence in tandem copies, highlights the utility of a contiguous, well annotated genome for furthering teak research. Overall,I found the report to be very clear and concise.
My main request to the authors is to expand the depth of the methods, particularly:
- software versions are not given for any packages used, which are typically reported for reproducibility and clarity
- line 66 - "modified SNAP read mapper" - how was it modified? Can you make the modifications public?
- line 95 - "followed by manual curation" - This is very vague - it needs a bit more description of what type of manual curtion and which genes
- methods for pfam domain identification are missing (hmmer version and pfam db version)
- RNASeq mapping details are missing (what software?) and how was data normalized
- The RNASeq data used is published: Galeano E et al., "Large-scale transcriptional profiling of lignified tissues in Tectona grandis.", BMC Plant Biol, 2015 Sep 15;15:221
This paper should be cited along with the SRA accessions.
- For figure 2, were expression profiles from biological replicates averaged or normalized in some other way?
- Expression profile colors vary from figure to figure with blue/black/yellow in figures 2 and 5, then white/red in figure 6. It would be good if they were consistent. Also, I find a two color scheme much easier to interpret over the three color blue/black/yellow.
I checked a set of 4 of the Dryad files (teak_hc_models_HiC.cdna_con_sorted_modiGeneID.fa, teak_hc_models_HiC.pep_con_sorted_modiGeneID.fa, teak_hc_models_HiC_con_sorted_modiGeneID.gff, teak_tectona_grandis_26Jun2018_7GlFM_fmt_tp.fa) - all were consistently and properly formatted and matched the details in the paper.
While Dryad is great, it is still worthwhile to submit the genome and annotation to NCBI or EMBL, where it will be more discoverable and users can take advantage of the many tools available for searching/downloading/exploring sequences.

Methods

Are the methods appropriate to the aims of the study, are they well described, and are necessary controls included? Choose an item.

Conclusions

Are the conclusions adequately supported by the data shown? Choose an item.

Reporting Standards

Does the manuscript adhere to the journal's guidelines on minimum standards of reporting? Choose an item.

Choose an item.

Statistics

Are you able to assess all statistics in the manuscript, including the appropriateness of statistical tests used? Choose an item.

Quality of Written English

Please indicate the quality of language in the manuscript: Choose an item.

Declaration of Competing Interests

Please complete a declaration of competing interests, considering the following questions:

- Have you in the past five years received reimbursements, fees, funding, or salary from an organisation that may in any way gain or lose financially from the publication of this manuscript, either now or in the future?
- Do you hold any stocks or shares in an organisation that may in any way gain or lose financially from the publication of this manuscript, either now or in the future?
- Do you hold or are you currently applying for any patents relating to the content of the manuscript?
- Have you received reimbursements, fees, funding, or salary from an organization that holds or has applied for patents relating to the content of the manuscript?
- Do you have any other financial competing interests?
- Do you have any non-financial competing interests in relation to this paper?

If you can answer no to all of the above, write 'I declare that I have no competing interests' below. If your reply is yes to any, please give details below.

I declare that I have no competing interests

I agree to the open peer review policy of the journal. I understand that my name will be included on my report to the authors and, if the manuscript is accepted for publication, my named report including any attachments I upload will be posted on the website along with the authors' responses. I agree for my

report to be made available under an Open Access Creative Commons CC-BY license (http://creativecommons.org/licenses/by/4.0/). I understand that any comments which I do not wish to be included in my named report can be included as confidential comments to the editors, which will not be published.

I agree to the open peer review policy of the journal

To further support our reviewers, we have joined with Publons, where you can gain additional credit to further highlight your hard work (see: https://publons.com/journal/530/gigascience). On publication of this paper, your review will be automatically added to Publons, you can then choose whether or not to claim your Publons credit. I understand this statement.

Yes Choose an item.
